# Triple Cross‐linked Dynamic Responsive Hydrogel Loaded with Selenium Nanoparticles for Modulating the Inflammatory Microenvironment via PI3K/Akt/NF‐κB and MAPK Signaling Pathways

**DOI:** 10.1002/advs.202303167

**Published:** 2023-09-22

**Authors:** Shuangqing Wang, Yanhong Liu, Qianwen Sun, Bowen Zeng, Chao Liu, Liming Gong, Hao Wu, Liqing Chen, Mingji Jin, Jianpeng Guo, Zhonggao Gao, Wei Huang

**Affiliations:** ^1^ State Key Laboratory of Bioactive Substance and Function of Natural Medicines Institute of Materia Medica Chinese Academy of Medical Sciences and Peking Union Medical College Beijing 100050 China; ^2^ Beijing Key Laboratory of Drug Delivery Technology and Novel Formulations Department of Pharmaceutics Institute of Materia Medica Chinese Academy of Medical Sciences and Peking Union Medical College Beijing 100050 China; ^3^ Key Laboratory of Natural Medicines of the Changbai Mountain Ministry of Education College of Pharmacy Yanbian University Yanji Jilin Province 133002 China

**Keywords:** injectable hydrogel, reactive oxygen species, rheumatoid arthritis, selenium nanoparticles, wound healing

## Abstract

Modulating the inflammatory microenvironment can inhibit the process of inflammatory diseases (IDs). A tri‐cross‐linked inflammatory microenvironment‐responsive hydrogel with ideal mechanical properties achieves triggerable and sustained drug delivery and regulates the inflammatory microenvironment. Here, this study develops an inflammatory microenvironment‐responsive hydrogel (OD‐PP@SeNPs) composed of phenylboronic acid grafted polylysine (PP), oxidized dextran (OD), and selenium nanoparticles (SeNPs). The introduction of SeNPs as initiators and nano‐fillers into the hydrogel results in extra cross‐linking of the polymer network through hydrogen bonding. Based on Schiff base bonds, Phenylboronate ester bonds, and hydrogen bonds, a reactive oxygen species (ROS)/pH dual responsive hydrogel with a triple‐network is achieved. The hydrogel has injectable, self‐healing, adhesion, outstanding flexibility, suitable swelling capacity, optimal biodegradability, excellent stimuli‐responsive active substance release performance, and prominent biocompatibility. Most importantly, the hydrogel with ROS scavenging and pH‐regulating ability protects cells from oxidative stress and induces macrophages into M2 polarization to reduce inflammatory cytokines through PI3K/AKT/NF‐κB and MAPK pathways, exerting anti‐inflammatory effects and reshaping the inflammatory microenvironment, thereby effectively treating typical IDs, including *S. aureus* infected wound and rheumatoid arthritis in rats. In conclusion, this dynamically responsive injectable hydrogel with a triple‐network structure provides an effective strategy to treat IDs, holding great promise in clinical application.

## Graphical abstract

The tri‐cross‐linked dynamic response hydrogel with ROS scavenging and pH‐regulating ability protects cells from oxidative stress and induces macrophages into M2 polarization to reduce inflammatory cytokines through PI3K/AKT/NF‐κB and MAPK pathways, exerting anti‐inflammatory effects and reshaping the inflammatory microenvironment, thereby effectively treating typical IDs, including *S. aureus* infected wound and rheumatoid arthritis in rats.

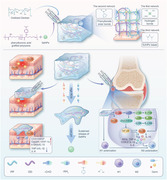

## 1 Introduction

Inflammatory diseases (IDs) are a general term that covers all diseases in which chronic inflammation perform as the primary manifestation of pathogenesis.^[^ ^1^, ^2^ ^]^ The inflammatory response is redness, pain, swelling, and organ dysfunction.^[^ ^3^, ^4^ ^]^ Thus, controlling the development of inflammation processes as early as possible is a promising strategy for treatment IDs. However, traditional treatment is based on anti‐inflammatory and immunosuppressive drugs, which are palliative and provide short‐term relief with serious side effects.^[^ ^5^, ^6^ ^]^ Therefore, the development of reliable, non‐toxic, long‐lasting, and low‐cost treatments have great potential in the pharmacological market.

Delivering drugs via biomaterials provides an alternative strategy for IDs treatment. Over the past few decades, synthetic hydrogels with a hydrophilic polymer network structure resembling biological soft tissues have made exciting advances in drug delivery.^[^ ^7^, ^8^, ^9^ ^]^ Hydrogels have been widely used as a treatment for IDs due to their remarkable biocompatibility, tunable biodegradability, and controlled mechanical strength. Whereas, traditional hydrogels have single characteristics, low mechanical properties, high porosity (resulting in rapid gel erosion and drug diffusion), and uncontrollable drug release in pathological environments, greatly limiting their further development. Various functional hydrogels have been developed in recent years, such as injectable, self‐healing, and stimulus‐responsive.^[^ ^10^, ^11^ ^]^ Stimulus‐responsive hydrogels, also known as “smart hydrogels”, have become a hot research topic due to their potential applications in biomedicine, responding to external microenvironmental stimuli such as glucose, temperature, pH, reactive oxygen species (ROS), and enzymes.^[^ ^12^ ^]^ Notably, the inflammatory microenvironment is primarily characterized by increased O_2_ demand and excessive ROS production, which in turn plays a further crucial role in inflammation exacerbation. For the treatment IDs, pH and ROS are appropriate triggers.^[^ ^13^, ^14^ ^]^ Therefore, constructing multifunctional hydrogels with multiple dynamic responses from existing biomaterials is a key challenge.

The functional properties of the hydrogels are vitally dependent on the polymeric composition and the interaction within the network.^[^ ^15^, ^16^, ^17^ ^]^ The formation and dissociation of Phenylboronate ester bonds, Schiff base bonds, Disulfide bonds, Acylhydrazone bonds, and Diels‐Alder reactions exhibit unprecedented versatility by simultaneously exhibiting pH and ROS sensitivity.^[^ ^18^, ^19^, ^20^ ^]^ As a class of Lewis acid, phenylboronic acid (PBA) can combine with compounds carrying catechol to form a Phenylboronate ester bonds, which is a reversible covalent bond.^[^ ^21^ ^]^ As a biodegradable biopolymer, polylysine (PLL) has abundant primary amine groups, low cost, and non‐toxic advantages.^[^ ^22^, ^23^ ^]^ Therefore, PBA grafted PLL (PP) combines with 1,2‐diol‐containing polymers to form Phenylboronate ester bonds. Dextran is a polysaccharide often studied for hydrogel preparation and has excellent biocompatibility and biodegradability.^[^ ^24^ ^]^ The dextran structure is rich in adjacent hydroxyl groups, which could be oxidized to generate aldehyde groups (–CHO).^[^ ^25^, ^26^, ^27^ ^]^ The aldehyde groups on oxidized dextran (OD) can form reversible covalent Schiff base bonds with the amino groups on PP structure chain, which have the characteristics of high reactivity and controllable cross‐linking degree. At the same time, the unoxidized 1,2‐vicinal diol on OD can form a Phenylboronate ester bonds with the phenylboronic acid group on PP. A novel double cross‐linked hydrogel (OD‐PP hydrogel) was developed based on Schiff base bonds and Phenylboronate ester bonds, which can respond to low pH and high ROS, reshape the inflammatory microenvironment, and promote the recovery of IDs. It has been reported that hydrogels containing multiple covalent cross‐links show good mechanical properties.^[^ ^28^, ^29^, ^30^ ^]^ Hydrogels with desirable mechanical properties need to be further explored for their functionality and to broaden the scope of their applications.

The hydrogels based on reversible covalent chemical bonds have been extensively examined for on‐demand therapeutic delivery of therapeutic agents and cells to treat different IDs. Selenium (Se) is an essential micronutrient that plays a vital role in regulating the immune response. However, the toxicological safety of organic/inorganic selenium compounds limits the applications on multiple diseases treatments.^[^ ^31^ ^]^ Selenium nanoparticles (SeNPs) have received extensive attention due to their excellent biocompatibility, antibacterial, antiviral, and anticancer activities, and notable antioxidant and anti‐inflammatory properties.^[^ ^32^, ^33^, ^34^, ^35^, ^36^, ^37^ ^]^ SeNPs triggers intracellular ROS overproduction to induce cell and bacterial apoptosis. Nevertheless, SeNPs have significantly emerged with pro‐oxidant and antioxidant potential dependent on subsequent duration, dose, frequency, and oxidation state.^[^ ^38^ ^]^ Given that SeNPs have non‐negligible concentration‐dependent therapeutic effects,^[^ ^39^ ^]^ it is a promising strategy to encapsulate SeNPs in a double covalently cross‐linked hydrogel. In an acidic environment with high ROS, the hydrogels based on Schiff base bonds and Phenylboronate ester bonds can be controllably decomposed and release SeNPs on demand, and maintain the SeNPs concentration at the desired level for clinical use. SeNPs endow additional properties to the hydrogel, such as antioxidant, anti‐inflammatory, and antibacterial. At the same time, the SeNPs work dually as initiators and nano‐fillers, and the polymer network interacts through hydrogen bonds to achieve triple cross‐linking. The addition of SeNPs significantly enhances the mechanical properties of the hydrogels. Tang et al. prepared a multifunctional hydrogel (CCOD‐MgO) by adding MgO to catechol‐modified chitosan and oxidized dextran (OD). CCOD‐MgO exhibits excellent self‐healing, hemostatic function, and low swelling rate.^[^ ^40^ ^]^ Carcia‐Astrain et al. developed a gelatin hydrogel using silver nanoparticles (AgNPs) as a cross‐linking agent and operating carrier. The addition of AgNPs significantly improved the mechanical properties of the hydrogels.^[^ ^41^ ^]^

Although some antioxidant properties of SeNPs have been reported, the specific mechanism is not fully elucidated. Further, exploring the anti‐inflammatory mechanism of SeNPs hydrogel will contribute to reshaping the inflammatory microenvironment and fundamentally curing IDs. Inflammatory sites are characterized by inflammatory cell infiltration and high levels of inflammatory cytokines.^[^ ^42^ ^]^ In this process, infiltrating inflammatory cells (neutrophils and macrophages) produce high levels of ROS. Excessive ROS will activate nuclear factor kappa‐B (NF‐κB) and mitogen‐activated protein kinase (MAPK) pathways, leading to the release of tumor necrosis factor‐α (TNF‐α), interleukin‐1β (IL‐1β) and interleukin‐6 (IL‐6).^[^ ^43^ ^]^ This causes oxidative stress in surrounding cells, leading to healthy cell apoptosis and eventually has a degenerative effect on the inflammatory lesions. Therefore, the removal of excess ROS is particularly critical in the treatment of IDs. It has been reported that Se can alleviate the inflammatory reaction by suppressing the activation of the NF‐κB and MAPK signaling pathways.^[^ ^44^ ^]^ The phosphatidylinositol 3‐kinase (PI3K) is a signaling enzyme complex that combines with protein kinase B (Akt) to regulate cellular metabolism. PI3K‐AKT modulates NF‐κB transcriptional activity and stimulates the NF‐κB signaling pathway, which may lead to an inflammatory response and the release of proinflammatory cytokines.^[^ ^45^ ^]^ As an upstream signal of NF‐κB and MAPK, whether PI3K/Akt is involved in regulating the inflammatory microenvironment by SeNPs hydrogel is not clarified.

In light of the above considerations, we present a high‐performance biomedical hydrogel with triple cross‐linking, namely, OD‐PP@SeNPs, which is injectable, self‐healing, strongly adhesive to tissues, and biodegradable. The critical gelation pathway is to establish triple dynamic cross‐linking among PBA grafted PLL (PP), oxidized dextran (OD), and SeNPs. The injectability and self‐healing characteristics of OD‐PP@SeNPs hydrogels are mainly attributed to dynamic Schiff base bonds, Phenylboronate ester bonds, and hydrogen bonds. The introduction of SeNPs as initiators and nano‐fillers into the hydrogel resulted in extra cross‐linking of the polymer network through hydrogen bonding, and the SeNPs improved the mechanical properties of the hydrogel via reconfiguring various bonds. The presence of Schiff base bonds and Phenylboronate ester bonds leads to OD‐PP@SeNPs hydrogel with dual ROS/pH response, allowing for intelligently controlled release of SeNPs. Meanwhile, Schiff base bonds and Phenylboronate ester bonds consume ROS and adjust pH during covalent bond cleavage. SeNPs, Schiff base bonds, and Phenylboronate ester bonds exert synergistic antioxidant and anti‐inflammatory effects. We prepared OD‐PP@SeNPs hydrogel for the first time and sought to exploit the properties of the material itself to reshape the inflammatory microenvironment, relieve inflammation, and attempt to illustrate the mechanism of ROS clearance. Briefly, we first evaluated in detail the physicochemical properties, drug release characteristics, biocompatibility, in vitro antimicrobial activity, and in vivo retention of OD‐PP@SeNPs hydrogels. Second, the effects and mechanisms of hydrogel remodeling of the inflammatory microenvironment were comprehensively explored at the cellular level. Finally, the therapeutic effects of OD‐PP@SeNPs on *S. aureus* infected rats with skin injuries and rheumatoid arthritis (RA) rats were assessed, respectively. This study not only lays a function for tri‐cross‐linked hydrogel loaded with SeNPs on anti‐inflammatory mechanisms but also for the clinical applications.

## 2 Results and Discussion

### 2.1 Preparation and Characterization of SeNPs

**Figure 1:**
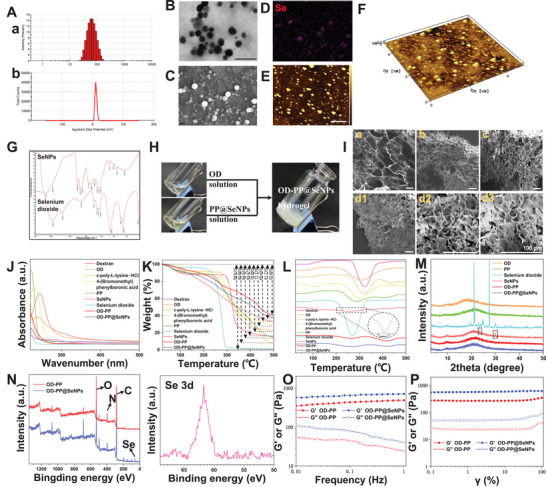
Characterizing data on the SeNPs and hydrogels. A) DLS data for SeNPs. (a) Particle size and (b) Zeta potentials. The Surface morphology of SeNPs. B) TEM, Scale bar = 200 nm. C) SEM, Scale bar = 200 nm, and E,F) AFM, Scale bar = 1 µm. D) EDS image of Se on SeNPs. G) The FT‑IR spectra of SeNPs and selenium dioxide. H) Photos of the OD‐PP@SeNPs hydrogel formation by mixing OD solution and PP@SeNPs solution. I) SEM micrographs of hydrogels (I). (a) OD‐PP, (b) OD‐PP@SeNPs‐500 µm, (c) OD‐PP@SeNPs‐1000 µm, and (d1, d2, d3) OD‐PP@SeNPs‐2000 µm. Scale bar = 100 µm. Evaluation of SeNPs and polymers characteristics by J) UV/vis, K) TGA, L) DSC, and M) XRD. N) XPS spectrum of the OD‐PP hydrogel and OD‐PP@SeNPs hydrogel. O) Frequency sweep tests of the hydrogels. P) Strain sweep tests of the hydrogels.

SeNPs were prepared by the template method. The particle size of SeNPs was 57.44 ± 2.34 nm (**Figure** **1**A‐a), Zeta potential was 10.7 ± 2.95 mV (Figure 1‐b), and PDI was 0.206 ± 0.051. SeNPs had uniform particle size and positive charge. Transition metal nanosystems with a size limitation of 45–115 nm were demonstrated to have satisfactory anti−inflammatory effects.^[^ ^46^ ^]^ The surface morphology of SeNPs was observed by TEM (Figure 1B), SEM (Figure 1C), and AFM (Figure 1E,F), and SeNPs were found to be regular spherical and sphere−like with a uniform particle size of ≈50 nm, similar to the DLS results. The sharp lattice fringes revealed the crystal structure of the particles. Energy−dispersive X‐ray spectroscopy (EDX) was used to investigate the composition of SeNPs and the presence of elemental Se. The EDS results indicated the presence of Se in the nanoparticles (Figure 1D). SeNPs also contained numerous C, O, and N elements on the surfaces (Figure S1, Supporting Information). It is presumed that chitosan (CS) covers the Se surface. SeNPs and CS exhibited a core‐shell growth mode with SeNPs encapsulated by CS, preventing Se aggregation. The content of Se measured by ICP‐MS was 3.17 ± 0.29% (W/W). Figure 1G is the FT‐IR results of SeNPs and selenium dioxide. The peak at 1411.6 cm^−1^ was attributed to the symmetric stretching vibration of COO─. The peak at 645.9 cm^−1^ was attributed to the bending vibration of Se─O, and the peaks at 1115.3 and 1114.2 cm^−1^ correspond to the stretching vibration of SeO_2_, confirming the presence of Se.^[^ ^47^ ^]^ The absorption peaks at 2936.2 and 1350.6 cm^−1^ represented the C─H stretching and vibrations. The peak at 1048.9 cm^−1^ represented the C─O stretching and C─C stretching backbone vibrations. At 931 cm^−1^, the A‐type absorption peak of the furan ring appears, which is the characteristic absorption peak of carbohydrate molecule vibration, and there was an α−pyranose absorption peak at 761.2 cm^−1^. The peaks at 1680.8 and 3365.9 cm^−1^ were shifted from 1638.3 and 2926.7 cm^−1^ for SeNPs alone. These peaks were assigned to the bonds Se─O and Se─C, respectively, indicating that SeO_2_ was completely reduced to zero‐valent selenium. The Se─O bond enhanced the stability of SeNPs. The above FT‐IR results suggested the formation of SeNPs. Due to the effect of hydroxyl groups in CS, a more stable system can be formed with SeO_2_. Therefore, the CS modified nanoparticles were more uniform and monodisperse. Figure 1J shows that the SeNPs have an absorption maximum of 264 nm, that attributed to the surface plasmon response of SeNPs. The results were similar to those reported.^[^ ^48^, ^49^ ^]^ The above results indicated that SeNPs were successfully prepared.

### 2.2 Synthesis and Characterization of the Polymers

The oxidation of dextran by periodate ions is an aqueous reaction without catalysis, and the purified product can be obtained by simple dialysis. The lyophilized OD appears as a white, odorless, loose structure. The FT‐IR spectrum of OD showed characteristic peaks of aldehydes and ketones at 1731.2 cm^−1^ (Figure S3, Supporting Information), indicating that the hydroxyl groups on the dextran are oxidized to aldehyde groups.^[^ ^50^ ^]^ In addition, in addition to the ─C═O stretching vibration peak on the aldehyde group mentioned above, other absorption peaks of OD are consistent with those of dextran, indicating that only a part of the hydroxyl groups on the dextran are changed into aldehyde groups. The peaks at 3.4 and 3.7 ppm in the ^1^H‐NMR spectrum were the hydroxyl proton peaks which are not entirely reacted in the dextran. The peaks ≈5.0–5.7 ppm were the aldehyde proton peaks on OD (Figure S4, Supporting Information). The GPC results showed that the molecular weight of OD is 67 753 Da, which was lower than 83 168 Da of dextran (Figure S5, Supporting Information). These results showed hydroxyls on the molecular chain are oxidized to the aldehyde group through an oxidation reaction, and the oxidation degree was ≈31.49 ± 1.81%. The theoretical oxidation degree was 50%. The theoretical oxidation degree was higher than the actual, probably because the NaIO_4_ content failed to react fully with the dextran, reducing the oxidation efficiency. Figure S6 (Supporting Information) shows the FT‐IR of PP (Figure S6, Supporting Information). The new peak at 1650.8 cm^−1^ is a stretching vibration of the C═O in the amide bond. The vibrational peaks occurring at 1580.8 and 1557.4 cm^−1^ are tensile vibrations of C═C in the benzene ring and B─O in the boronic acid moiety. The ─CH bending peak appears at 1364.8 cm^−1^. The peak at 1401.1 cm^−1^ represents C─B. In addition, the C─H stretching vibration of phenylborates is mainly observed at 725.0 cm^−1^. The peak at 7.3–7.7 ppm in the ^1^H‐NMR spectrum is the benzene ring (Figure S7, Supporting Information). PP has a similar absorption peak (295 nm) with 4‐ (Bromomethyl) phenylboronic acid (Figure 1J). Maldi‐Top results showed that the molecular weight of PP (4746.02 Da) was higher than that of PLL (4116.80 Da) (Figure S8, Supporting Information). The above results demonstrated the successful grafting of phenylboronic acid onto the PLL side chain.

### 2.3 Fabrication of OD‐PP@SeNPs Hydrogel

An adhesive injectable hydrogel with antibacterial, anti‐inflammatory, anti‐oxidation, and ROS scavenging properties was prepared and applied to treat inflammatory diseases in this study. The preparation process is exhibited in Figure 1H. The cross sectional morphology of the hydrogel was observed by SEM, and all of the hydrogels had cross‐linked networks, exhibiting a 3D porous mesh structure with uniform distribution (Figure 1I). The porous hydrogel help deliver gases and nutrients, reshaping the inflammatory microenvironment. In contrast, the addition of SeNPs makes the pore size smaller. Within the experimental range, the more SeNPs contained in the hydrogels, the smaller the pore size (from 209.11 ± 14.07 to 27.37 ± 3.78 µm). The addition of SeNPs changes the distance between OD‐PP chains and increases the cross‐linking sites, leading to a more dense and complex network backbone. The EDS results showed that SeNPs were uniformly distributed in the hydrogels, indicating good dispersion of SeNPs in the hydrogel systems (Figure S9, Supporting Information). OD‐PP@SeNPs‐2000 µm was used for all subsequent studies and was noted as OD‐PP@SeNPs.

FT‐IR is a vital tool for surveying functional groups and their interaction. The absorption peak of 3428.3 cm^−1^ represents the stretching vibration of ─OH (Figure S10, Supporting Information). The peaks at 3365.9 and 1632.2 cm^−1^ are attributed to ─OH and C═O. The intramolecular hydrogen bonding of OD‐PP@SeNPs appears to be weakened compared to OD‐PP. This is because some of the ─OH and ─COOH hydrogen bonds in the polymer were occupied with coordinating with SeNPs, which increased intermolecular hydrogen bonding. The characteristic absorption peak of the imine group appears near 1674 cm^−1^, indicating the formation of Schiff base bonds. 1085 cm^−1^ is the characteristic peak of the Phenylboronate ester bonds. It showed the presence of Schiff base bonds and Phenylboronate ester bonds in the hydrogel. The physical and chemical changes in the polymers as a function of temperature were evaluated by the STA analysis. Figure 1K shows the weight loss percentage of materials. The weight loss of materials as a function of temperature was evaluated via TGA. The samples were heated at 25–500 °C, and the TGA curve for each sample was plotted. Compared with selenium dioxide, the thermogravimetric loss of SeNPs was significantly reduced. A second thermogravimetric loss was observed at ≈260 °C, which may be due to the decomposition, oxidation, and reduction reactions of polysaccharides occurring on the surface of SeNPs. The thermogravimetric loss of PP was between that of PLL and 4‐bromomethylphenylboronic acid, indicating that the thermal stability of PP was higher than that of PLL. The addition of SeNPs in the OD‐PP system obviously changed the thermal degradation process: A reduction in the start point of mass loss temperature. It loses moisture at 78 to 153 °C and decomposes slowly at 256 to 500 °C. These results indicated that the crystallinity of the nanoparticles decreases as the melting point changes. This also showed that the thermal stability of OD‐PP@SeNPs hydrogel is better than that of the OD‐PP hydrogel. The DSC analysis indicates the characteristics of polymer materials, including the degree of crystallization, intra‐ and intermolecular interaction, mixability, and homogeneity. The results of DSC showed that the materials (dextran, OD, PLL, 4‐bromomethylphenylboronic acid, and PP) all had heat absorption peaks in the range of 280 to 330 °C, indicating a continuous moisture loss (Figure 1L). Compared with OD‐PP hydrogel, the positions of all peaks in OD‐PP@SeNPs hydrogel were red‐shifted, indicating that the addition of SeNPs increased the thermal stability of the system. Moreover, the characteristic peak intensity of SeNPs in the DSC spectrum of OD‐PP@SeNPs hydrogel was low, and the degree of crystallization region decreased. X‐ray diffraction patterns are a valid tool used to resolve the degree of the crystallinity of materials and structures. The diffraction peak at 2θ = 20.46 ° was due to the hydrated crystalline phase of PP and OD (Figure 1M). SeNPs showed characteristic peaks at 2θ = 24.19 and 31.14°, this confirmed the existence of SeNPs crystallites. SeNPs addition can cause structural variation in the hydrogel. OD‐PP@SeNPs showed a decrease in the intensity of peaks without changing, demonstrating a reduction in the crystallinity region. This also suggests SeNPs are highly dispersed in the hydrogel system and cause an increase in the ratio of amorphous due to differences in the cross‐link density. This showed that the addition of SeNPs causes interactions between SeNPs and the polymeric matrix.^[^ ^51^ ^]^ This is consistent with the DSC results. The Se 3d (61.7 eV) peak in the XPS spectrum of OD‐PP@SeNPs hydrogel indicated that SeNPs were successfully added to the hydrogel system (Figure 1N). C, O, and N characteristic peaks were also detected in the XPS spectrum, indicating that C, O, and N elements were the primary elements constituting the hydrogel (Figure S11, Supporting Information).

### 2.4 Properties of OD‐PP@SeNPs Hydrogel

It is an arduous task to incorporate a variety of functional characteristics into a single hydrogel system to satisfy the sophisticated requirements of IDs. Hydrogels must have good stability and certain mechanical properties to resist external pressure and ensure their integrity for clinical applications.^[^ ^52^ ^]^ The dynamic mechanical properties of hydrogels were evaluated by oscillation frequency experiments. The storage modulus (G′) reveals the elastic properties of the hydrogels and the loss modulus (G″) reveals the viscous properties of the hydrogels.^[^ ^53^ ^]^ In the range of 0.01 to 1 Hz, G′ was higher than G″ for all groups without any cross trace (Figure 1O), indicating stable hydrogel formation and excellent elasticity. The oscillation of G″ may be related to the breakage and recombination of dynamic Schiff base bonds, Phenylboronate ester bonds, and hydrogen bonds in the gel network.^[^ ^54^ ^]^ The strain scan results showed (Figure 1P) that the difference between G′ and G″ gradually becomes smaller in the range of 0.1 to 100%, indicating that the mechanical strength of OD‐PP hydrogel is slightly weaker. The G' of the OD‐PP@SeNPs hydrogel group was higher than that of the OD‐PP hydrogel group, reflecting that the addition of SeNPs increased the mechanical properties of the hydrogel. The hydrogen bonding in hydrogels contributes to the toughness of hydrogels. The addition of SeNPs also altered the mechanical properties of the hydrogels. The tensile stress‐strain curves of OD‐PP and OD‐PP@SeNPs hydrogels showed that the tensile stress and elongation at the break of the hydrogels increased significantly with the addition of SeNPs (Figure S12, Supporting Information). The tensile strain of the OD‐PP@SeNPs hydrogel increased from 83.33 ± 3.06% to 142.33 ± 4.51%. This is attributed to the fact that Schiff base bonds, Phenylboronate ester bonds, and hydrogen bonds help maintain the stability of the gel skeleton during stretching.

**Figure 2:**
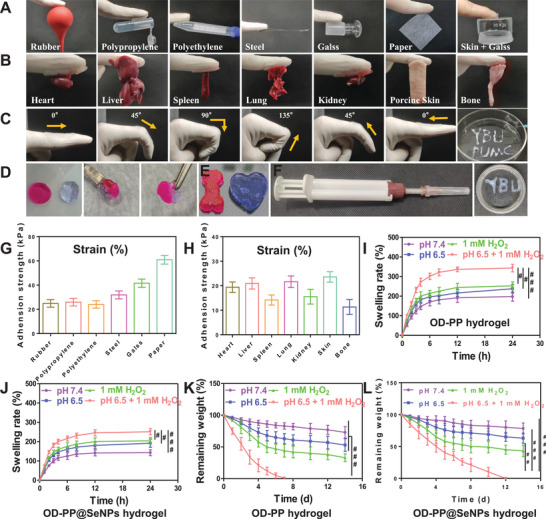
Properties of OD‐PP@SeNPs Hydrogel. A) Photographs of the hydrogels adhesion to different substrates. B) Digital images demonstrating the macroscopic adhesion of the hydrogels to various tissues from a rat. C) Softness of OD‐PP@SeNPs hydrogel. D) The self‐healing procedure between two separate OD‐PP@SeNPs hydrogels pieces. OD‐PP@SeNPs hydrogels were stained with Nile red and crystal violet stains. E) Hydrogel injected into various shapes (moldability). F) Injectability of OD‐PP@SeNPs hydrogels and a picture of the crossing needle‐equipped double‐barrel syringe. G,H) Adhesion strength of OD‐PP@SeNPs hydrogels to different substrates (*n* = 3). Swelling rate of I) OD‐PP hydrogel and J) OD‐PP@SeNPs hydrogel (*n* = 3). Remaining weight of K) OD‐PP hydrogel and L) OD‐PP@SeNPs hydrogel (*n* = 3).

The hydrogel lacking adhesion will detach and cause inflammation. The excellent adhesion ability of the hydrogel ensures that the hydrogel is in close contact with the tissue, keeping the wound moist and preventing bacterial invasion.^[^ ^52^ ^]^ In addition, the hydrogel needs to maintain its adhesion in distorted and moist environments. OD‐PP@SeNPs hydrogel can tightly adhere to various substrate surfaces (**Figure** **2**A), and the adhesion strength is greater than 20 kPa (Figure 2G). For adhesion evaluation in humid environment, the major tissues and organs of rats were used as model materials. Impressively, the OD‐PP@SeNPs hydrogel produced stable adhesion to the tissue, demonstrating excellent adhesion strength (Figure 2B,H). The hydrogel could adhere to the finger and resist the bending force of the finger without breaking, indicating that the hydrogel had good skin adhesion (Figure 2C). After removing the hydrogel, there were no allergic reactions, such as redness and inflammation. And there is no residual hydrogel. The Schiff base bonds, Phenylboronate ester bonds, and hydrogen bonds endow the hydrogel with strong adhesion properties, as the abundant hydroxyl, amino, carboxyl groups, and electrostatic forces can bind to tissues. The above results preliminarily indicated that OD‐PP@SeNPs hydrogel has excellent adhesive ability and effectively overcomes the shortcomings of traditional hydrogel.

The high mobility of the human body tends to deform the fragile hydrogel, leading to the formation of cracks. The self‐healing behavior of broken hydrogels is a huge advantage for hydrogel applications, extending hydrogel life and sustainability and reducing dosing frequency. The self‐healing behavior of the OD‐PP@SeNPs hydrogel was displayed in Figure 2D. Two round‐shaped gels were physically cleaved into four halves and then instantly brought into contact. Hydrogels prepared based on dynamic bond cross‐linking usually have excellent self‐healing properties. The OD‐PP@SeNPs hydrogels are self‐healing via Schiff base bonds, Phenylboronate ester bonds, and hydrogen bonds. This ability could be recovered under mild conditions without the demand for any external stimulus. This provides the hydrogel with bring more potent bonding force and self‐healing ability. Injectability is ideal in biomedical applications for the topical administration of therapeutic hydrogels. The good injectability of the hydrogel facilitates the filling of irregularly shaped wounds and lesion cavities. Figure 2F illustrated the injectability of the OD‐PP@SeNPs hydrogel using a crossing needle‐equipped double‐barrel syringe. The hydrogel also demonstrated desirable processibility to shape the final architecture and automatic and seamless filling of irregularities (Figure 2E). The OD‐PP@SeNPs hydrogel could instantly recover into the initial shape after compression (Video S1, Supporting Information).

The equilibrium swelling rate and degradation properties are crucial for applying hydrogel, which will influence the absorption of tissue exudate and drug release.^[^ ^55^ ^]^ The swelling is mainly determined by the cross‐linking density, the water solubility of the polymer, and the morphological characteristics of the hydrogel. The swelling rates of all hydrogels increased sharply at the initial stage (Figure 2I,J). The swelling rates of OD‐PP and OD‐PP@SeNPs were 271.33 ± 20.03% (W/W) and 199.17 ± 12.25% at pH 6.5+1 mm H_2_O_2_ for 4 h, indicating their good absorption capability. Then both began to level off after 8 h, and finally were 343.33 ± 19.22% and 251.33 ± 16.04%, respectively. The swelling rates of both hydrogels at pH 6.5+1 mm H_2_O_2_ were the highest. The hydrophilic properties of OD and PP (containing ─OH and ─NH_2_) allow the hydrogel to retain large amounts of water in its network, enabling a favorable moist environment to be maintained at the application site. The OD‐PP@SeNPs hydrogel kept the tissue moist and absorbed exudate to prevent exudate buildup and sepsis, which was efficient for tissue repair. Based on the results, OD‐PP and OD‐PP@SeNPs exhibited excellent swelling properties, which could accelerate the absorption of excess fluid from hyperpermeable wounds. Generally speaking, excessive swelling will eventually break the hydrogel network and could result in elevated compression on neighboring tissues, this is not optimal for hydrogels. The addition of SeNPs reduced the swellability because SeNPs acted as cross‐linking agents, occupied some hydrophilic cross‐linking sites, and reduced the pore size. Thus, OD‐PP@SeNPs hydrogel exhibited proper swelling capacity, which gave the hydrogel the ability to absorb inflammatory tissue fluids and helped to alleviate oxidative stress. The reasonable degradation rate of biomaterials is an essential indicator for biomedical applications.^[^ ^56^ ^]^ Furthermore, after 14 d of submersion in pH 7.4 PBS, the weight reduction of the hydrogels was less than 21.67 ± 8.51% (Figure 2K), indicating that the degradation rate of these hydrogels was prolonged. The mass loss rate was accelerated at lower pH and high ROS. In 1 mm H_2_O_2_+pH 6.5 PBS, the OD‐PP hydrogels degraded to ≈100% of their initial weight at 7 d while demonstrating significant responsiveness. The addition of SeNPs retarded the degradation of the hydrogel (Figure 2L). This feature was also confirmed by the physical pictures of in vitro degradation (Figure S13, Supporting Information). It was completely degraded by 12 d, which is desirable for in vivo applications. The pH of normal synovial fluid ranges between 7.4–7.8, dropping to 6.5–7.2 in arthritic joints. Schiff base bonds and Phenylboronate ester bonds are pH‐responsive and theoretically susceptible to decomposition in inflammatory microenvironments with lower pH. The degradation and solubilization properties of hydrogels facilitate drug release from the gel network.

The in vitro drug release experiments help provide critical data regarding the performance of a dosage form under in vivo conditions.^[^ ^57^ ^]^ Based on the Schiff base bonds and Phenylboronate ester bonds, the OD‐PP and OD‐PP@SeNPs hydrogels exhibited responsive behavior under different pH and redox conditions. In this work, the pH/ROS dual‐responsive manner of the OD‐PP@SeNPs was examined under various conditions. Porous hydrogels have the function of storing and delivering a variety of drugs. In order to investigate the release properties of the hydrogel, we also prepared the hydrogel loaded with BSA‐FITC, which were labeled as OD‐PP@BSA‐FITC hydrogel and OD‐PP@SeNPs@BSA‐FITC hydrogel, respectively. Compared to OD‐PP@BSA‐FITC hydrogel, OD‐PP@SeNPs@BSA‐FITC hydrogel released BSA‐FITC at a lower rate than OD‐PP@BSA‐FITC in different environments. The cumulative OD‐PP@BSA‐FITC hydrogel release rate was 80.37 ± 8.56% in 1 mm H_2_O_2_+pH 6.5 PBS on 4 d (Figure S14A, Supporting Information). The cumulative release of OD‐PP@SeNPs@BSA‐FITC hydrogel was 50.07 ± 4.10% (Figure S14B, Supporting Information). The high cross‐linking density in the OD‐PP@SeNPs@BSA‐FITC hydrogel reduced the release speed of the drug to achieve the effect of sustained release. This is consistent with previous reports.^[^ ^58^ ^]^ The OD‐PP@SeNPs can also release SeNPs slowly and sustainably. At 8 d, in pH 6.5 PBS+1 mm H_2_O_2_, 32.06 ± 3.15% SeNPs were released (Figure S14C, Supporting Information). Most importantly, after being treated with low pH and high ROS simultaneously (pH 6.5 PBS+1 mm H_2_O_2_), the hydrogels would rapidly disintegrate and change into a concentrated solution, leading to the exposure of BSA‐FITC or SeNPs. The release results indicated that the hydrogel network was broken due to hydrolysis of the Schiff base bonds and Phenylboronate ester bonds under acidic and oxidative conditions. The OD‐PP@SeNPs hydrogel could not only rapidly release PP and OD with antibacterial and antioxidant properties but also sustainably release SeNPs, which have anti‐apoptosis activity. The Schiff base bonds and Phenylborate ester bonds in hydrogels also regulate the pH and ROS of the solution when hydrolyzed. Therefore, OD‐PP@SeNPs hydrogel has good application potential for treating inflammatory diseases. The release kinetics model studied the release characteristics of the hydrogel. The diffusional exponent (n) depended on the release mechanism and the shape of the drug delivery device: 0.45 < n < 0.89 indicates a non‐Fickian diffusion and erosion release mechanism where a combination of diffusion and polymer relaxation controlled release.^[^ ^59^ ^]^ The swelling and relaxation of polymer chains and the invasion of the matrix polymer were involved during the release process (Table S1, Supporting Information).

### 2.5 In Vitro Antioxidant Evaluation

Bioactive effects are also essential for therapeutic hydrogels. The inflammatory tissue usually loses the balance of oxidative and antioxidant systems resulting in oxidative stress and cell damage, which could promote the over generation of various radicals.^[^ ^60^ ^]^ The inactivation of RNA, DNA, protein, and lipid by radicals is irreparable and constant. H_2_O_2_, O_2_^−^•, and • OH are typical oxygen free radicals. The capability of hydrogel to scavenge radicals O_2_^−^• and • OH was compared with ascorbic acid. Ascorbic acid is an acidic polyhydroxy compound and is the most versatile antioxidant known, which is extremely reductive.^[^ ^61^ ^]^ The enediol in its molecular structure can be oxidized to a ketone group which scavenges free radicals.^[^ ^61^ ^]^

The DPPH method is widely used for the quantitative analysis of free radical scavenging ability, which has the advantages of good stability, high sensitivity, and simple operation. DPPH ethanol solution appears purple with a characteristic absorption peak at 517 nm, which can be used to monitor its reduction. After the reaction between SeNPs and DPPH, a decreased absorption peak was found (Figure S15, Supporting Information), and the DPPH solution exhibited marked decolorization. The DPPH scavenging ability of SeNPs demonstrated concentration‐dependent properties. As the content increased from 10 to 1000 µm, the DPPH scavenging ratio increased from 1.84% to 97.65% for SeNPs. When the DPPH solution was handled with OD‐PP@SeNPs hydrogel, complete color fading occurred, and there was no absorbance peak at 517 nm, revealing a better DPPH scavenging capability. The ABTS method has the advantages of simplicity, rapidity, and a strong correlation with the biological activity of antioxidants. ABTS can be oxidized into ABTS•+, and the solution occurs bright blue‐green with a characteristic absorption peak at 413 nm (Figure S16, Supporting Information). The absorbance peak dropped substantially upon SeNPs treatment, with evident fading. Especially, the peak disappeared after the addition of OD‐PP@SeNPs, and complete fading was noted, suggesting better ABTS scavenging capability. Hydroxyl radicals are the most potent oxygen radicals in the body and can react non‐specifically with nucleic acids, proteins, or lipids to cause damage to tissue cells. The catechol group in the hydrogel is considered to be able to scavenge • OH free radicals. As highlighted in Figure S17 (Supporting Information), the dependence of the scavenging ratios on the SeNPs concentration indicates that the scavenging ability increased with the SeNPs concentration. OD‐PP@SeNPs exhibited a higher scavenging ability than SeNPs due to the combined actions of SeNPs and OD‐PP hydrogel. These results illustrated that OD‐PP@SeNPs hydrogels based on Schiff base bonds and Phenylboronate ester bonds had excellent antioxidant capacity.

In summary, SeNPs and OD‐PP@SeNPs hydrogels could scavenge ROS as brilliant antioxidant agents to maintain the balance of the antioxidant defense system. This could be ascribed to the combined effects of SeNPs and OD‐PP hydrogels (Schiff base bonds and Phenylboronate ester bonds).

### 2.6 In Vitro Antibacterial Studies

Tissue infection is an excellent challenge in the clinical setting. Due to the long‐term inflammatory response, more exudate is secreted, which easily induces tissue infection. Bacterial metabolism and infection can generate unique microenvironments with pH differences. Bacterial fermentation produces lactic acid, which can acidify its surrounding pH as low as 3.5.^[^ ^62^ ^]^ Hence, aseptic conditions are essential for parenteral hydrogels to avoid contamination. At the same time, an ideal hydrogel should have antibacterial properties, which help reduce the number of bacteria in the tissue, control inflammation, and accelerate recovery. It has been reported that excess Se exhibits potent antibacterial activity.^[^ ^63^ ^]^ Thus, *E. coli* (early infection) and *S. aureus* (late infection) were used to evaluate the in vitro antibacterial performance of OD‐PP@SeNPs hydrogel and to explore the OD‐PP@SeNPs hydrogel potential antibacterial mechanism. The evaluation methods used were colony formation, OD value, bacterial precipitation, disc diffusion, and live & dead bacteria staining.

The antibacterial efficiency of hydrogels was evaluated through spread plate method.^[^ ^64^, ^65^, ^66^ ^]^ As expected, the majority of bacteria were inactivated after contact with materials (SeNPs, OD‐PP hydrogel, and OD‐PP@SeNPs hydrogel) for 12 h (Figure S18A, Supporting Information), demonstrating excellent antimicrobial activity against two kinds of bacteria. Compared with the Model groups, all groups treated with OD‐PP@SeNPs hydrogel showed almost no bacterial clones on the agar plates. The excellent antibacterial properties of OD‐PP@SeNPs hydrogel were preliminarily proved. The bacteriostatic properties of OD‐PP@SeNPs hydrogel were further characterized by OD value and bacterial precipitation detection. The bacteria treated with OD‐PP@SeNPs hydrogels aggregated and precipitated (Figure S18B, Supporting Information), indicating the destruction of the bacteria. The OD at 600 nm also decreased significantly (Figure S18C, Supporting Information). Antibacterial activity after 12 h of incubation was assessed by measuring the diameter of the clear zone of inhibition. Clear antibacterial zones were generated around SeNPs, OD‐PP hydrogel, and OD‐PP@SeNPs hydrogel (Figure S18D, Supporting Information). OD‐PP@SeNPs hydrogel inhibited the growth of two bacteria (*E. coli* and *S. aureus*) were 12.27 ± 1.03 and 13.25 ± 1.24 mm, respectively. Both SeNPs and OD‐PP hydrogel had little antibacterial zones. The integrity of the bacterial cell membrane was examined by SYTO9 (LIVE dye)/propidium iodide (PI) double fluorescent staining and visualized by confocal laser scanning microscopy (CLSM), where green means live bacteria and red means dead bacteria. The Model group showed strong green fluorescence. In contrast, both SeNPs and OD‐PP@SeNPs hydrogels showed extensive red fluorescence (Figure S18E, Supporting Information). Some bacteria were stained both green and red at the same time and presented an orange color. These co‐staining results demonstrated that the bacteria were injured but not completely killed. The antimicrobial effect of SeNPs and OD‐PP hydrogel alone was not very satisfactory. The combination of SeNPs and OD‐PP into OD‐PP@SeNPs hydrogel significantly enhanced the antibacterial performance. In conclusion, these data demonstrate the efficient antibacterial properties of OD‐PP@SeNPs hydrogel.

### 2.7 Cell Researches

A perfect hydrogel should have superior cytocompatibility regardless of direct contact and the degradation products. The biocompatibility of hydrogels was evaluated with Mouse Leukemia Cells of Monocyte Macrophage (RAW264.7), Human Umbilical Vein Endothelial Cells (HUVECs), and Mouse Fibroblast Cells (L929).

**Figure 3:**
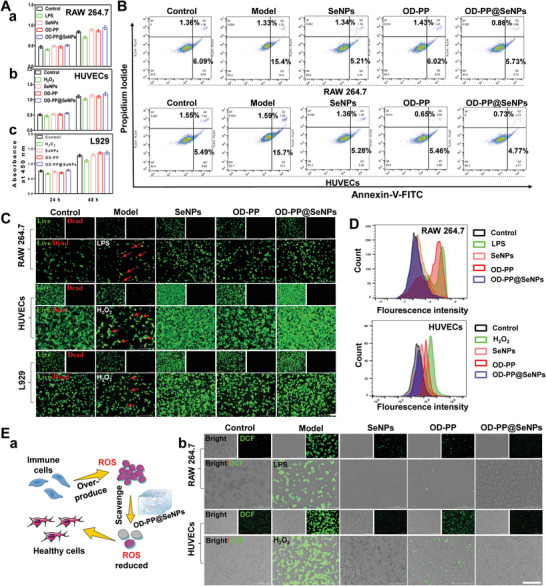
Biocompatibility and ROS clearance of OD‐PP@SeNPs hydrogel. A) Cell viability of (a) RAW264.7, (b) HUVECs, and (c) L929 with the different preparations at various time (*n* = 6). B) Flow cytometry analysis of RAW264.7 and HUVECs cells treated with different preparations for 24 h. The cells were stained with Annexin V‐FITC and Propidium Iodide for analysis (*n* = 3). C) Live & dead staining of RAW264.7, HUVECs, and L929 after incubation with the different preparations for 24 h. Scale bar = 200 µm. D) ROS scavenging of different preparations by flow cytometry (*n* = 3). E) DCFH‐DA staining. (a) Schematic diagram of the ROS scavenging mechanism. (b) CLSM. Scale bar = 100 µm.

The optimal induction concentrations of LPS and H_2_O_2_ were screened by cell activity and oxidative indicators (NO and ROS), respectively. And the final concentration of LPS was 1 µg mL^−1^ (Figure S19, Supporting Information), and the concentration of H_2_O_2_ was 200 µm (Figure S20, Supporting Information). After being induced by LPS or H_2_O_2_, the cell viability was decreased and more NO or ROS was produced, showing that the cell model was successfully constructed. Then, the effects of different concentrations of SeNPs on the activity of normal cells and inflammatory cells were evaluated by CCK 8 and live & dead cell staining. The results showed that the cell viability was greater than 80% in the concentration range of 10–1000 µm (Figures S21–S23, Supporting Information). Among them, SeNPs can significantly promote cell proliferation at low concentrations. Next, the cytotoxicity of hydrogel to normal and inflammatory cells was tested against, and CCK‐8 (**Figure** **3**A; Figure S24, Supporting Information) and flow cytometry (Figure 3B) results showed satisfactory biocompatibility at both 24 and 48 h. The results of live & dead cell staining (Figure 3C) exhibited that all the cells in the OD‐PP@SeNPs hydrogel treatment were mainly in a viable state (green), and only negligible dead cells (red) appeared. During the whole culture process, the cells tended to adhere closely and steadily formed cell colonies, indicating the good growth status of the three cells and the excellent biocompatibility of the hydrogels. Together, these results demonstrated the outstanding biocompatibility of OD‐PP@SeNPs hydrogels.

As a factor of inflammation, intracellular ROS can trigger and amplify inflammatory responses and even cause oxidative stress damage. Intracellular ROS levels in inducer‐stimulated RAW264.7 and HUVECs were assessed by 2′,7′‐dichlorofluorescein diacetate (DCFH‐DA) staining, respectively.^[^ ^67^ ^]^ First, the concentration of SeNPs was screened, and a dose‐dependent ROS scavenging capacity was found in the concentration range of 10–500 µm (Figure S25, Supporting Information). When the concentration reached 750 µm, the clearance ability decreased. Figure 3E a is a schematic diagram of the ROS scavenging mechanism of the hydrogel. A strong green fluorescence signal was observed in the Model group (Figure 3E‐b), indicating high ROS levels. More importantly, the OD‐PP@SeNPs hydrogel group showed no fluorescence intensity, indicating that OD‐PP@SeNPs hydrogel has the best antioxidant property. The synergistic effect of OD‐PP hydrogel and SeNPs can scavenge ROS to the maximum extent. The flow cytometry experiments obtained similar results (Figure 3D; Figure S26, Supporting Information). OD‐PP@SeNPs hydrogel exhibited excellent cytoprotective and antioxidant properties.

Oxidative stress is a primary marker for understanding IDs pathogenesis and progression. Then, the intracellular superoxide dismutase (SOD) activity, Glutathione peroxidase (GPx) level, and malondialdehyde (MDA) content assay were performed to address the intracellular anti­oxidative properties of the OD‐PP@SeNPs hydrogel. SOD is a vital antioxidant metalloproteinase in cells, which can resist oxidative damage caused by external stimuli. Its activity reflects the severity of oxidative damage to cells.^[^ ^68^ ^]^ The SOD in the LPS group was significantly decreased, demonstrating the enzyme oxidizing system of macrophages was damaged under inflammation (Figure S27A, Supporting Information). The SOD activities of the administered group (SeNPs, OD‐PP hydrogel, and OD‐PP@SeNPs hydrogel) were significantly restored, especially the OD‐PP@SeNPs hydrogel group. Se is a critical metal co­factor for the catalytic activity of SOD and is likely to reduce oxidative stress by enlarging the activity of this enzyme.^[^ ^69^ ^]^ This implies that OD‐PP@SeNPs hydrogel has the ability to protect intracellular SOD activity. GPx is an important peroxidase in cells, which can reduce peroxides to non‐toxic hydroxyl compounds and protect cell functions from damage. Se is an essential component of GPx. The activity of the enzyme is determined by the activity of selenocysteine, which can be used to measure the Se level of the body. GPx levels were reduced in the LPS group due to the inflammatory response (Figure S27B, Supporting Information). However, stable GPx levels were maintained in the administered groups. Surprisingly, the OD‐PP@SeNPs hydrogel group had comparatively higher GPX levels. This suggested that OD‐PP@SeNPs hydrogel effectively reduced oxidative stress and protected GPx from depletion. MDA is a toxic product formed by oxidative stress in cells. Its level represents the severity of oxidative damage in tissues. A significant increase in MDA levels was detected in the LPS group. The MDA levels in the administered group were similar to those of normal cells. Among them, OD‐PP@SeNPs hydrogels were not significantly different from the control group (Figure S27C, Supporting Information). This also indicated that OD‐PP@SeNPs hydrogel effectively reduced oxidative damage. Based on these results, it is proved that OD‐PP@SeNPs hydrogel could enhance the resisting oxidative stress capability of macrophages (RAW264.7) by suppressing oxidants and stimulating autophagy.

**Figure 4:**
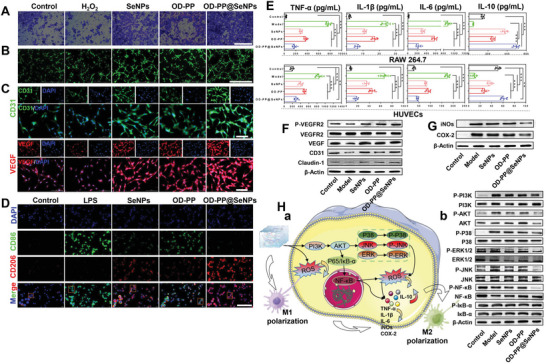
Angiogenesis performance in vitro and anti‐inflammatory mechanism of OD‐PP@SeNPs hydrogel. A) The ability of hydrogel to migrate vertically. Scale bar = 1000 µm. B) Tube formation capacity of HUVECs after incubation with the different preparations for 6 h. Scale bar = 1000 µm. C) Immunofluorescent staining of CD31 (green), VEGF (red), and DAPI (blue) after incubation with the different preparations for 24 h. Scale bar = 200 µm. D) Macrophage polarization by CD86 (M1, green), CD206 (M2, red), and DAPI (blue). Scale bar = 200 µm. E) The level of cytokines (TNF‐α, IL‐6, IL‐1β, and IL‐10) with RAW264.7 and HUVECs cells after incubation with the different preparations for 24 h (n = 6). F) Western blots of P‐VEGFR2, VEGFR2, VEGF, CD31, and Claudin‐1 by HUVECs incubated in different preparations for 24 h. J) Western blots of iNOs and COX‐2 by RAW264.7 incubated in different preparations for 24 h. K) Anti‐inflammatory mechanism of OD‐PP@SeNPs hydrogel. (a) A mechanical diagram of OD‐PP@SeNPs hydrogel remodeling of the inflammatory microenvironment. (b) Western blots of PI3K/AKT/NF‐κB and MAPK signaling pathways by RAW264.7 incubated in different preparations for 24 h.

The inflammatory state affects the nature of cell migration. The scratch wound assay was carried out to investigate the ability of the OD‐PP@SeNPs hydrogel to promote in vitro cell migration. Compared with the Model group, all administered groups could narrow the scratch gap of HUVECs cells, and OD‐PP@SeNPs hydrogel had the strongest healing ability (Figure S28, Supporting Information). Because excessive ROS production at the inflammation site causes oxidative stress and leads to cytotoxicity by damaging DNA and enzymes, OD‐PP@SeNPs hydrogel with antioxidant capacity can accelerate inflammation recovery by scavenging ROS. The effect of different preparations on the vertical migration of HUVECs cells was examined by Transwell (**Figure** **4**A). This indicated that OD‐PP@SeNPs hydrogel promoted the vertical migration of HUVECs cells. At concentrations ≤100 µm, SeNPs did not exert a cell migration‐promoting effect (data not shown). Endothelial cell vascularization is a prominent process in angiogenesis. The in vitro tube formation assay was implemented to inspect the angiogenic ability of SeNPs and OD‐PP@SeNPs hydrogel. SeNPs can promote tube formation only at concentrations ≥100 µm (Figure S29, Supporting Information). OD‐PP@SeNPs hydrogel improved the ability of HUVECs cells to generate blood vessels in vitro (Figure 4B).

CD31 is an endothelial cell marker expressed in early vascular development to identify newly formed vessels.^[^ ^70^ ^]^ Meanwhile, VEGF is considered a vascular permeability factor that can contribute to the proliferation and migration of HUVECs cells.^[^ ^71^, ^72^ ^]^ VEGF is also a protein that is essential for supporting tissue growth. Therefore, we observed the effect of hydrogel on the expression levels of CD31 and VEGF. The results of immunofluorescence showed that the OD‐PP@SeNPs hydrogel accelerated angiogenesis by promoting the proliferation and migration of HUVECs cells, as well as increasing the expression levels of CD31 and VEGF (Figure 4C,H). Western blotting (WB) experiments found that OD‐PP@SeNPs hydrogel promoted the expression of CD31 and VEGF proteins (Figure 4F; Figure S30, Supporting Information), which was consistent with the results of Figure 4C. In addition, OD‐PP@SeNPs hydrogel increased the ratio of phosphorylated Vascular Endothelial Growth Factor Receptor 2 (P‐VEGFR2) to Vascular Endothelial Growth Factor Receptor 2 (VEGFR2). Furthermore, the hydrogel also increased Claudin‐1 protein levels. Therefore, OD‐PP@SeNPs hydrogel could accelerate the maturation and proliferation of HUVECs by promoting the secretion of CD31, VEGF, and Claudin‐1, exerting the effect of promoting cell angiogenesis. The above results indicated that OD‐PP@SeNPs hydrogel at this dose is the best candidate to promote cell adhesion, spreading, and proliferation. This phenomenon can be attributed to the synergistic effect of OD‐PP hydrogel and SeNPs. The addition of the hydrogel provides more adhesion sites for the cells, enabling cell adhesion, spreading, and proliferation. SeNPs released from the hydrogel can also promote cell viability and proliferation, owing to its role as an essential dietary micronutrient at a low level.

Clinical evidences disclose that many macrophages are significantly increased in IDs patients with the inflammatory tissue.^[^ ^73^ ^]^ Macrophages have many phenotypes, and the balance of different phenotypes plays an essential role in the inflammatory cascade, mainly divided into M0 (resting state), M1, and M2 phenotypes.^[^ ^74^ ^]^ The M1 phenotype secretes inflammatory cytokines, accelerating oxidative processes and inhibiting tissue repair. And the M2 phenotype secretes anti‐inflammatory cytokines, which reduce the release of inflammatory factors and promote tissue repair and antioxidant activity.^[^ ^75^ ^]^ An imbalance in M1/M2 balance leads to a chronic inflammatory response. The cells not only secret proinflammatory cytokines such as TNF‐α, IL‐6, and IL‐1β but also accelerate the degradation of the cartilage extracellular matrix. TNF‐α is the predominant proinflammatory cytokine that initiates the inflammatory cascade and is also a central proinflammatory cytokine in the pathogenesis of IDs. Macrophages mainly produce IL‐1β and play an important role in bone erosion, synovial hypertrophy, leukocyte infiltration, and cartilage destruction. It exerts proinflammatory effects principally by regulating the expression of various macrophages, immunomodulatory molecules, cell adhesion molecules, and inflammatory mediators. TNF‐α and IL‐1β have synergistic effects, and both interact with each other to mediate joint damage and inflammatory responses. IL‐10 is a crucial anti‐inflammatory cytokine, which can effectively inhibit the effector T cell response. To study the anti‐inflammatory effects of the OD‐PP@SeNPs hydrogel, we investigated protein expression in RAW264.7 and HUVECs. ELISA assays were used to detect the levels of TNF‐α, IL‐1β, IL‐6, and IL‐10. OD‐PP@SeNPs hydrogel significantly decreased TNF‐α, IL‐1β, and IL‐6 levels and increased IL‐10 (Figure 4E). The severity of inflammation was strongly correlated with high levels of iNOs proteins and increased NO concentrations. COX‐2 is an isoenzyme and mutagenic enzyme with shallow activity in cells of normal joints. When these cells are stimulated by inflammation, the COX‐2 expression level in inflammatory cells increases, causing increased content of vasodilatory prostaglandins at inflammatory sites and resulting in an aggravated inflammatory response and tissue damage. OD‐PP@SeNPs hydrogel treatment of LPS‐induced RAW264.7 cells revealed a significant decrease in both iNOs and COX‐2 protein levels (Figure 4G). The above results suggested that OD‐PP@SeNPs hydrogel alleviated the inflammatory state of cells and regulated the inflammatory microenvironment.

The polarization of macrophages toward an M2 phenotype from M1 is essential for inflammation relief.^[^ ^76^ ^]^ The anti­inflammatory ability of OD‐PP@SeNPs hydrogel is related to macrophage phenotypes regulation and inflammatory microenvironment modulation. In order to verify this hypothesis, the immunofluorescence experiments of RAW264.7 cells were carried out. The addition of LPS effortlessly induced the M1 phenotype (CD86) polarization of the RAW264.7 macrophages, whereas the addition of OD‐PP@SeNPs hydrogel remarkably induced the M2 phenotype polarization (CD206) in the presence of LPS, demonstrating the shift toward M2 macrophage polarization (Figure 4D). This suggests that OD‐PP@SeNPs hydrogel exerts anti‐inflammatory efficacy by promoting macrophage transition from M1 to M2 phenotype. We further studied the anti‐inflammatory mechanism of the hydrogel. The results of WB found that the hydrogel could reduce the phosphorylation level of PI3K‐AKT. PI3K/AKT pathway is a signaling pathway related to cell proliferation, differentiation, migration, protein synthesis, and inhibition of inflammation. Extracellular signal‐regulated kinases (ERK1/2), c‐Jun N‐terminal kinases (JNK), and p38 kinases, which belong to the MAPK family, are readily activated in the presence of intracellular ROS.^[^ ^77^ ^]^ JNK, P38, and ERK are thought to play important roles in oxidative stress and apoptosis. NF‐κB pathway, including NF‐κB and IκB‐α, is widely involved in response to external stimuli. Some studies have shown that NF‐κB plays a key role in the polarization of M1‐type cells.^[^ ^78^ ^]^ Figure 4H‐a represents a mechanical diagram of hydrogel remodeling of the inflammatory microenvironment. Macrophages were induced by LPS, activating NF‐κB and MAPK pathways. The phosphorylation of ERK1/2, JNK, and P38 was significantly reduced after treatment with the preparation (Figure 4H‐b), demonstrating that the hydrogel can inhibit the activation of the MAPK pathway induced by LPS and reduce ROS levels. Treatment with hydrogel significantly reduced P‐PI3K and P‐AKT levels in the pathway. At the same time, the hydrogel also reduced the expression of related proteins in the NF‐κB pathway and inhibited NF‐κB signal transduction. Figure S31 (Supporting Information) presents protein expression quantification results. The above results indicated that the OD‐PP@SeNPs hydrogel reduces the expression of TNF‐α, IL‐1β, IL‐6, iNOs, and COX‐2, increases IL‐10 secretion, promotes the transformation of macrophages from M1 to M2, remodels the inflammatory microenvironment, and exerts anti‐inflammatory effects via PI3K/AKT/NF‐κB and MAPK pathway.

### 2.8 In Vivo Retention of Hydrogel

All animal experiments were approved by the Laboratory Animal Ethics Committee in the Institute of Materia Medica and Peking Union Medical College (0 0008582 and 0 0008361). All procedures followed ethical standards during the experiment.

Frequent administration may result in secondary tissue damage and secondary infections. Therefore, it is crucial to construct a hydrogel with long‐term mechanical stability and reduce the frequency of administration. LPS is usually used to induce inflammatory responses and ROS production in vivo.^[^ ^79^, ^80^ ^]^ New Indocyanine Green (IR820) was added to the OD‐PP@SeNPs hydrogel, and the hydrogel was detected in the abdominal subcutaneous and joint cavity retention experiments by the in vivo imaging system. Compared with in vitro degradation, the hydrogel showed a faster degradation rate in vivo, which was attributed to the body temperature of 37 °C and the complex in vivo environment. In LPS (‐) and Healthy group, it is manifest that the OD‐PP@SeNPs hydrogel could be retained locally for at least 14 d (Figures S32A and S33A, Supporting Information), and it displayed persistent maintenance and sustained release. The results exhibited that OD‐PP@SeNPs hydrogel, which can be used for drug retention and controlled release, is a potential local controlled release drug delivery system. In LPS (+) and Inflammatory group, the retention time of hydrogel in vivo was significantly shortened, and the inflammatory environment accelerated the metabolism of hydrogel, reflecting the inflammatory responsiveness of hydrogel. Figure S32C (Supporting Information) is a photo of the hydrogel injected subcutaneously on 1 d.

In the abdominal subcutaneous retention experiment, neither detectable signs of damage nor inflammatory lesions after inspection 14 d, and the body weight of these rats consistently increased (Figures S32B and S33B, Supporting Information). No inflammatory cells were discovered, and collagen fibers and fibroblasts appeared regular (Figure S34, Supporting Information). Furthermore, no apparent inflammatory lesions or tissue damage were seen in the organs after the intra‐articular injection of the hydrogel. By hematology analysis, no significant differences were observed between all groups in Lymphocyte percentage (LY), Neutrophil count (NEUT), Red blood cells (RBC), Hemoglobin (HGB), Platelet count (PLT), and White blood cell count (WBC) (Figure S35, Supporting Information). In addition, all these results were within the reference range of normal rat blood parameters (Table S2, Supporting Information), indicating no dehydration, hemorrhage, infection, or immune system disorders in any group. The above results showed that the OD‐PP@SeNPs hydrogel does not cause an inflammatory response and possesses high biosafety.

### 2.9 Treatment of IDs In Vivo

The excellent anti‐inflammatory ability of hydrogel encourages us to explore its therapeutic effects in vivo further. We investigated the therapeutic effects of OD‐PP@SeNPs hydrogel on two typical IDs, for infected wound healing and RA. Wound healing is considered an immensely complicated and regulated process. The phases of skin wound healing include the inflammatory phase, the repair phase, and the maturation phase.^[^ ^81^ ^]^ After wound hemostasis, inflammatory cells migrate to the wound to facilitate the inflammatory phase, which is characterized by a continuous infiltration of associated cells such as macrophages that may produce ROS. The high levels of ROS lead to cellular and wound damage. Therefore, clearing excess ROS, reducing oxidative stress and inflammatory responses during wound healing, shortening the inflammatory phase, and shifting the wound enter the cell proliferation stage as soon as possible, wound healing can be effectively accelerated. RA is a chronic systemic autoimmune disease. Oxidative stress, due to the disruption of the balance between ROS generation and the antioxidant defense system, performs an essential part in pathophysiology of RA. The foreign antioxidants can play a key role in the treatment of RA by removing excessive production of free radicals and reducing bone erosion of the joints. Therefore, treatments that control the development of inflammation may inhibit IDs progression, such as modulating the inflammatory microenvironment.

**Figure 5:**
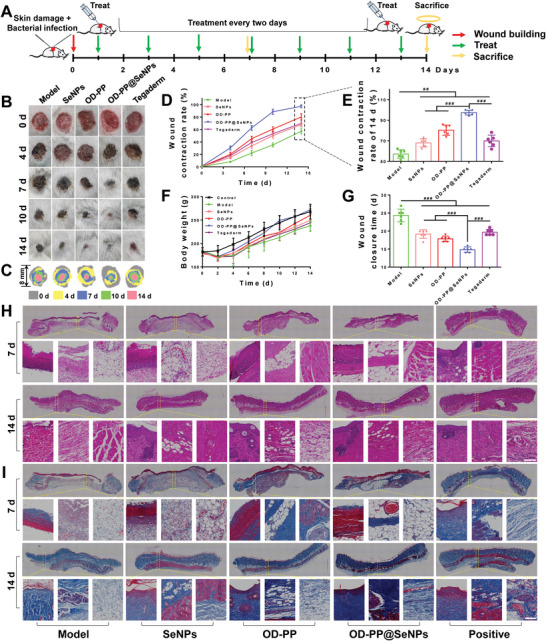
In vivo evaluation of OD‐PP@SeNPs hydrogel in the infected rats wound model. A) A schematic illustration of the process and timeline of infected rats wound model. B) Macroscopic photographs of the wounds healing process with different treatments on 0, 3, 7, 10, and 14 d. C) A physical map of the wound healing process. D) Wound contraction rate during 14 d after treatment with the different preparations (*n* ≥ 6). E) Wound contraction rate of 14 d (*n* = 6). F) Body weight during 14 d after treatment with different preparations (*n* = 6). G) Wound closure time after treatment with the different preparations (*n* = 6). Histological evaluation of wound in different groups. H) H&E and I) Masson trichrome stained sections of wound tissue with different treatments at 14 d. Scale bar = 100 µm.

The *S. aureus* infected rats with skin defect model was performed to evaluate the actual effect of OD‐PP@SeNPs hydrogel in promoting infected wound healing. **Figure** **5**A is a schematic illustration of the process and timeline of an infected rat wound model. The commercially available Tegaderm ^3M^ wound dressing serves as the positive group. At 14 d, the wounds in the model group (untreated) failed to heal themselves (Figure 5B), similar to previous reports.^[^ ^82^ ^]^ The wounds in the OD‐PP@SeNPs group healed utterly. The rats in other groups still had different degrees of injury and scab. The dynamic process of wound repair was remodeled with a contour map (Figure 5C). The healing rate was significantly faster in the OD‐PP@SeNPs hydrogel group than in the other groups (Figure 5D). Comparing the relative wound areas, at 14 d, the wounds in the OD‐PP@SeNPs hydrogel group entirely healed without scarring, and the wound contraction rate reached 97.35 ± 2.32% (Figure 5E). During the experiment, the body weight of rats with wound infection decreased first and then increased. Body weight of the OD‐PP@SeNPs hydrogel group was ultimately similar to that of the Control group (Figure 5F). The wound closure time was significantly shorter in the OD‐PP@SeNPs hydrogel group, averaging 14.83 ± 0.75 d (Figure 5G). The visible results provided that the OD‐PP@SeNPs hydrogel can keep wounds clean and promote wound healing. The healing effect of rat infected injuries was even grander than that given by the clinically used Tegaderm ^3M^ with the addition of an antimicrobial agent.

Base on the phases of skin wound healing, it is essential to shorten the inflammatory period and promote wound repair sufficiently. H&E staining and Masson staining were used to observe the different stages of regenerated skin to assess the anti‐inflammatory and pro‐collagen deposition characteristics of the OD‐PP@SeNPs hydrogel. H&E staining results were consistent with wound contraction rates (Figure 5H). At 7 d, the Model group was free of epidermal formation and collagen deposition. At 14 d, there was still an apparent inflammatory reaction in the Model group, with amounts of inflammatory cell infiltration. Wounds treated with OD‐PP@SeNPs hydrogel shrank faster than the other groups, especially early in healing. At 14 d, the OD‐PP@SeNPs hydrogel group showed an intact epidermal layer and fewer inflammatory cells on the wound with more fibroblasts, neovascularization and hair follicles, and significant granulation tissue with better connective tissue and epidermal structure was observed. The proliferation of fibroblasts, keratinocytes, new capillaries, endothelial cells, and inflammatory cells produces granulation tissue. Additionally, the controlled and sustained release of SeNPs from the OD‐PP@SeNPs hydrogel was profitable for increased collagen deposition, accelerated wound healing, boosted the formation of granulation tissue and epithelialization, and wound closure. The thickness of the epidermis is also an important indicator to assess the wound healing process,^[^ ^83^ ^]^ and the OD‐PP@SeNPs hydrogel group had the best epidermal thickness, which was similar to that of the surrounding healthy skin. This indicates that the keratin‐forming cells in this group began to degenerate, implying that the wound was nearing completion. This result was consistent with the physical map of the wounds (Figure 5C). The level of collagen expression can reflect the degree of wound healing to a certain extent.^[^ ^84^ ^]^ Hence, Masson trichrome staining was carried out to study newly formed collagen (Figure 5I). On 14 d, residual scabs were still noticeable in the Model group, showing a delayed healing process of the infected wound. The red‐stained necrotic tissue with little blue‐stained collagen appeared in the Model and Tegaderm group. By contrast, the OD‐PP@SeNPs hydrogel group showed a distinct red epidermal layer and an extreme collagen volume. The highest density of collagen and the best organized fibrous structure were found in the OD‐PP@SeNPs hydrogel group at any time. The results indicated that OD‐PP@SeNPs hydrogel could extremely heighten the collagen deposition during the wound healing process. Besides, excessive vascularisation during wound healing can also produce side effects such as wound fibrosis and scar formation. The OD‐PP@SeNPs hydrogel group held substantially higher blood vessel densities at the late stages of wound healing. However, no significant scarring was formed.

**Figure 6:**
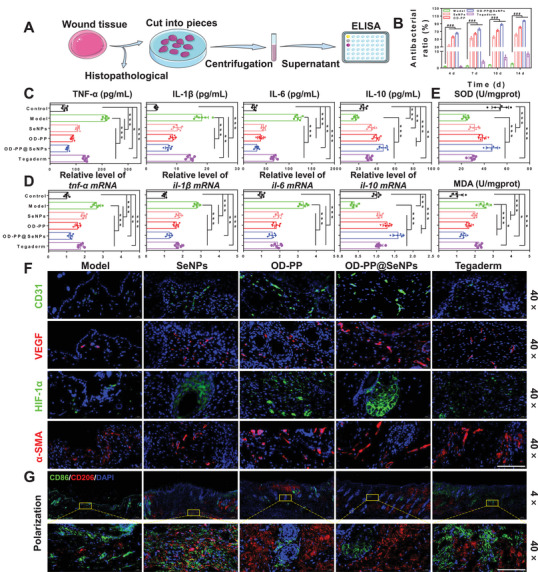
In vivo antibacterial, anti‐inflammatory, and vascularization capacity of the OD‐PP@SeNPs hydrogel. A) A schematic diagram of the wound tissue detection process. B) Antibacterial effect of different preparations in vivo (*n* = 3). C) Expression levels of TNF‐α, IL‐1β, IL‐6, and IL‐10 after treatment with the different preparations at wound tissue (*n* = 6). D) Gene expression levels of *tnf‐α, il‐1β, il‐6*, and *il‐10* after treatment with the different preparations at wound tissue (*n* = 6). E) MDA and SOD levels at the wound site (*n* = 6). F) Immunofluorescence staining images of CD31 (green), VEGF (red), HIF‐1α (green), α‐SMA (red), and DAPI (blue) at the wound tissue with different treatments at 14 d. Scale bar = 200 µm. G) Immunofluorescence staining of macrophage phenotype at the wound tissue with different treatments at 14 d. CD86 (green), CD206 (red), and DAPI (blue). Scale bar = 200 µm.

**Figure** **6A** is a schematic diagram of the wound tissue detection process. Bacterial infection is both the cause and one of the potential complications of chronic wounds. At the same time, a sustained inflammatory response can further hinder the healing process from the inflammatory phase to the proliferative one. Skin tissue surrounding the wound site was excised, and the wound was homogenized for in vivo bacterial inhibition assays. The results showed that OD‐PP@SeNPs hydrogel had an outstanding bacterial inhibition effect in vivo, with a bacteriostatic rate of 97.65 ± 2.15% (Figure 6B). The long‐lasting inflammation may lead to further tissue damage. A dihydroethidium probe was used to examine the ROS scavenging capacity in vivo to prove the ROS content in the wound tissue. Bright red fluorescence signals and high fluorescence intensity were found in the Model group (Figure S36, Supporting Information). In contrast, the fluorescence intensity in the SeNPs and OD‐PP hydrogel group reduced noticeably. Almost no fluorescence signal could be found in the OD‐PP@SeNPs hydrogel group. The results showed that OD‐PP@SeNPs hydrogel could scavenge ROS to lessen oxidative stress. Elisa assay assessed the expression levels of TNF‐α, IL‐1β, IL‐6, and IL‐10 as the critical inflammatory mediator during wound healing. The highest TNF‐α, IL‐1β, IL‐6, and the lowest IL‐10 expression levels were observed in the Model group (Figure 6C), implying a severe inflammatory response. At 14 d, minimal expression of TNF‐α, IL‐1β, and IL‐6 was observed in the OD‐PP@SeNPs hydrogel group. In contrast, a significant amount of IL‐10 was detected. This result was generally consistent with the results of RT‐PCR (Figure 6D). These findings suggested that OD‐PP@SeNPs hydrogel effectively attenuated the inflammatory response at tissue sites, consistent with the in vitro results. To further assess the effects of oxidative stress, MDA and SOD levels at the wound site were measured, as they typically reflect the degree of oxidative damage to the tissue. Compared with the Model group, the MDA level was significantly decreased, and the SOD content was significantly increased in the OD‐PP@SeNPs hydrogel group (Figure 6E), which may be attributed to the antioxidant and anti‐inflammatory effects of SeNPs and OD‐PP hydrogel, regulating the inflammatory microenvironment and protecting cells and proteins from oxidative damage. Neovascularization supplies nutrition to cells in the new wound tissue, preserves continued tissue growth, and is vital for wound contraction, re‐epithelialization, and wound healing. The immunostaining of CD31, VEGF, hypoxia inducible factor‐1 (HIF‐1α), and α‐Smooth muscle actin (α‐SMA) was observed to estimate the existence of newly generated vessels in the wound site on 14 d (Figure 6F). The granulation tissue from the treatment groups had more CD31, VEGF, and HIF‐1α than the Model group on 14 d, with OD‐PP@SeNPs hydrogel exhibiting the highest level. This suggested that the hydrogel promoted wound angiogenesis through the HIF‐1α‐VEGF pathway, scavenged • OH radicals, and alleviated the hypoxic state at the wound site. Impressively, OD‐PP@SeNPs hydrogel expressed less α‐SMA than the OD‐PP group. Excessive α‐SMA can be deposited on the skin surface and form scars.

The mechanism of OD‐PP@SeNPs hydrogel in remodeling the wound inflammatory microenvironment was further explored. Macrophages are crucial players in the local immune responses to tissue damage. Combined with the in vitro results of OD‐PP@SeNPs hydrogel promoting the transformation of M1 to M2 macrophages, we further researched whether the decreased inflammation and enhanced healing were related to the polarization status of macrophages in wounds. Double immunofluorescence staining was carried out to establish the number of M2 macrophages (CD206) and M1 macrophages (CD86) expression. The Model group showed the most intense green fluorescence, which implies a massive infiltration of M1‐type macrophages and a highly inflammatory state of the wound (Figure 6G). More M2 macrophages and low M1 macrophages were observed in the OD‐PP@SeNPs hydrogel group, with a higher ratio between M2 and M1, displaying a reduced inflammatory response and better anti‐inflammatory characteristics. This is due to the combination of SeNPs and OD‐PP hydrogel. The progressive tilt of M1 macrophages to M2 is the defining event in the transition from the inflammatory phase to the proliferative phase.

Thus, these two factors illustrate the prominent wound healing properties of OD‐PP@SeNPs hydrogel. On the one hand, antibacterial. SeNPs and PP inhibited the growth of bacteria at the wound site, effectively reduced the inflammation caused by bacteria, and accelerated the wound healing of *S. aureus* infection. On the other hand, it reshapes the inflammatory microenvironment. OD‐PP@SeNPs hydrogel continuously released SeNPs. The presence of SeNPs, Schiff base bonds, and Phenylboronate ester bonds induced the polarization of M1 macrophages to M2 type, reduced the level of inflammatory cytokines, decreased the level of ROS in the inflammatory site, shortened the inflammatory phase, reshaped the inflammatory microenvironment, and made the wound enter the cell proliferation stage as soon as possible. In particular, SeNPs promote endothelial cell proliferation and angiogenesis, accelerating wound healing and avoiding excessive vascularization without leaving visible scars.

**Figure 7:**
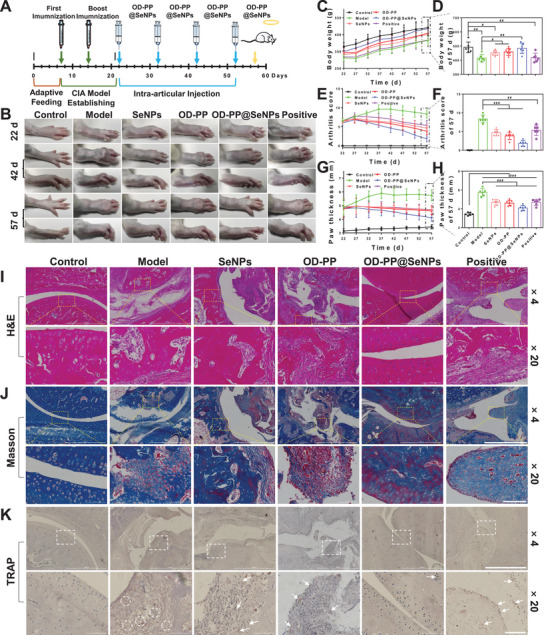
In vivo therapeutic effects of OD‐PP@SeNPs hydrogel treatment in RA rats. A) Overall experimental timeline for OD‐PP@SeNPs hydrogel treatment in RA rats model with collagen‐induced arthritis. B) Representative hind paw images of the sample‐treated RA rats after the different preparations treatment. C) Body weight of rats during different treatments (*n* = 6). D) Body weight of RA rats at 57 d. (*n* = 6). E) Time‐dependent average arthritis score of RA rats determined at each evaluation time point. F) Average arthritis score of RA rats at 57 d. (*n* = 6). G) Paw thickness changes over time in RA model after the sample treatment. (*n* = 6). H) Paw thickness of RA rats at 57 d. (*n* = 6). Histological assays with I) H&E, J) Masson, and K) TRAP staining of the joint tissue after the sample treatment. × 4, Scale bar = 1000 µm. × 20, Scale bar = 100 µm.

The collagen‐induced arthritis (CIA) model is one of the animal models of RA currently used in preclinical studies of drugs. It is highly similar to human RA regarding clinical features, pathological changes, and immune response.^[^ ^85^ ^]^ Therefore, a CIA rat model was established as a pathological model of RA to assess the role of OD‐PP@SeNPs hydrogel in RA. Methotrexate (MTX), the gold standard for RA, was used as a Positive group.^[^ ^86^ ^]^ **Figure** **7**A shows the flow chart of animal experiments for RA. The paw thickness and arthritis index of the rats were evaluated every 5 d to reflect the severity of arthritis and the anti‐arthritic activity of the hydrogel. Observing changes in body weight, arthritis score, and paw thickness in disease or drug‐treated conditions is critical to knowing the pathological state or progression of the animal condition.^[^ ^87^ ^]^ The fully developed arthritis was confirmed in the Model group after observing noticeable swelling, tenderness, deformity, and ankyloses (Figure 7B). As shown in Figure 7E–H, treated rats demonstrated noteworthy improvement in the clinical index and reduction of paw thickness. Body weight was recovered in the treatment group (SeNPs, OD‐PP, and OD‐PP@SeNPs) (Figure 7C,D). Specifically, the best results were observed in the OD‐PP@SeNPs hydrogel group. In this case, paw thickness was reduced compared with the Model group at 57 d. The Model group rats could not walk normally in the balance beam experiment. In contrast, rats of the OD‐PP@SeNPs hydrogel group can slowly cross the balance beam (Video S2, Supporting Information). The time for rats to cross the balance beam was recorded (Figure S37, Supporting Information) and was significantly shorter in the OD‐PP@SeNPs hydrogel group compared to the other groups. The results illustrated the excellent therapeutic effect of OD‐PP@SeNPs hydrogel.

The pathological state of the RA condition is related to continual synovitis, progressive cartilage, and bone destruction.^[^ ^88^ ^]^ The progression or recovery of the RA rats following OD‐PP@SeNPs hydrogel administration was confirmed by evaluating the status of bone, cartilage, and synovium from 57 d. Synovial hyperplasia and joint structure of CIA rats were evaluated by H&E staining (Figure 7I). The Control group rats had intact synovial tissue cells, no inflammatory cell infiltration, a smooth and undamaged articular cartilage surface, intact joint structure, and no cartilage destruction. In the Model group, there were noticeable pathological changes in the joints, such as synovial tissue hyperplasia in the joint cavity, vascular expansion in the subsynovial tissue, fibrosis, and adhesion of some synovial tissues, destruction of joint soft tissues, bone erosion, pannus formation, accompanied by many inflammatory cell infiltration and cell arrangement disorder. The pathological sections of the Positive group rats showed that the articular surface was smooth, but the cell morphology was changed, and there were signs of fibrosis. OD‐PP@SeNPs have a better effect than free MTX in alleviating joint deformation and bone erosion. OD‐PP@SeNPs hydrogel can significantly improve pathological conditions and greatly reduce symptoms such as synovial hyperplasia, inflammatory cell infiltration, and cartilage invasion. Figure 7G is the result of Masson staining. Blue represents newly formed bone and red represents mature bone tissue. The joints of the Control group and OD‐PP@SeNPs hydrogel group showed regular blue areas, indicating normal morphology of a large amount of new bone. An increase in bone mass indicated increased bone synthesis and decreased bone breakdown. Due to the vital role of osteoclasts in bone catabolism, joint tissue sections were stained with tartrate resistant acid phosphatase (TRAP) to assess the number of osteoclasts. TRAP is a marker enzyme of osteoclasts and is usually used for identifying osteoclasts.^[^ ^89^ ^]^ The cytoplasm of osteoclasts stained by TRAP was wine red. Compared with the Model group, OD‐PP@SeNPs hydrogel can effectively inhibit the generation of osteoclasts (Figure 7K), thereby reducing the absorption of bone tissue and significantly alleviating the erosion and destruction of bone and joints.

**Figure 8:**
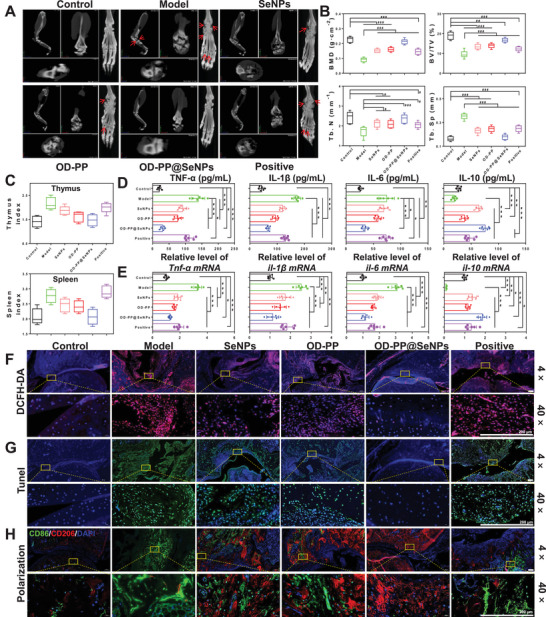
Evaluation of the therapeutic efficacy after OD‐PP@SeNPs hydrogel treatment. A) Representative paw X‐ray images of RA rats with detailed ankle images for each. The red arrows indicate bone destruction and damage. B) Quantitative results of bone status, including bone mineral density (BMD), bone volume‐tissue volume (BV/TV), trabecular number (Tb. N), and trabecular separation (Tb. Sp) (*n* = 3). C) Organ index of RA rats after different treatments at 57 d (*n* = 6). D) Expression levels of TNF‐α, IL‐1β, IL‐6, and IL‐10 after treatment with the different preparations in RA rats (*n* = 6). E) Gene expression levels of *tnf‐α, il‐1β, il‐6, and il‐10* after treatment with the different preparations in RA rats (*n* = 6). F) ROS staining of ankle joint in RA rats after treatment with the different preparations. ROS (red) and DAPI (blue). Scale bar = 200 µm. G) Tunel staining. Apoptotic cells (green) and DAPI (blue). Scale bar = 200 µm. H) Immunofluorescence staining of macrophage phenotype at ankle joint with different treatments at 57 d. CD86 (green) and CD206 (red). Scale bar = 200 µm.

RA is an autoimmune inflammatory disease, and its final pathological features are often cartilage damage and bone erosion.^[^ ^90^ ^]^ Therefore, X‐RAY was used to assess the effect of OD‐PP@SeNPs hydrogel on blocking bone damage and erosion by joint imaging. The Control group rats had smooth joint surfaces, intact morphology, no bone erosion, and regular spaces (**Figure** **8**A). The ankle joints of rats in the model group exhibited obvious bone erosion and structural damage with impaired integrity and significantly more joint space (red arrows). Bone damage and erosion were significantly reduced after treatment with OD‐PP@SeNPs hydrogel. The morphological parameters of ankle trabecular bone were quantitatively analyzed (Figure 8B). Compared with the Model group, the OD‐PP@SeNPs hydrogel group significantly increased bone mineral density (BMD), bone volume‐tissue volume (BV/TV), and trabecular number (Tb. N), reducing the trabecular separation (Tb. Sp), the bone mass in bone trabeculae increased. The results indicated that OD‐PP@SeNPs hydrogel has effective repair ability and efficacy against bone destruction caused by arthritis. Organ indices were calculated to study the effect of hydrogel on organ function in rats. Abnormal function of the thymus, a central immune organ, is one of the primary pathogenesis of RA. The thymus is the site of T‐cell differentiation and maturation, and when its function is abnormal, it affects T‐cell levels and the normal immune inflammatory state.^[^ ^91^ ^]^ In addition to the thymus, the spleen is a peripheral immune organ that plays an important role in regulating neuroendocrine immunity in the body. The level of thymus and spleen indices depends on the degree of lymphocyte proliferation and can reflect the strength of the immune function. Therefore, their relative weights are usually used as preliminary indicators to estimate the immune‐regulatory activity of tested substrates used for treatment. The thymus index and spleen index of the Model group were increased, indicating that the organs had congestion, edema, hypertrophy, and other symptoms. MTX treatment increased thymus and spleen indices, probably due to its adverse effects.^[^ ^92^ ^]^ There was no significant change in the OD‐PP@SeNPs hydrogel organ index compared to the Control group (Figure 8C). The results confirmed that immune regulation due to the treatment of SeNPs as hydrogel played an essential role in the anti‐arthritic activity of SeNPs. The imbalance between inflammatory and anti‐inflammatory cytokines is a major factor contributing to the initiation and progression of RA. Therefore, the cytokine levels as an index of therapeutic efficacy were measured in RA rats treated with different formulations. This study found that the contents of TNF‐α, IL‐1β, and IL‐6 in the Model group rats were significantly increased (Figure 8D), which once again proved that the abnormal expression of inflammatory indicators in the CIA rats was closely related to joint injury. Compared with the Model group, TNF‐α, IL‐1β, and IL‐6 were significantly decreased, and IL‐10 was significantly increased in the OD‐PP@SeNPs hydrogel group. The gene expression (Figure 8E) and immunohistochemical results (Figure S38, Supporting Information) of the inflammatory factors were consistent with the Elisa results. Further immunofluorescence studies of the joint showed that OD‐PP@SeNPs hydrogel reduced ROS levels at the joint site (Figure 8F), reduced the regular apoptosis rate (Figure 8G), and restored cell viability. Polarization of macrophages in the joint tissues was also evaluated by immunofluorescence staining. The Model group secreted large amounts of CD86 (green), indicating severe inflammation (Figure 8H). After treatment with OD‐PP@SeNPs hydrogel, CD86 was significantly reduced in the joint site and many CD206 (red) was secreted. The results showed that OD‐PP@SeNPs hydrogel could induce polarization of macrophages from M1 to M2 phenotype, remove dysfunctional and hyper‐proliferated macrophages from inflammatory sites, and significantly reduce inflammation.

The above results revealed that OD‐PP@SeNPs hydrogel could promote macrophage polarization, down‐regulate the levels of proinflammatory cytokines (TNF‐α, IL‐1β, and IL‐6), increase IL‐10 secretion, eliminate ROS in joints, and reshape the inflammatory microenvironment. Finally, the OD‐PP@SeNPs hydrogel inhibited synovial hyperplasia/inflammation, reduced osteoclast formation, improved cartilage and bone reconstruction, reduced CIA rat foot swelling, improved immune organ index, and achieved the effect of relieving RA.

### 2.10 In Vivo Toxicity

Hemocompatibility is a fundamental feature of biomaterials since they are in direct or indirect contact with blood. The hemolytic test was performed to evaluate the blood compatibility of the hydrogels. The supernatant of all formulations (OD‐PP@SeNPs hydrogel and SeNPs) with blood cells showed a yellowish to reddish (Figure S39, Supporting Information) and a small amount of red precipitate at the bottom of the tube, suggesting that OD‐PP@SeNPs hydrogel and SeNPs had no or slight hemolysis, and the hemolysis rate was less than 2%. Therefore, the OD‐PP@SeNPs hydrogel has excellent hemocompatibility. And the hemolysis ratio is below the permitted limit of 5% for biomaterials in the application concentration range.

The biocompatibility of the OD‐PP@SeNPs hydrogel was systematically assessed in vivo. H&E staining exhibited that all the preparation groups were safe for the rats because of the ignorable organ damage and normal cell morphology (Figures S40 and S41, Supporting Information). These results certified that the OD‐PP@SeNPs hydrogel is excellent biosafety. The body weight of rats in all groups did not fluctuate significantly during treatment, suggesting that the OD‐PP@SeNPs hydrogel has no systematic toxicity and did not affect the growth of rats. Blood biochemical analysis showed that the typical liver and kidney function indicators of rats in all groups are within normal ranges (Figures S42 and S43, Supporting Information). These results exhibited that the SeNPs released at the tissue site from hydrogel would not exert any systematic toxicity, demonstrating the extraordinary biocompatibility of the OD‐PP@SeNPs hydrogel in vivo, which holds great promise for treating IDs.

## 3 Conclusion

We prepared for the first time a dynamically responsive hydrogel that integrated multiple functions based on PBA grafted PLL, oxidized dextran, and SeNPs. The OD‐PP@SeNPs hydrogel has a triple‐network structure due to the role of SeNPs as ligands besides Schiff base bonds and Phenylboronate ester bonds, thus resulting in a tough composite hydrogel. The OD‐PP@SeNPs hydrogel has ideal mechanical properties, injectable, self‐healing, adhesion, good flexibility, swelling capacity, biodegradability, stimuli‐responsive active substance release performance, and excellent biocompatibility. The OD‐PP@SeNPs hydrogel with ROS scavenging and pH‐regulating ability protects cells from oxidative stress and induces macrophages into M2 polarization to reduce inflammatory cytokines through PI3K/AKT/NF‐κB and MAPK pathways, exerts anti‐inflammatory effects and reshapes the inflammatory microenvironment. All these features make tri‐cross‐linked dynamically responsive hydrogels as promising biomimetic materials for antibacterial, therapeutic IDs, and other biomedical applications. In the future, we will explore the pharmacological mechanism of OD‐PP@SeNPs hydrogel in modulating the inflammatory microenvironment in vivo and broaden their applications.

## Conflict of Interest

The authors declare no conflict of interest.

## Author Contributions

Z.G., W.H., and S.W. designed this study. S.W. carried out experiments on synthesis, in vitro cell experiments and animal experiments. Y.L., Q.S., B.Z., C.L., L.G., H.W., and L.C. assisted with the animal experiments and Software. The major contribution of M.J., W.H., and J.G. was the teaching method. S.W. analyzed the data and wrote this manuscript. Z.G. revised this manuscript.

## Supporting information

Supporting Information

Supplemental Video 1

Supplemental Video 2

## Data Availability

The data that support the findings of this study are available from the corresponding author upon reasonable request.
