# Supplementary material for: Triple Cross‐linked Dynamic Responsive Hydrogel Loaded with Selenium Nanoparticles for Modulating the Inflammatory Microenvironment via PI3K/Akt/NF‐κB and MAPK Signaling Pathways
Source: Adv Sci (Weinh). 2023 Sep 22;10(31):2303167. doi: 10.1002/advs.202303167 (PMC10625091; doi:10.1002/advs.202303167)
Supplement: Supplementary file 1 — Supporting Information [file ADVS-10-2303167-s001.pdf]

ADVANCED SCIENCE
Open Access

# Supporting Information

for *Adv. Sci.*, DOI 10.1002/advs.202303167

Triple Cross-linked Dynamic Responsive Hydrogel Loaded with Selenium Nanoparticles for Modulating the Inflammatory Microenvironment via PI3K/Akt/NF-$\kappa$B and MAPK Signaling Pathways

*Shuangqing Wang, Yanhong Liu, Qianwen Sun, Bowen Zeng, Chao Liu, Liming Gong, Hao Wu, Liqing Chen, Mingji Jin, Jianpeng Guo, Zhonggao Gao* and Wei Huang**

# Triple Crosslinked Dynamic Responsive Hydrogel Loaded with Selenium Nanoparticles for Modulating the Inflammatory Microenvironment *via* PI3K/Akt/NF-κB and MAPK Signaling Pathways

Shuangqing Wang [1, 2, 3], Yanhong Liu [1, 2], Qianwen Sun [1, 2], Bowen Zeng [1, 2], Chao Liu [1, 2], Liming Gong [1, 2], Hao Wu [1, 2, 3], Liqing Chen [1, 2], Mingji Jin [1, 2], Jianpeng Guo [3], Zhonggao Gao [1, 2, 3, *], Wei Huang [1, 2, *]

[1.] State Key Laboratory of Bioactive Substance and Function of Natural Medicines, Institute of Materia Medica, Chinese Academy of Medical Sciences and Peking Union Medical College, Beijing 100050, China

[2.] Beijing Key Laboratory of Drug Delivery Technology and Novel Formulations, Department of Pharmaceutics, Institute of Materia Medica, Chinese Academy of Medical Sciences and Peking Union Medical College, Beijing 100050, China

[3.] Key Laboratory of Natural Medicines of the Changbai Mountain, Ministry of Education, College of Pharmacy, Yanbian University, Yanji 133002, Jilin Province, China

[*.] Corresponding author: Zhonggao Gao (zggao@imm.ac.cn), and Wei Huang (huangwei@imm.ac.cn)

No potential conflicts of interest were disclosed.

**1. Table of Contents Text**

**2. Materials and Methods**

2.1 Materials

2.2 Preparation of SeNPs

2.3 Synthesis of Oxidized Dextran

2.4 Synthesis of Phenylboronic Acid Grafted Polylysine

2.5 Fabrication of OD-PP@SeNPs Hydrogel

2.6 Characterization of SeNPs and OD-PP@SeNPs Hydrogel

2.7 *In Vitro* Antioxidant Capacity of the Hydrogel

2.8 *In Vitro* Antibacterial Activity Assays

2.9 Cell Research

2.10 *In Vivo* Responsiveness and Retention

2.11 Wound Healing *in Vivo*

2.12 RA

2.13 *In Vivo* Toxicity

2.14 Statistical Analysis

**3. Discussion**

**Figure S1-S43**

**Table S1-S5**

**References**

## 2. Materials and methods

### 2.1 Materials

Immunization Grade Bovine Type II Collagen (20022) and Incomplete Freund's Adjuvant (7002) were purchased from Chondrex (Washington State, USA). Crystal Violet Staining Solution (C0121), Cell Counting Kit-8 (C0038), Calcein AM Cell Viability Assay Kit (C2013M), DCFH-DA (S0033M), 4% Paraformaldehyde Fix Solution (P0099), Immunostaining Permeabilization Buffer with Triton X-100 (P0096), QuickBlock™ Blocking Buffer for Immunol Staining (P0260), Phenylmethanesulfonyl fluoride (ST505), and Protease and phosphatase inhibitor cocktail for general use (P1046) were obtained from Shanghai Beyotime Biotech. Inc. (Shanghai, China). Triethylamine (12129), Ethylene glycol (12422), and Hydrogen peroxide (11641-2) were purchased from Beijing Tong Guang Fine Chemicals Company (Beijing, China). Dextran T-70 (D8260), DAPI solution(ready-to-use) (C0065)，LB Broth (powder, L1010), LB agar (powder, L1015), FBS, RIPA buffer (high) (R0010), BCA protein Assay kit (PC0020), and Matrigel Basement Membrane Matrix (356234) were purchased from Beijing Solarbio Science & Technology Co., Ltd (Beijing, China). Selenium dioxide (S817942), 2,2-Diphenyl-1-picrylhydrazyl (DPPH, D807297), 2,2'-Azino-bis (3-ethylbenzothiazoline-6-sulfonic acid) diammonium salt (ABTS, A800764), Ferrous sulfate (F903428), 4-(Bromomethyl)phenylboronic acid (B803441), Chitosan (C850347), and Sodium periodate (S817518) were acquire from Shanghai Macklin Biochemical Co., Ltd (Shanghai, China). ε-Poly-L-lysine·HCl (P192512) was obtain from Shanghai

Aladdin Biochemical Technology Co., Ltd (Shanghai, China). SYTO 9/PI Live/Dead Bacterial Double Stain Kit (MX4234-40T) was purchased from Shanghai Maokang Biotechnology Co., Ltd (Shanghai, China). Methotrexate (S18026) was obtained from Shanghai yuanye Bio-Technology Co., Ltd (Shanghai, China). L-Ascorbic acid (V900134) and Lipopolysaccharide (L2880) were purchased from Sigma (Merck KGaA, Darmstadt, Germany). Fetal bovine serum (26010074) was purchased from GIBCO LLC. (Grand Island, NY, USA). A Angiogenesis Assay Kit (ab204726), Goat Anti-Rabbit IgG H&L (Alexa Fluor® 488) (Alexa Fluor® 488 (ab150077), Goat Anti-Rabbit IgG H&L (Alexa Fluor® 647) (ab150079), Anti-CD86 Antibody [EPR21962] (ab239075), Anti-Mannose Receptor Antibody (ab64693), VEGF (ab32152), Anti-CD31 Antibody [RM1006] (ab281583), Anti-VEGF Receptor 2 Antibody (ab11939), Anti-VEGF Receptor 2 (phospho Y1214) Antibody (ab5475), Anti-Claudin 1 Antibody [EPRR18871] (ab211737), Anti-COX2 / Cyclooxygenase 2 Antibody [EPR12012] (ab179800), Anti-iNOS Antibody [EPR16635] (ab178945), Anti-PI 3 Kinase p85 alpha Antibody [EPR18702] (ab191606), Anti-PI 3 Kinase p85 alpha (phospho Y607) Antibody (ab182651), Anti-AKT1 (phospho S473) Antibody [EP2109Y] (ab81283), Anti-AKT (phospho T308) Antibody (ab38449), Anti-NF-κB p65 Antibody [E379] (ab32536), Anti-NF-κB p65 (phospho S536) Antibody [EP2294Y] (ab76302), Anti-JNK1 + JNK2 (phospho T183 + Y185) Antibody (ab4821), Anti-JNK1 + JNK2 + JNK3 Antibody [EPR16797-211] (ab179461), Anti-p38 alpha/MAPK14 Antibody [E229] (ab170099), Anti-p38 (phospho T180 + Y182) Antibody [EPR18120] (ab195049) were purchased from Abcam (Shanghai,

China). All other reagents were analytical grade and used without any further purification.

**2.2 Preparation of SeNPs**

0.8 mL of 0.012% (W/V) chitosan solution and 0.2 mL of 1 mmol·L$^{-1}$ $H_2SeO_3$ solution were mixed evenly. 0.8 mL of 4 mmol·L$^{-1}$ Vitamin C solution was added to the solution and diluted to 5 mL with distilled water. The selenium nanoparticles (SeNPs) were obtained by stirring at 200 rpm for 2 h at 25 °C.

**2.3 Synthesis of Oxidized Dextran**

12 mL of 0.5 mol·L$^{-1}$ periodate ($NaIO_4$) solution was added dropwise to 20 mL of 100 mg·mL$^{-1}$ dextran solution. The reaction was carried out at 25 °C, 600 rpm, protected from light for 4 h. After that, 1 mL of ethylene glycol was added to the solution, and stirring was continued for 15 min to terminate the oxidation reaction. The unreacted monomer was removed using a dialysis bag (MW = 3500). The solution is lyophilized to give oxidized dextran (OD). The chemical reaction formula is shown in Figure S2.

The content of aldehyde group in OD was directly determined by hydroxylamine hydrochloride titration, and the degree of oxidation was calculated. Briefly, 100 mg of OD was dissolved in 25 mL of hydroxylamine hydrochloride-methyl orange solution and stirred for 3 h at 25 °C. The control group used 100 mg of dextran. The consumption of sodium hydroxide (NaOH) was recorded by titration with 0.05 M standard NaOH solution until the color of the solution changed from red to yellow, the same color as that of the control group. Finally, the degree of oxidation is calculated.

**2.4 Synthesis of Phenylboronic Acid Grafted Polylysine**

750 μL of 20 mg·mL$^{-1}$ polylysine (PLL) in DMSO and 200 μL of 20 mg·mL$^{-1}$ 4-bromomethylphenylboronic acid in DMSO were mixed, and then 10 μL of Triethylamine was added. After reaction at 70 °C for 24 h, phenylboronic acid grafted PLL (PP) was obtained by dialysis and lyophilization. The synthesis reaction equation is shown in Figure S2.

**2.5 Fabrication of OD-PP@SeNPs Hydrogel**

A solution of 100 mg·mL$^{-1}$ OD was prepared in pH 7.4 PBS. The SeNPs were uniformly dispersed in pH 7.4 PBS, and PP was added. A 100 mg·mL$^{-1}$ PP solution containing SeNPs was obtained. The above two solutions were mixed in equal volumes to prepare the OD-PP@SeNPs hydrogel. The hydrogel without SeNPs are noted as OD-PP hydrogel.

**2.6 Characterization of SeNPs and OD-PP@SeNPs Hydrogel**

Bruker Vertex 70 spectrometer (Billerica, USA) and UV-1800–Vis spectrophotometer (Kyoto, Japan) was used to determine the chemical structures of the polymers and SeNPs. The grafting rate of OD was again calculated by $^{1}$H nuclear magnetic resonance (NMR) spectroscopy (Bruker Avance II, 400 MHz). The molecular weight of OD was determined by Agilent 1260 gel permeation chromatography (GPC, State of California, USA). The molecular weight of PP was detected by matrix-assisted laser desorption ionization time-of-flight mass spectrometry (Maldi-Top, METTLER TOLEDO, Switzerland). The particle size, PDI, and Zeta potential of SeNPs were measured by a ZS90 Malvern Zeta Nanosizer

(Malvern Instruments Ltd., Malvern, UK). After lyophilization, SeNPs powder was obtained (n = 3). The morphologies of the SeNPs were detected by transmission electron microscope (TEM; Hitachi H-600) and SPM-9700HT atomic force microscopy (AFM, Shimadzu, Japan). The morphologies of lyophilized hydrogels and SeNPs were observed by scanning electron microscopy (SEM, Thermo scientific Apreo-2C) and energy-dispersive spectrometry (EDS; Oxford Max-65). The elemental species of SeNPs and OD-PP@SeNPs hydrogel were analyzed by X-ray Photoelectron Spectrometer (XPS, Shimadzu, Japan). TGA of SeNPs and polymers were carried out on an STA 449 F3 simultaneous thermal analyzer (NETZSCH Group, German). DSC 3 (METTLER TOLEDO, Switzerland) was used to conduct a thermal analysis of all samples. The crystal form of SeNPs and polymers were evaluated by a D/max 2200VPC X-ray diffractometer (Rigaku Corporation, Japan).

The rheological measurements of hydrogels were performed on a Kinexus Lab+ rheometer (Malvern, UK) with a 25 mm parallel plate. The storage (G′) and loss (G″) moduli of the hydrogels were detected at 37 °C. The frequency sweep was measured with a scan frequency from 0.01 to 1 Hz at 0.5% constant strain in the linear viscoelastic region and assessed by strain sweep experiments for each sample. The strain sweep was measured from a small strain ($\gamma$ = 0.1%) to a large strain ($\gamma$ = 100%).

The adhesion performances of OD-PP@SeNPs hydrogel to the skin and common materials were determined. The adhesive strength of various substrates was measured and quantified. The OD-PP@SeNPs hydrogel were stained with Nile red and crystal violet stains. Regarding self-healing properties, the OD-PP@SeNPs hydrogel were

stained with Nile red and crystal violet stains. The hydrogel was cut into 2 pieces, and then the 2 pieces were reassembled into a new shape and placed at 25 °C for 20 min to allow it to self-heal into a complete hydrogel.

Swelling experiments of OD-PP and OD-PP@SeNPs hydrogels were performed in pH 7.4 PBS at 37 ° C. The swollen hydrogel was removed at the desired time. After removing the superficial water using filter paper, the weight of hydrogels was recorded as $W_t$. The initial weight of the lyophilized hydrogels was weighed as $M_0$ (n = 3). Swelling (%) = ($M_t$-$M_0$) / $M_0$ × 100%. For the *in vitro* degradation experiment, the degradation of the hydrogels was examined in pH 7.4 PBS, pH 6.5 PBS, 1 mM $H_2O_2$ pH 7.4 PBS, and 1 mM $H_2O_2$+pH 6.5 PBS, respectively. All samples were stored in different solutions at 25 °C for 14 d. The hydrogels were taken out and lyophilized at different times, and the weight was recorded as $M_d$. Remaining weight (%) = ($M_0$-$M_d$) / M0 × 100%. Photographs of the samples were taken at different time intervals.

Bovine serum albumin- Fluorescein Isothiocyanate (BSA-FITC) was incorporated into OD-PP and OD-PP@SeNPs hydrogels, respectively, and to prepare OD-PP@BSA-FITC and OD-PP@SeNPs@BSA-FITC. The BSA-FITC was added to PP at a concentration of 10% (W/W).

The release characteristics of BSA-FITC from OD-PP@BSA-FITC and OD-PP@SeNPs@BSA-FITC hydrogels were evaluated. Briefly, OD-PP@BSA-FITC and OD-PP@SeNPs@BSA-FITC hydrogels were immersed in pH 7.4 PBS, pH 6.5 PBS, 1 mM $H_2O_2$ pH 7.4 PBS, and 1 mM $H_2O_2$+pH 6.5 PBS, respectively. The

temperature was 37 °C and the rotation speed was 50 rpm. The release medium was collected at predetermined times while replenishing with fresh medium. The BSA-FITC release efficiency was measured by a Synergy H1 microplate reader (Biotek, Winooski, VT, USA).

To study SeNPs release from OD-PP@SeNPs hydrogel, OD-PP@SeNPs hydrogel were immersed in pH 7.4 PBS, pH 6.5 PBS, 1 mM $H_2O_2$ pH 7.4 PBS, and 1 mM $H_2O_2$+pH 6.5 PBS, respectively, at 37 °C and 50 rpm. The Se release efficiency was measured by ICP-MS (PlasmaQuant MS, Germany). The mechanism of SeNPs release from OD-PP@SeNPs hydrogel was determined using various kinetics models on the *in vitro* release data, which include zero-order, first-order, Korsmeyer-Peppas, and Higuchi (Table S3).

**2.7 Antioxidant Capacity of the Hydrogel**

The antioxidant capacity of hydrogel was determined by scavenging DPPH radicals, $O_2^{-}$• radical scavenging, and • OH radical scavenging.

The hydrogel of each group were homogenized in a tissue grinder. Then, 100 μM DPPH and different preparations were mixed and dispersed in 2.0 mL of ethanol with PBS as a blank group. The mixture was mixed and allowed to react for 30 min at 37 °C in the dark. The absorbance of the mixture was measured using UV-Vis spectrophotometer at 517 nm. The DPPH scavenging activity was calculated using the following equation: DPPH scavenging ratio (%) = $(A_b - A_h) / A_h \times 100\%$. In where, $A_b$ is the absorbance of the control (DPPH + ethanol. $A_h$ is the absorbance of the preparations (DPPH + methanol + sample).

100 μL of hydrogel or SeNPs solution was added to 100 μL of 100 μM ABTS solution. After mixing, the absorbance of the sample was detected at 413 nm.

The ability of the hydrogel to scavenge • OH radicals generated by the Fenton reaction was examined. Briefly, 100 mg of the sample was mixed with 500 μL of 1 mM $FeS0_4$ solution and 500 μL of 100 mM $H_2O_2$. The mixture was incubated at 37 °C for 1 h. After cooling to 25 °C, 100 μL of the supernatant was mixed with 100 μL of 10 mM 3355-tetramethylbenzidine (dissolved in DMSO) and the absorbance at 660 nm was measured by a microplate reader.

**2.8 *In Vitro* Antibacterial Activity Assays**

*E. coli* (CGMCC 1.3373) and *S. aureus* (CGMCC 1.0089) were purchased from Biotechnology Research Institute, CAAS. These bacteria were incubated with Luria-Bertani (LB) broth at 37 °C, and then the concentration of each bacterial suspension was adjusted to $1 \times 10^8$ CFU·mL$^{-1}$. The hydrogel antibacterial experiments were divided into four groups: (1) Bacteria, (2) Bacteria + SeNPs, (3) Bacteria + OD-PP hydrogel, (4) Bacteria + OD-PP@SeNPs hydrogel.

Antibacterial activity: 100 μL of $1 \times 10^6$ CFU·mL$^{-1}$ bacterial suspensions were added to 96-well culture plates with different preparations. After incubating for 12 h at 37 °C, the OD value of the suspensions was detected at 600 nm by a microplate reader. Antibacterial ratio (%) = $(N_0 - N_p)/N_0 \times 100\%$. In where, the OD value of the blank group was noted as $N_0$, and the experimental group OD value was $N_p$.

Colony formation test: The sterilized hydrogels with or without SeNPs were first soaked in pH 7.4 PBS at 37 ° C for 24 h to obtain hydrogel extracts (200 mg·mL$^{-1}$).

The bacteria were mixed with PBS and 100 μL of the obtained bacterial suspension was applied to LB agar plates. The plates were then incubated for an additional 12 h at 37 °C to form observable colony units and photographs of the LB plates were taken. The blank group was solid agar medium. The Model was the bacterial group without the addition of preparation.

Bacterial aggregation: The sterilized hydrogels with or without SeNPs were first soaked in pH 7.4 PBS at 37 ° C for 24 h to obtain hydrogel extracts (200 mg·mL$^{-1}$). Bacteria were mixed with PBS to obtain bacterial suspensions. The bacteria were incubated at 37 °C for 12 h. Bacterial aggregation was observed and photographs of the aggregates were taken.

Inhibition zone test: The antibacterial properties of hydrogels were detected by the disc diffusion method. First, 100 μL of the bacterial suspension at a density of 1 × $10^6$ CFU·mL$^{-1}$ was evenly spread onto the agar plates. Thereafter, circular paper (6 mm in diameter) soaked in the different samples was gently placed on agar plates and incubated at 37 °C for 12 h. Finally, the growth inhibition zone around each group of samples was photographed, and the antibacterial activity was determined by measuring the diameter of the inhibition zone generated around the samples.

Live-Dead bacterial staining: Bacteria were incubated with the different samples at 37 °C for 12 h before being stained by Live & Dead Bacterial Staining. In short, the 1 mL of bacterial suspension was stained with Live & Dead Bacterial Staining Kit (YEASEN Biotech Co., Ltd., China) for 30 min in dark at 25 °C. After washing twice with PBS, the stained bacterial solution was dropped on the slides, and fluorescence

images were captured with a Cytation5 (Biotek, Winooski, VT, USA).

**2.9 Cell Research**

RAW264.7, L929, and HUVECs cells were selected for cell research. Subsequent cellular studies were performed using lyophilized SeNPs, OD-PP, and OD-PP@SeNPs hydrogels. The freeze-dried and sterilized hydrogels (200 mg, containing 2 mmol SeNPs) were immersed in complete DMEM and extracted for 24 h at 37 °C. The medium containing the hydrogel was obtained for further use.

Screening of Inducer Concentration: As previously reported, LPS was frequently used to induce RAW264.7 cells and $H_20_2$ to induce HUVECs cells so that they become inflammatory cells. The concentration of inducers was screened using cellular activity and inflammatory indicators (NO and ROS). Briefly, RAW264.7 cells were seeded into 96-well plates at a density of $6 \times 10^3$ cells·well$^{-1}$. After 12 h of culture, different concentrations of LPS were added to induce for 24 and 48 h. And HUVECs cells were seeded into 96-well plates at a density of $1 \times 10^4$ cells·well$^{-1}$. After 12 h of culture, different concentrations of $H_20_2$ were added to induce for 24 h. Then, the mediums were abandoned and cells were incubated with 100 μL of serum-free mediums containing 10% (V/V) CCK-8 reagent. Besides, the absorbance was detected at 450 nm using MULTSIKANMK3 multifunctional enzyme marker (Thermo Fisher, USA) (n = 6). The release of NO as nitrite was also measured by a Nitric Oxide Detection Kit (NO; Beyotime Biotech Inc, S00215, China).

Biocompatibility test: To evaluate the cytotoxicity of preparations, RAW264.7 cells were seeded into 96-well plates at a density of $5 \times 10^3$ cells·well$^{-1}$ and activated

by LPS for 24 h. And L929 and HUVECs cells were seeded into 96-well plates at a density of 5 × $10^3$ cells·$well^{-1}$ and activated by $H_2O_2$ for 24 h, respectively. After removing the medium, the cells were incubated with different formulations for 24 and 48 h. Then, the mediums were abandoned and cells were incubated with 100 μL of serum-free mediums containing 10% (V/V) CCK 8 reagent. Besides, the absorbance was detected at 490 nm using an enzyme marker (n = 6). The cells with oxidation induction were used as the model group, and the cells without any treatment (no oxidant or preparation) were used as the control group.

Live & Dead cell staining: Live & dead cell staining experiments were performed to assess biocompatibility further. Briefly, different cells (RAW264.7, L929, and HUVECs) were seeded separately on 12-well plates. The cells were activated by LPS or $H_2O_2$ for 24 h as the Model group. The cells were incubated in a medium (containing hydrogel) for 24 h. The cells were stained with 500 μL Calcein-AM/propidium iodide and then further co-cultured at 37 °C for 30 min. The well plates were washed 3 times with PBS for 1 min each. Finally, cells were observed with a CLSM (Biotek, Winooski, VT, USA) and the apoptosis rate was quantitatively detected by flow cytometry.

ROS staining: The cell-permeable DCFH-DA was applied to measure the ROS levels in cells. Briefly, RAW264.7 and HUVECs were seeded separately in 12-well plates at a density of 1 × $10^5$ cells·$well^{-1}$. The cells were activated by LPS or $H_2O_2$ for 24 h. Cells were co-cultured with different preparations for 24 h. After PBS washing three times, RAW264.7 and HUVEC were treated with 0.5 mL of 10 μmol·$L^{-1}$

DCFH-DA in the free-medium for 30 min in the cell incubator, respectively. The fluorescent images were visualized by an CLSM. The cells were analyzed on flow cytometry to quantify the results, with FlowJo X Software to deal with the data.

Cell scratch: The process of cell migration was simulated by scratching the $H_2O_2$-induced HUVECs cell monolayer. Briefly, $1 \times 10^5$ cells·well$^{-1}$ HUVECs cells were seeded in 12-well culture plates for 6 h to form a cell monolayer. Then $H_2O_2$ was added and incubated for 24 h. Subsequently, the cell monolayer was scraped using a 20 μL pipette and washed twice with PBS. The cells were incubated with different preparations (500 μL), and cell images were captured by a microscope at specific times (0, 12, 24, and 36 h). Scratch healing rate (%) = $(C_0 - C_t) / C_0 \times 100\%$. In where, $C_0$ and $C_t$ separately represent the scratch area before and after an intervention.

Vertical migration: HUVECs cells ($1 \times 10^5$ pcs·well$^{-1}$) were seeded in the upper chamber of the transwell (costar 3422, 8.0 μm pore size). 200 μL of media containing different formulations were added continuously. After 12 h of incubation, the non-migrated cells in the upper chamber were removed with a cotton swab. The cells on reverse side were gently rinsed with PBS and fixed in 4% paraformaldehyde. The cells were stained with a crystal violet staining solution (300 μL) for 30 min. Then, PBS was used to wash away the excess crystalline violet staining solution. The cells were observed under an inverted microscope and the characteristics of vertical migration were evaluated.

Tube formation assay: 50 μL of Matrigel was added to precooled 96-well plates, then placed at 37 °C in a 5 % $CO_2$ ambiance for 1 h. HUVECs were seeded into well

plates at a density of 4 × $10^3$ cells·well$^{-1}$. The cells were incubated with different preparations (200 μL). After 6 h, the cells were stained by an Angiogenesis Assay Kit. Tube formation images were obtained by an inverted fluorescence microscope.

Determination of cytokines: The levels of various cytokines produced by the cells were measured to assess the anti-inflammatory activity of the formulation. RAW264.7 macrophages were pre-treated with LPS for 24 h and HUVECs were pre-cultured with $H_2O_2$ for 24 h. Afterward, different samples (SeNPs or hydrogel and DMEM) were added for further incubation for 24 h. The supernatant was gently aspirated and collected. The levels of inflammatory cytokines (TNF-α, IL-1β, IL-6, and IL-10) secreted by RAW264.7 and HUVECs cells were determined with an enzyme-linked immunosorbent assay (ELISA) kits (Enzyme-Link Biotech, Shanghai, China). The therapeutic effect of hydrogel on inflammation in RAW264.7 cells was preliminarily researched, and the antioxidant mechanism of OD-PP@SeNPs hydrogel on LPS-induced RAW264.7 cells was explored. The levels of SOD, GPx, and MDA were measured by the respective kits.

Immunofluorescence: HUVECs were divided into the Control group, Model group ($H_2O_2$), SeNPs group, OD-PP group, and OD-PP@SeNPs group. HUVECs were seeded on sterile coverslips and cultured for 12 h. HUVECs were induced by $H_2O_2$ and then incubated with the different formulations for 24 h. Cells were fixed with 4% paraformaldehyde for 30 min. Then, the HUVECs cells were incubated with primary antibodies against VEGF (1:200) or CD31 (1:200) at 4 °C overnight and then incubated with respective fluorescent-labeled secondary antibodies for 60 min. The

nuclei were stained with DAPI for 10 min. The immunofluorescence images of the cells were taken by a CLSM.

Macrophage phenotype transition study: Polarization of macrophages was characterized by immunofluorescence staining. RAW264.7 were seeded in 6-well culture plates and cultured for 12 h. RAW264.7 were induced by LPS and then incubated with the different formulations for 24 h. Then, RAW264.7 were permeabilized with Triton X-100 for 20 min. Next, they were blocked with 5% goat serum at 37 °C for 1 h. Next, cells were incubated with CD86 (1:400) and CD206 (1:500) overnight at 4 °C, followed by a fluorescent secondary antibodies incubation for 60 min. The nuclei were stained with DAPI for 10 min. The immunofluorescence images of the cells were taken by a CLSM.

Western Blot: RAW264.7 and HUVECs were seeded separately in 6-well plates at a density of $1 \times 10^5$ cells·well$^{-1}$. The cells were activated by LPS or $H_2O_2$ for 24 h. Cells were co-cultured with different preparations for 24 h. The cells were then collected and lysed using RIPA (with PMSF) in an ice bath. The sample protein concentrations were determined using the BCA protein assay kit. The proteins were separated by SDS-PAGE and then transferred to PVDF membrane. After blocking at 25 °C for 1 h, the PVDF membrane was incubated overnight at 4 °C with specific primary antibodies. After overnight, incubated with the secondary antibodies 1 h at 25 °C. Finally, PVDF films were stained with ECL luminescent agent in a dark environment for 10 min. The intensity of the blot was measured by Image J software.

### 2.10 *In Vivo* Responsiveness and Retention

Subcutaneous: The inflammatory model was established by subcutaneous injection of 0.1 mL 10 mg·mL$^{-1}$ LPS in the abdomen of rats. 0.5 mL of OD-PP@SeNPs hydrogel loaded with IR820 was injected subcutaneously into the abdomen of rats, and the retention of the hydrogel was observed by the small animal living imaging system. The body weight of the rats was recorded. After the hydrogel was completely metabolized, 1 mL of blood was taken from the fundus venous plexus of rats for blood routine and biochemical analysis. After the rats were euthanized, the subcutaneous tissues at the hydrogel injection site were taken and fixed in 4% paraformaldehyde. H&E staining was performed to observe whether the subcutaneous tissues were damaged. The hearts, livers, spleens, lungs, and kidneys were taken and stained with H&E and observed for any inflammation.

Joint Cavity: The inflammatory model was established by joint injection of 0.05 mL 10 mg·mL$^{-1}$ LPS in the rats. 0.05 mL of OD-PP@SeNPs hydrogel loaded with IR820 was injected subcutaneously into the joint cavity of rats, and the retention of the hydrogel was observed by the small animal living imaging system. The body weight of the rats was recorded. After the hydrogel was completely metabolized, 1 mL of blood was taken from the fundus venous plexus of rats for blood routine and biochemical analysis.

**2.11 Healing of Infected Wounds *in Vivo***

The rats were anesthetized by inhalation and the hair on the back was clipped. Rats with intact skin were selected to establish a wound model. Under anesthesia, a 0.8 cm diameter skin wound was obtained in the middle of the rat's back using a

punch, and S. aureus suspension (10 μL, $1\times10^8$ $CFU\cdot mL^{-1}$) was added dropwise for bacterial infection. After 24 h, the rats were randomly divided into the Model group, SeNPs group (0.5 mL), OD-PP hydrogel group (0.5 mL), OD-PP@SeNPs hydrogel group (0.5 mL), and Tegaderm group. There were 15 rats in each group. The wounds were treated according to the experimental groups. The body weight and wound healing were observed and recorded according to the experimental times. In addition, a Control group of six healthy rats was set up. Wound healing was observed and photographed at 0, 4, 7, 10, and 14 d, and the wound contraction rate (%) was determined by Image J software (National Institutes of Health).

To evaluate the anti-inflammatory and pro-wound healing effects of OD-PP@SeNPs hydrogel, the experiments of H&E staining, Masson staining, ROS staining, immunofluorescence staining, Elisa and RT-PCR were carried out. Three rats were randomly selected from each group on the 7th and 14th d after injury. After euthanasia, the wound tissue was cut. The obtained wound tissue was divided into two parts. One part was fixed with 4 % paraformaldehyde solution for histopathological analysis, and the other part was prepared into skin homogenate for the determination of inflammatory cytokines and antibacterial properties. The isolated wound samples were homogenized with sterile saline to get 20% (W/V) homogenates. The wound homogenates were centrifuged at 5000×g, 4 °C for 10 min. Granulation tissue gap, epithelial thickness, hair follicle density, and re-epithelialization were observed according to H&E staining. Collagen deposition was assessed by Masson staining. The protein levels of TNF-α, IL-1β, IL-6, and IL-10 were evaluated by Elisa. To

further evaluate the efficacy of the hydrogel, angiogenesis in wound area was evaluated by immunofluorescence staining of VEGF, CD31, HIF-1, and α-SMA.

The infected tissues were collected and homogenized with 2 mL of LB broth under sterile conditions. After dilution with a liquid medium, the bacteria were cultured at 37 °C for 12 h, and the OD value was detected at 600 nm by a microplate reader.

The expression of inflammation-related genes (*tnf-α, il-1β, il-6, and il-10*) was quantified using RT-PCR. The total RNA from the skin was extracted with a reagent (Invitrogen, USA), and the purity and concentration of the RNA were determined. A Revert Aid First Strand cDNA Synthesis Kit and 1 μg of RNA were used to synthesize cDNA. The cDNA was amplified by RT-PCR with a SYBR Premix Ex Tag Kit, employing an PRISM 7500 sequencing detection system (ABI, USA). The mRNA levels of *tnf-α, il-1β, il-6, and il-10*, and GAPDH were quantified and normalized to GAPDH. The primer pairs for RT-PCR are presented in Table S4.

After deparaffinization, rehydration, and antigen retrieval, the sections of the wound were incubated with primary antibodies against CD86 (1:100) and CD206 (1:100) at 4 °C overnight and then incubated with fluorescent-labeled secondary antibodies for 60 min. After being counterstained with DAPI, the skin sections were imaged using a fluorescent microscope.

**2.12 Rheumatoid Arthrisis (RA)**

To induce CIA, rats were subcutaneously injected with bovine type II collagen (2 mg·mL$^{-1}$) and incomplete Freunds adjuvant (CFA, 2 mg·mL$^{-1}$). About 200 μL of the

emulsion was injected into rat tails as initial immunizations. After 7 d, rats were again immunized with 100 μL emulsion. The success of model establishment was evaluated by inflammatory scores of rat joints (Table S5). After 21 d, the rats were randomly divided into the Model group, SeNPs group (0.05 mL), OD-PP hydrogel group (0.05 mL), OD-PP@SeNPs hydrogel group (0.05 mL), and Positive group (Methylethalin, 0.5 mL, 1.8 $mg \cdot kg^{-1}$). There were 9 rats in each group. The wounds were treated according to the experimental groups. The healthy rats served as the Control group (n = 6). The frequency of administration was once every 10 d, and the administration cycle was 35 d. The body weight, paw thickness, and arthritis score of rats were recorded every 5 d.

The therapeutic effect of hydrogel on RA rats was evaluated by a balance beam experiment. The balance beam is 5 cm wide and 40 cm long. The time required for rats to walk from one end to the other end was recorded, and the morphology of rats during walking was captured by video.

X-ray imaging was performed on the rat toes and ankle joints in order to assess bone destruction. The differences in inflammation between the groups were evaluated from bone erosion, joint space, and bone destruction.

After treatments, all the rats were sacrificed. The ankle joints and main organs (heart, liver, spleen, lung, and kidney) of some rats were collected and fixed in 4% paraformaldehyde. The ankle joints were decalcified with 10% EDTA solution for 30 d at 25 °C. Samples were embedded in paraffin and sliced into 4 μm thick sections. The sections of ankle joints and main organs (heart, liver, spleen, lung, and kidney)

were stained with H&E staining. And the sections of ankle joints were Masson staining, TUNEL staining, ROS staining, immunohistochemistry, and immunofluorescence staining.

After the end of treatment, the spleen and thymus were collected. Adipose tissue adhering to the surface was removed, tissue fluid was aspirated on filter paper, and the weight was recorded. Organ index = Organ weight (mg) / Body weight (g)

The blood samples were collected at the study endpoint. The Elisa and RT-PCR were used to determine the inflammatory cytokines and mRNA expression. The specific operation is the same as "2.15.4 RT-PCR".

After deparaffinization, rehydration, and antigen retrieval, the sections of ankle joints were incubated with primary antibodies against CD86 (1:100) and CD206 (1:100). The specific operation is the same as "2.15.5 Macrophage Polarization".

**2.13 *In Vivo* Toxicity**

Pharmacological safety is a key factor to be considered for innovative preparations. Blood compatibility of SeNPs and OD-PP@SeNPs was evaluated by spectrophotometry. Briefly, 10 mL of fresh rat blood was centrifuged at 3,000 rpm for 10 min to separate red blood cells (RBCs). RBCs were repeatedly washed with saline. The different concentrations of SeNPs and OD-PP@SeNPs were added to 2% (V/V) RBCs, respectively. Incubation was done at 37 °C for 2 h. Negative and positive controls were obtained by mixing RBCs with PBS and deionized water, respectively. Subsequently, the samples were centrifuged at 1,000 rpm for 10 min. Approximately 100 μL of supernatants for each sample were used to measure absorbance using a

microplate reader at a wavelength of 540 nm (n = 3). Hemolysis (%) = ($A_{sc}$ - $A_{nc}$) / ($A_{pc}$ - $A_{nc}$). In where, $A_{sc}$, $A_{nc}$, and $A_{pc}$ represent the absorbances of the sample, negative control (-) and positive control (+) controls, respectively.

To evaluate the safety of each preparation, the serum of rats was collected after treatment, and the levels of aspartate aminotransferase (AST), alanine aminotransferase (ALT), blood urea nitrogen (BUN) and creatinine (Cre) were measured by using the standard kits according to the manufacturer's instructions. The heart, liver, spleen, lung, and kidney of rats were stained for H&E.

**2.14 Statistical Analysis**

All data were presented as mean ± standard deviation (SD) and each experiments was performed at least three times. Statistical analysis was tested with the Prism 7.0 software (GraphPad Software) by Tukey's multiple comparison tests and one-way analysis of variance (ANOVA). The differences were considered significant, when p values $^{\#} < 0.05$, $^{\#\#} < 0.01$, and $^{\#\#\#} < 0.001$.

**3. Discussion**

We prepared OD-PP@SeNPs hydrogel with a triple-network structure for the first time and sought to exploit the properties of the biomaterial itself to modulate the inflammatory microenvironment, treat IDs, and illustrate the mechanism behind ROS clearance. The hydrogel based on Schiff base bonds, Phenylboronate ester bonds, and hydrogen bonds has ideal mechanical properties, self-healing property, injectable, adhesiveness, good flexibility, swelling capacity, biodegradability, excellent stimuli-responsive active substance release performance, and excellent

biocompatibility. Schiff base bonds and Phenylboronate ester bonds are specifically broken with pH and ROS. This process can reshape the inflammatory microenvironment. Introduction of SeNPs into the OD-PP@SeNPs hydrogel enhanced the antioxidant capacity, improving its mechanical properties and self-healing ability *via* re-configuration of various bonds. OD-PP@SeNPs hydrogel has excellent stimuli-responsive SeNPs release performance, intelligent inhibition of bacterial proliferation, and regulation of inflammatory response to achieve a long-term therapeutic effect. The release of active SeNPs is accelerated when the pH of the inflammation site decreases. In addition, the SeNPs can scavenge ROS keeping the intracellular redox homeostasis and reducing oxidative stress, and eradicate bacteria to avoid infection, abilities that were boosted by OD-PP@SeNPs hydrogel. Meanwhile, Schiff base bonds and Phenylboronate ester bonds consume ROS and adjust pH during covalent bond cleavage to achieve the effect of modulating the inflammatory microenvironment. This allows the hydrogel to have multifunctional activities to be antioxidant, anti-inflammatory, inhibit bacterial infection, and scavenge ROS. The presence of SeNPs, Schiff base bonds, and Phenylboronate ester bonds together enhances the pharmacological efficacy of the OD-PP@SeNPs hydrogel.

Achieving high antimicrobial efficacy and low drug resistance of hydrogels remains a challenge. Some hydrogels do not exhibit inherent antibacterial characteristics. Metal nanoparticles are a promising alternative. The antimicrobial impact of metal materials is assigned to their structure/surface properties and charges

type, a synergistic effect of SeNPs, besides the bacteria structural characteristics and the negative charge of the bacteria surface. In another word, due to the high porosity of the cell wall and the most negative bacteria, the electrostatic attraction of the positively charged NPs to the surface of the cell wall can facilitate permeation, therefore increasing the bactericidal effect. [1] The antibacterial mechanism of SeNPs is their adhesion to the bacteria surface and change of cell wall integrity, which is followed by diffusion of Se into the cells. [2] This increases oxidative stress by generating a high level of intracellular ROS, which inhibits protein synthesis and causes DNA mutations. At last, cytoplasmic contents leaked, cell walls were damaged and bacteria death occurred. [3] SeNPs can be attached to the polymer surface in a better dispersed state. The OD-PP@SeNPs hydrogel provides continuous control of SeNPs release and prolongs the antimicrobial time. The OD-PP@SeNPs hydrogel with inherent antimicrobial properties provides long-lasting antimicrobial activity and biocompatibility. Catechol is a phenolic compound that has recently been shown to be an effective broad-spectrum disinfectant. [4] PP affects the structure of the bacterial cell wall through the interaction between cationic groups and the negative charge on the bacterial surface leading to the efflux of bacterial contents. Combining antibacterial agents and inherent antibacterial activity endows the OD-PP@SeNPs hydrogel with considerable antibacterial effects to control infection.

To date, various IDs have seriously affected the quality of life of people. Traditional therapies are based on anti-inflammatory and immunosuppressive drugs and are palliative with short-term remission. In addition, the complex pathogenesis

has not been fully elucidated, and IDs cannot be cured from the root. Previous studies suggest inflammation-related signaling pathways, including PI3K/Akt/NF-κB and MAPK. Therefore, suppression of these pathways is an effective and crucial therapeutic approach for treating IDs. Metal-based NPs are emerging nanocarriers for anti-inflammatory applications. [5] This study showed that OD-PP@SeNPs hydrogel significantly inhibited PI3K/Akt/NF-κB and MAPK signals, as evidenced by the inhibition of TNF-α, IL-1β, IL-6, iNOs, and COX-2 inflammatory cytokine secretion and decreased TNF-α, IL-1β, and IL-6 gene levels, increased the infiltration of M2 macrophages and anti-inflammatory factors secretion. This is mainly due to Schiff base bonds, Phenylboronate ester bonds, and SeNPs. Schiff base bonds and Phenylboronate ester bonds respond to acidic environments with high ROS, consuming ROS and adjusting pH during covalent bond cleavage. Selenium and selenoproteins play a key role in regulating cellular metabolism and their antioxidant and anti-inflammatory properties. [6] There is evidence that Se can reduce inflammation in autoimmune diseases. [7] Qin et al. demonstrate that selenium supplementation reduces ROS and accelerates the healing of RA disease in mice. [8] The concentration of SeNPs was also screened for antioxidant and anti-inflammatory effects. SeNPs are more effective than other forms of Se in increasing selenoprotein expression and scavenging free radicals. And SeNPs have been studied in different inflammation and redox imbalance-mediated disorders, such as arthritis, diabetes, nephritis, and cancer, and showed potential remedial uses. [9] To our knowledge, this is the first study of SeNPs and SeNPs-containing polymers exerting anti-inflammatory

effects via the PI3K/AKT/NF-κB and MAPK signaling pathways. It also proved that OD-PP@SeNPs hydrogel with ROS scavenging and pH-regulating ability protects cells from oxidative stress and induces macrophages into M2 polarization to reduce inflammatory cytokines through PI3K/AKT/NF-κB and MAPK pathways, exerting anti-inflammatory effects and modulating the inflammatory microenvironment.

Unfortunately, no biomaterial is perfect, and the same is true for OD-PP@SeNPs hydrogel. OD-PP@SeNPs hydrogel can only scavenge ROS in the local environment, restore local pH, and regulate the inflammatory microenvironment. Compared with those antioxidant nanomaterials that target macrophages, OD-PP@SeNPs hydrogel can hardly inhibit oxidative stress at the source of excessive ROS production. Whatever, local delivery systems with low toxicity, high efficiency, and controllable dose also have high development potential, providing a personalized treatment for IDs.

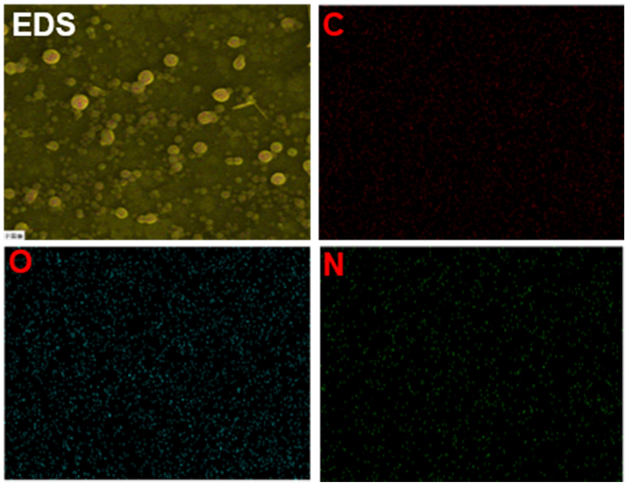


**Figure S1** EDS elemental mapping of SeNPs

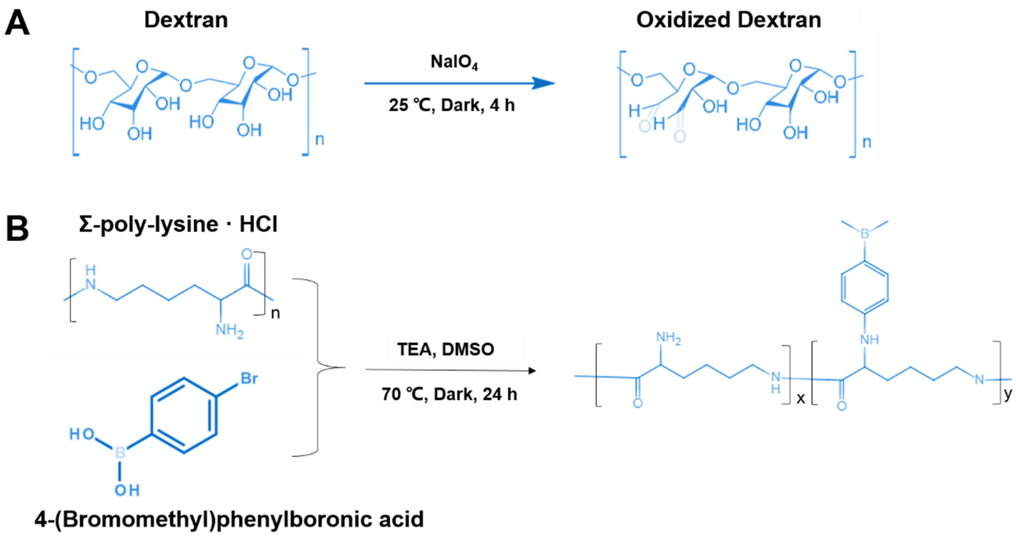


**Figure S2** Synthesis of hydrogel materials. (A) Oxidized dextran (OD) and (B) phenylboronic acid grafted polylysine (PP).

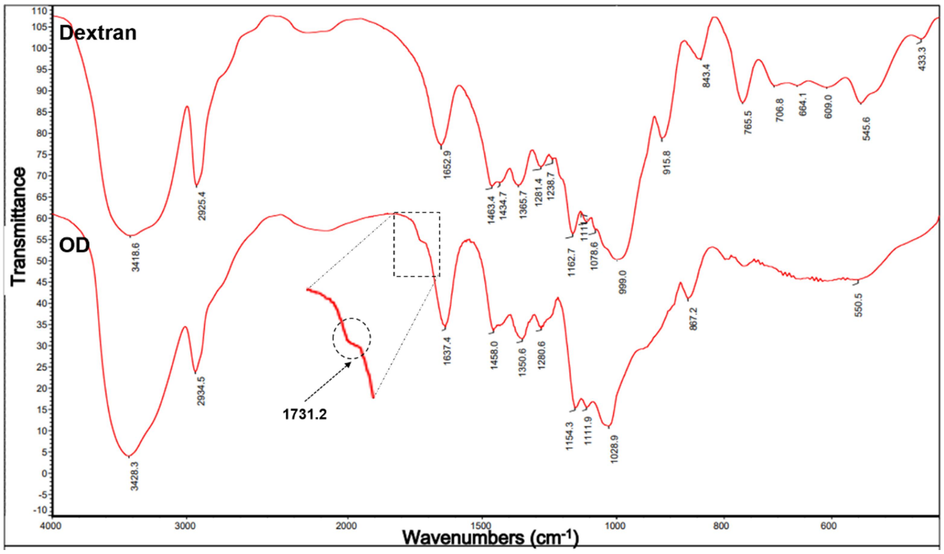


**Figure S3** FT-IR spectrum of OD

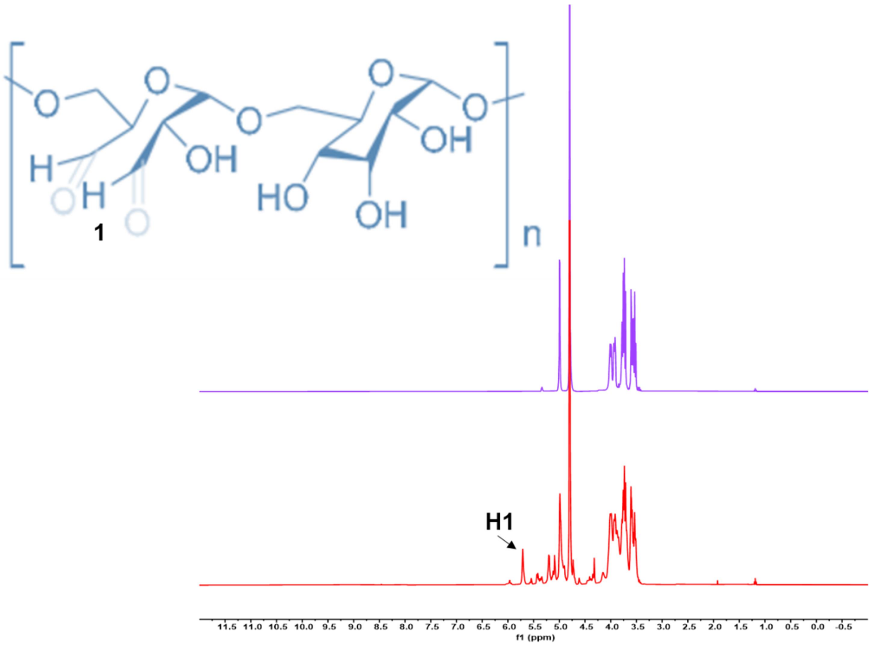


**Figure S4** $^{1}$H-NMR spectrum of OD

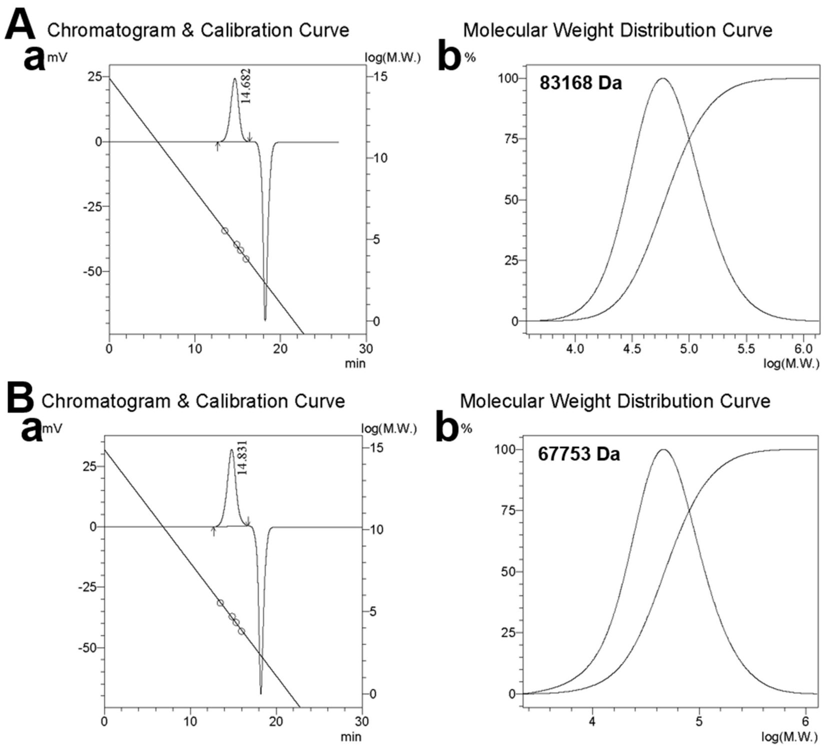


**Figure S5** GPC spectrum of (A) dextran and (B) OD

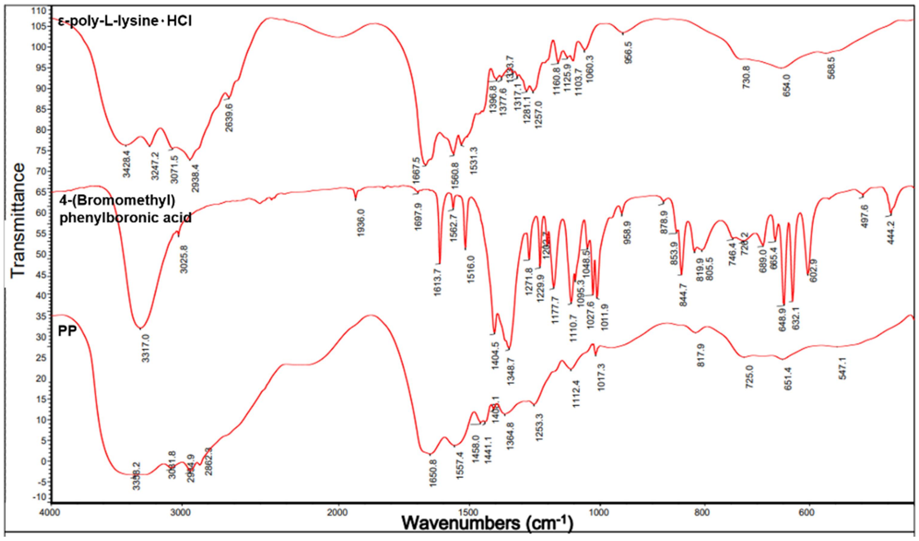


**Figure S6** FT-IR spectrum of PP

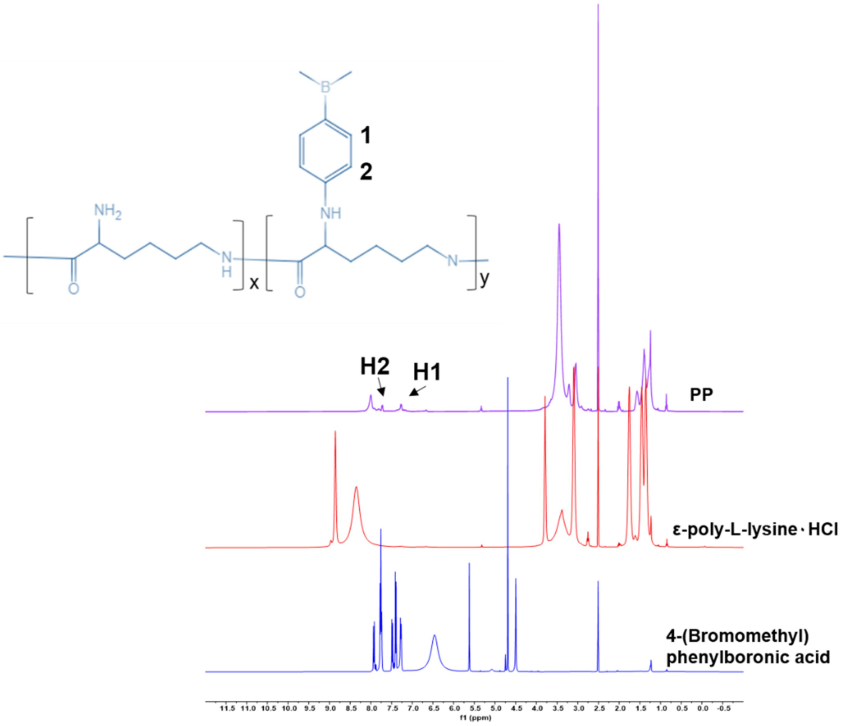


**Figure S7** $^{1}$H NMR spectrum of PP

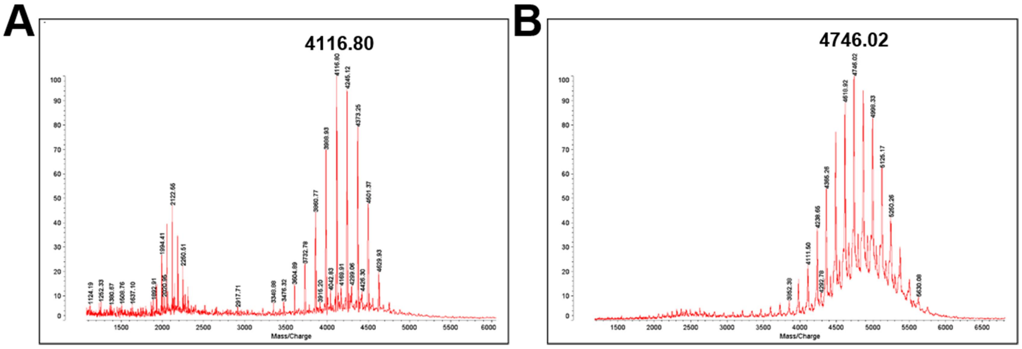


**Figure S8** Maldi-Top of (A) polylysine and (B) PP

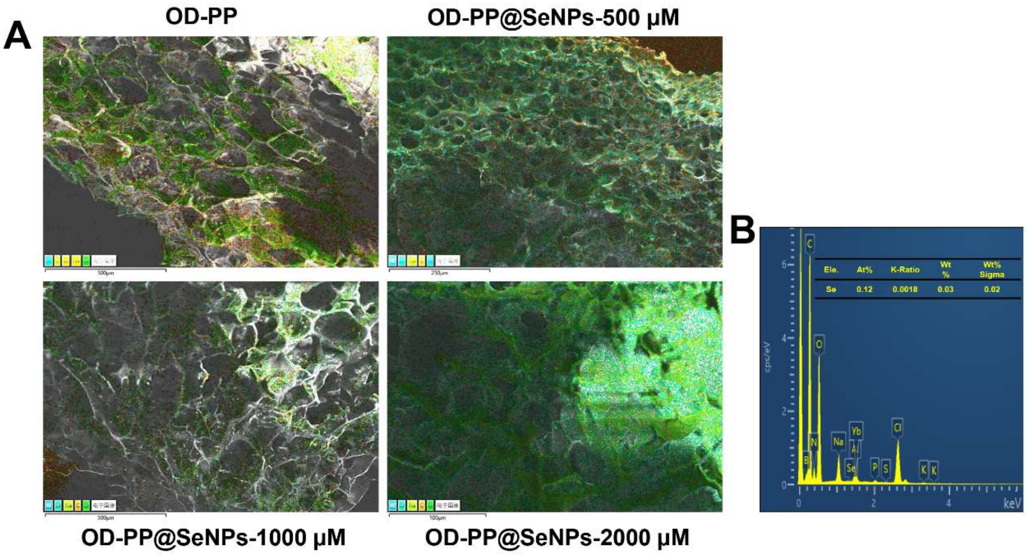


**Figure S9** Representative EDS image of hydrogel. (A) EDS elemental mapping of the hydrogels. (B) EDS spectrum of OD-PP@SeNPs hydrogel

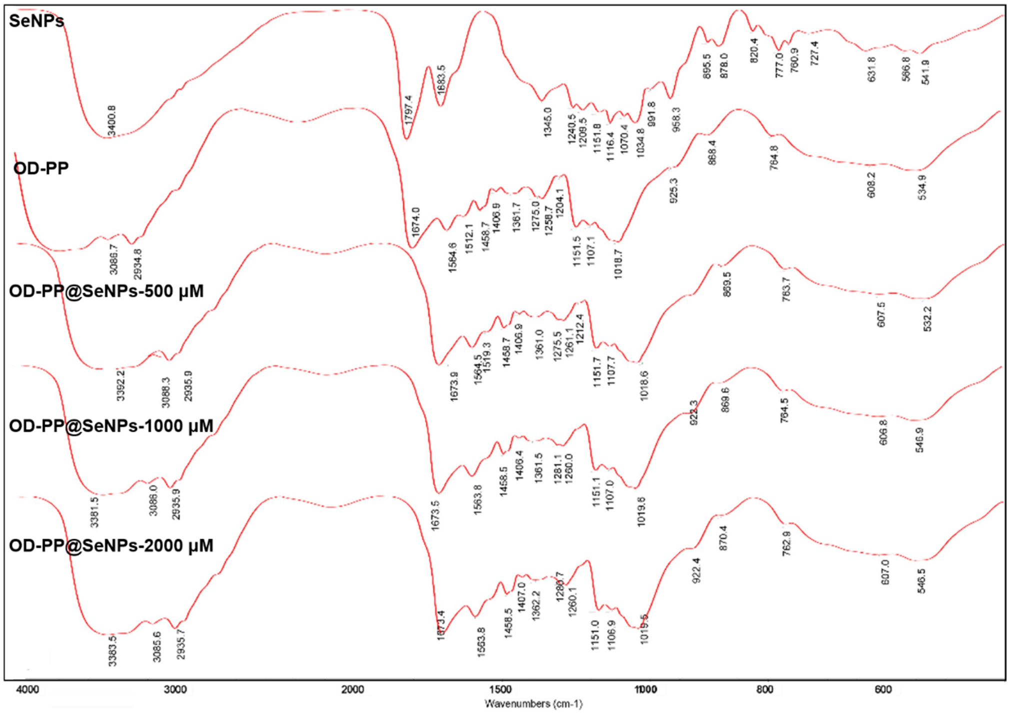


**Figure S10** FT-IR spectrum of OD-PP hydrogel and OD-PP@SeNPs hydrogel

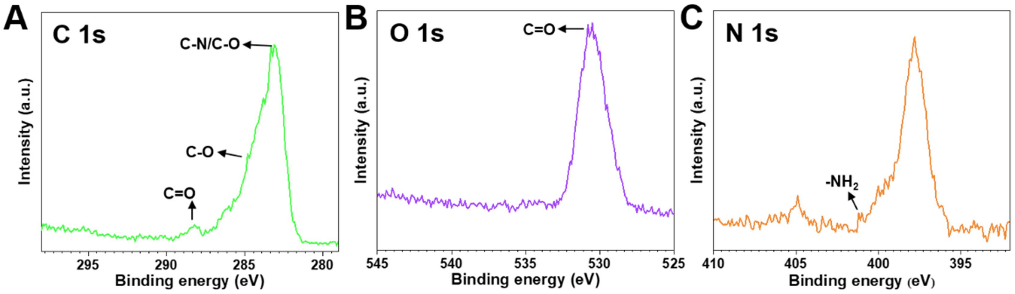


**Figure S11** XPS spectrum of OD-PP@SeNPs hydrogel

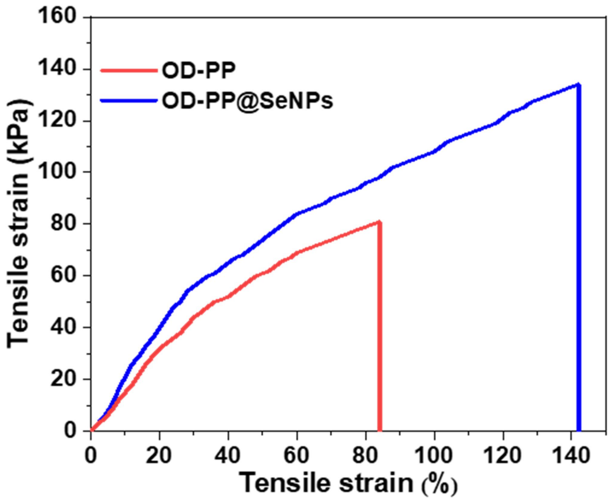


**Figure S12** Viscosity test of hydrogels with various shear rates.

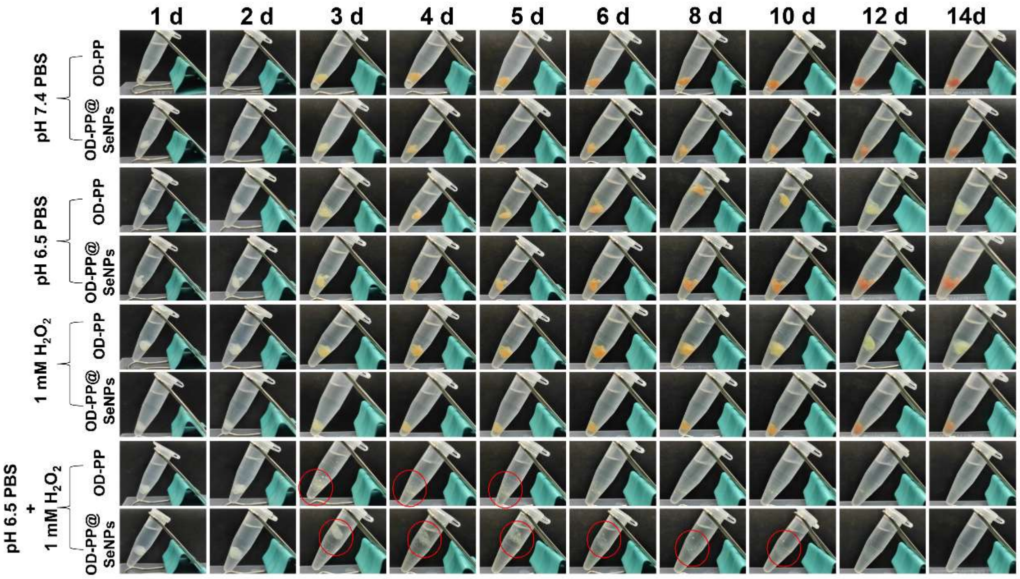


**Figure S13** Representative pictures of OD-PP and OD-PP@SeNPs hydrogels degradation *in vitro*

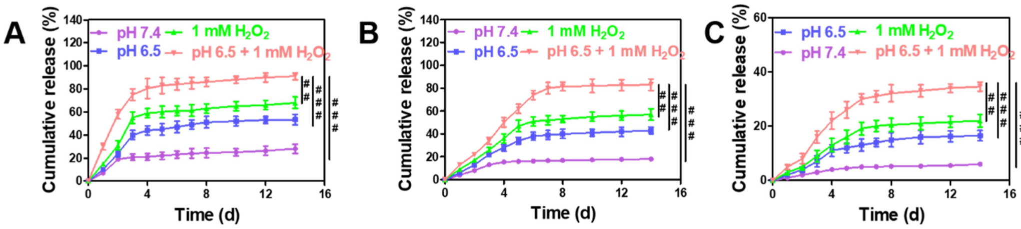


**Figure S14** The BSA-FITC cumulative release of (A) OD-PP hydrogel and (B) OD-PP@SeNPs hydrogel under different conditions at different time (n = 3). (C) *In vitro* release profiles of SeNPs from OD-PP@SeNPs hydrogel (n = 3)

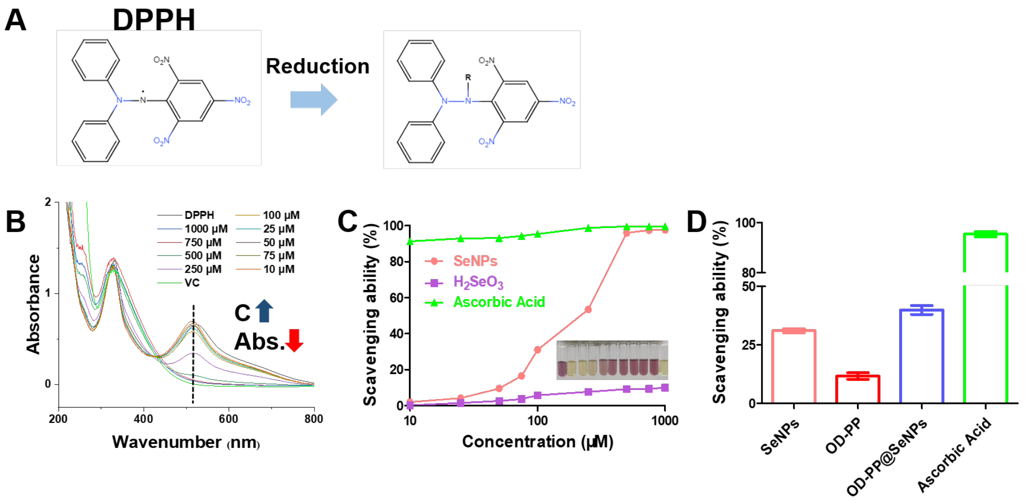


**Figure S15** DPPH. Mechanism of DPPH scavenging assays (A). DPPH assay absorbance curves (B). Scavenging ratio of SeNPs (C). Scavenging ability of OD-PP@SeNPs hydrogel (n = 3) (D)

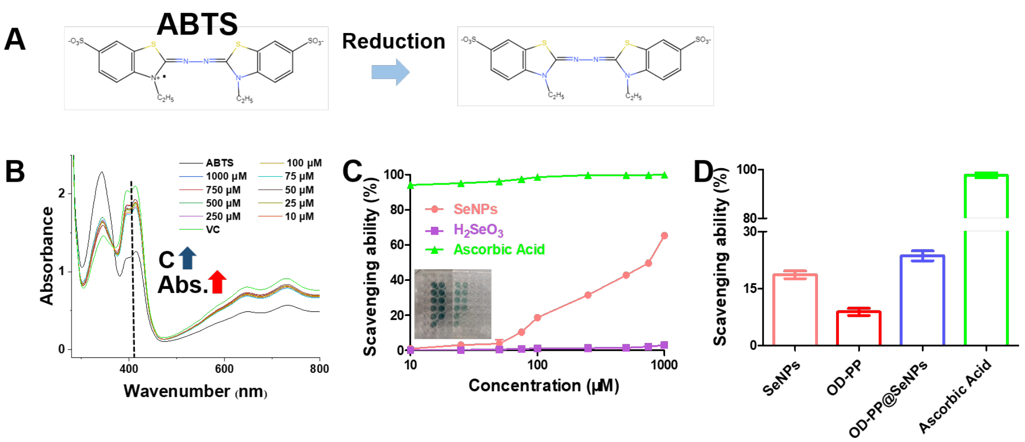


**Figure S16** ABTS. Mechanism of ABTS scavenging assays (A). ABTS assay absorbance curves (B). Scavenging ratio of SeNPs (C). Scavenging ability of OD-PP@SeNPs hydrogel (n = 3) (D)

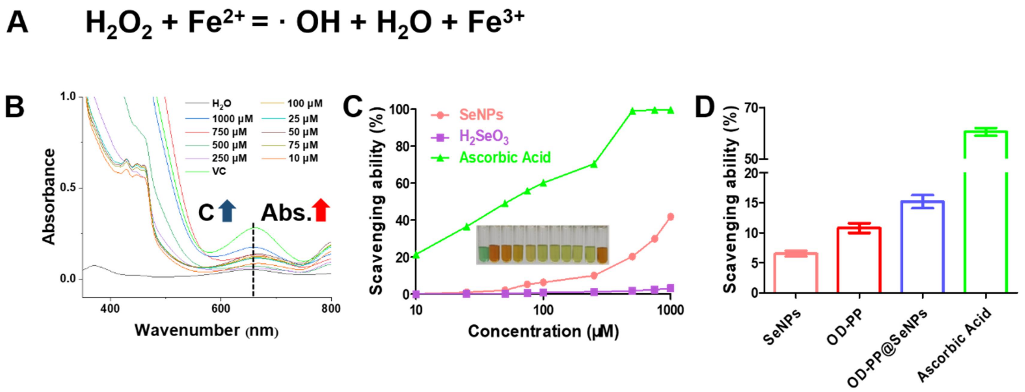


**Figure S17** Hydroxyl. (A) Mechanism of • OH scavenging assays. (B) • OH assay absorbance curves. (C) Scavenging ratio of SeNPs. (D) Scavenging ability of OD-PP@SeNPs hydrogels (n = 3)

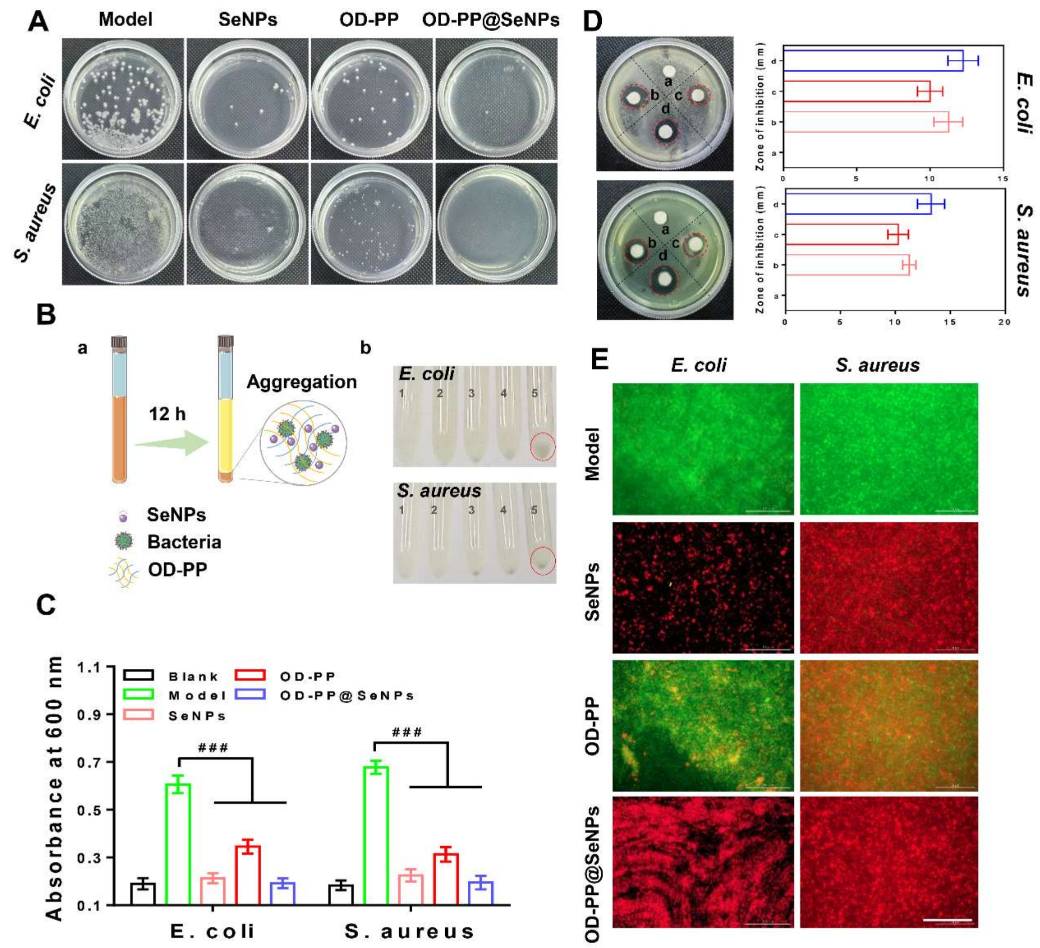


**Figure S18** Antibacterial activity evaluation of the hydrogel. (A) Representative images of *E. coli* and *S.aureus* clones on agar plates after incubating with the different preparation for 12 h. (B) Bacterial aggregation. (a) Schematic diagram of bacterial aggregation and (b) representative images of bacterial aggregation. (C) Antibacterial ratio of different preparation against two kinds of bacteria *via* OD method (n = 6). (D) The growth inhibition zones of the (a) Model, (b) SeNPs, (c) OD-PP, and (d) OD-PP-SeNPs against *E. coli* and *S.aureus*, respectively. And statistical analysis of the inhibition zones of the different preparation against the two kinds of bacteria (n = 3). (E) CLSM images of the two bacterial strains with SYTO9/PI dual fluorescent staining after 12 h of co-incubation with different preparation. Note that all bacteria

alive were stained by SYTO9 and exhibited green fluorescence, while the dead bacteria with damaged membrane were stained by PI and presented red fluorescence. Scale bar = 30 μm.

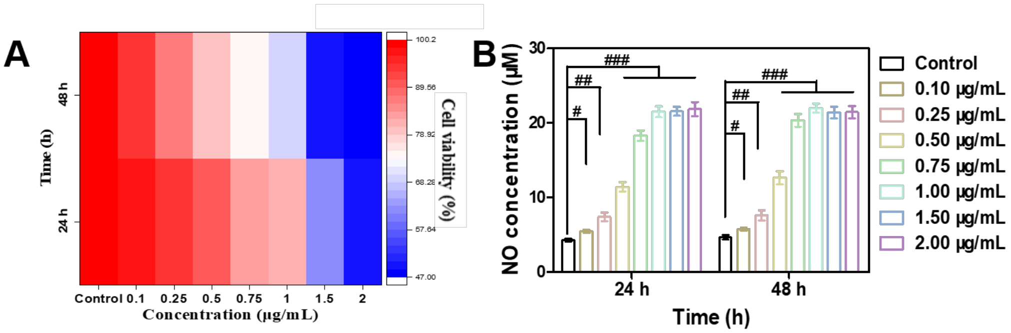


**Figure S19** Concentration screening of LPS. (A) RAW264.7 cells viability (n = 6) and (B) NO concentration (n = 6)

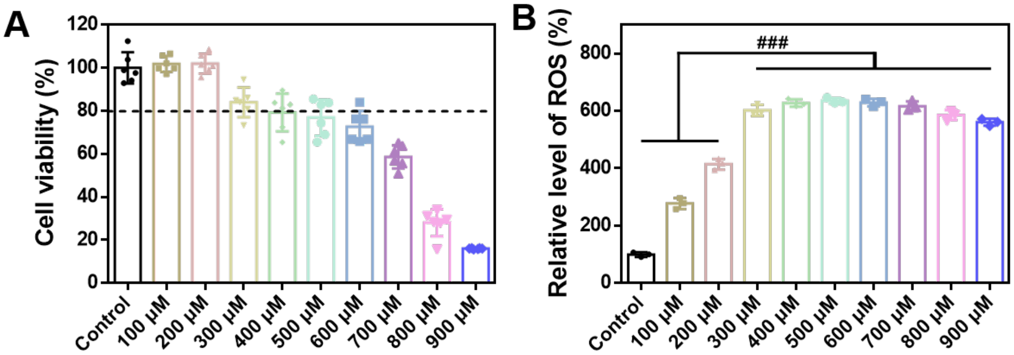


**Figure S20** Concentration screening of $H_2O_2$. (A) HUVECs cells viability (n = 6) and (B) relative level of ROS (n = 6)

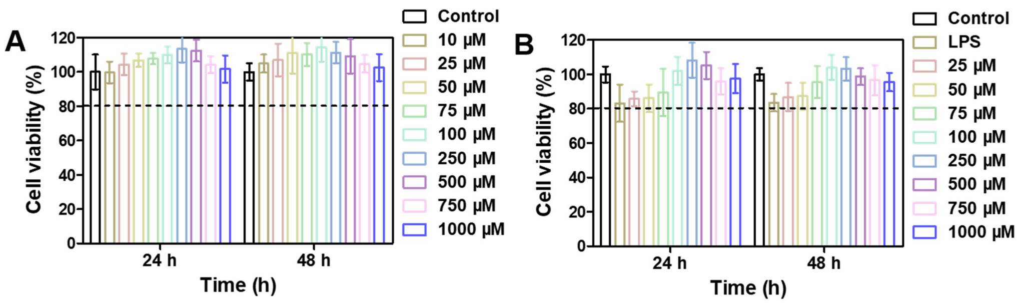


**Figure S21** Cells viability. (A) Different concentrations of SeNPs on RAW264.7 cells (n = 6). (B) Different concentrations of SeNPs on LPS-induced RAW264.7 cells (n = 6)

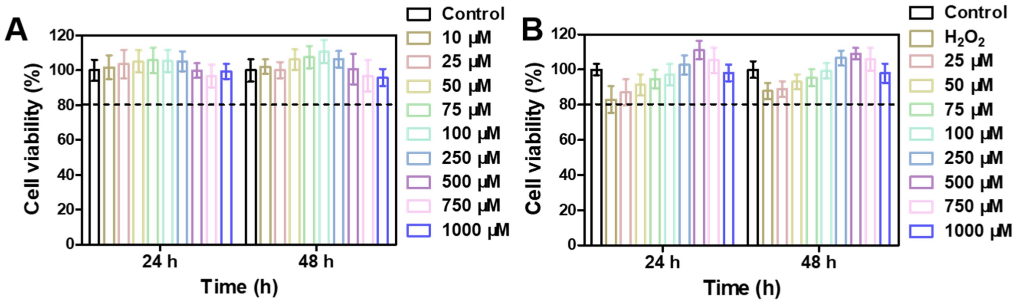


**Figure S22** Cells viability. (A) Different concentrations of SeNPs on HUVECs cells (n = 6). (B)Different concentrations of SeNPs on $H_2O_2$-induced HUVECs cells (n = 6)

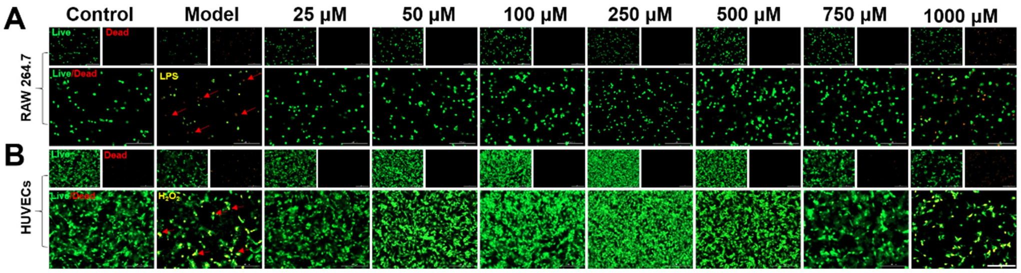


**Figure S23** Representative images of Live-dead cell staining. (A) Different concentrations of SeNPs on RAW264.7 cells. (B) Different concentrations of SeNPs on HUVECs cells. Scale bar = 200 μm.

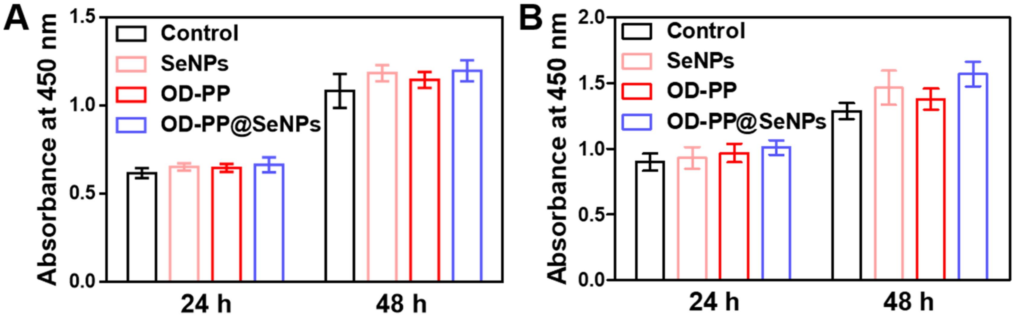


**Figure S24** Cells viability. (A) RAW264.7 cells (n = 6) and (B) HUVECs cells (n = 6)

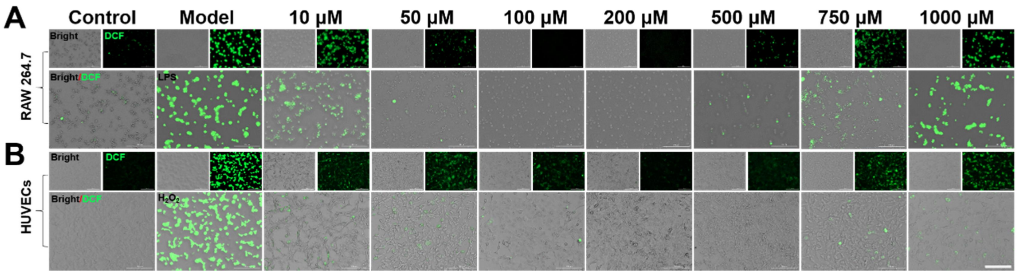


**Figure S25** ROS staining. (A) Different concentrations of SeNPs on RAW264.7 cells. (B) Different concentrations of SeNPs on HUVECs cells. Scale bar = 200 μm.

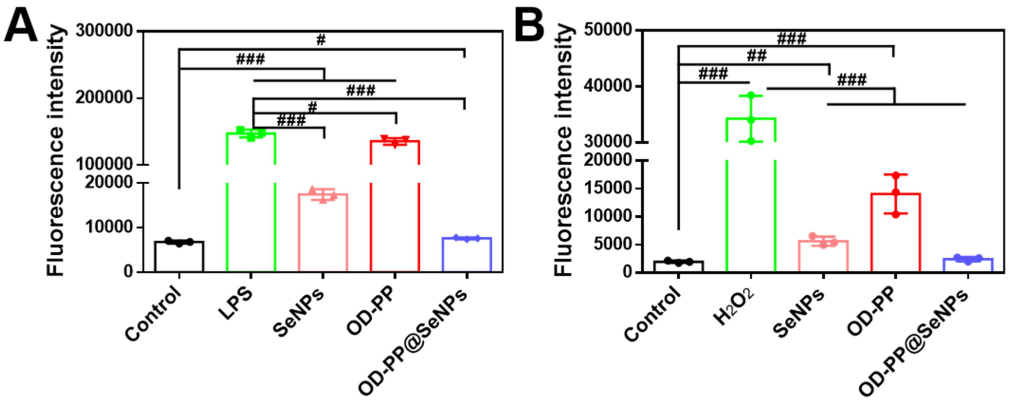


**Figure S26** Quantitative results of ROS by flow cytometry. (A) RAW264.7 cells (n = 3) and (B) HUVECs cells (n = 3)

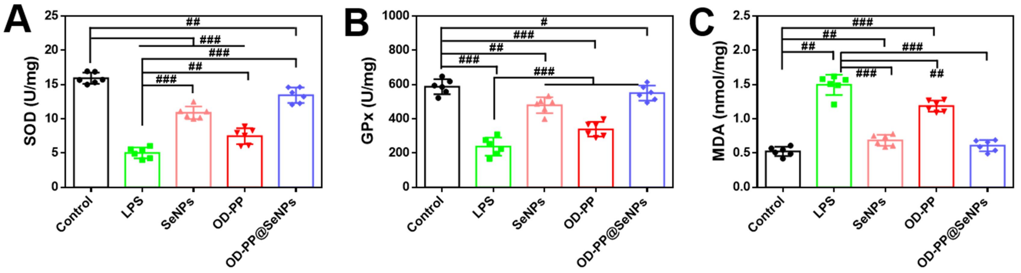


**Figure S27** Oxidative stress indexes in RAW264.7 cells. (A) SOD (n = 6), (B) GPx (n = 6), and (C) MDA (n = 6)

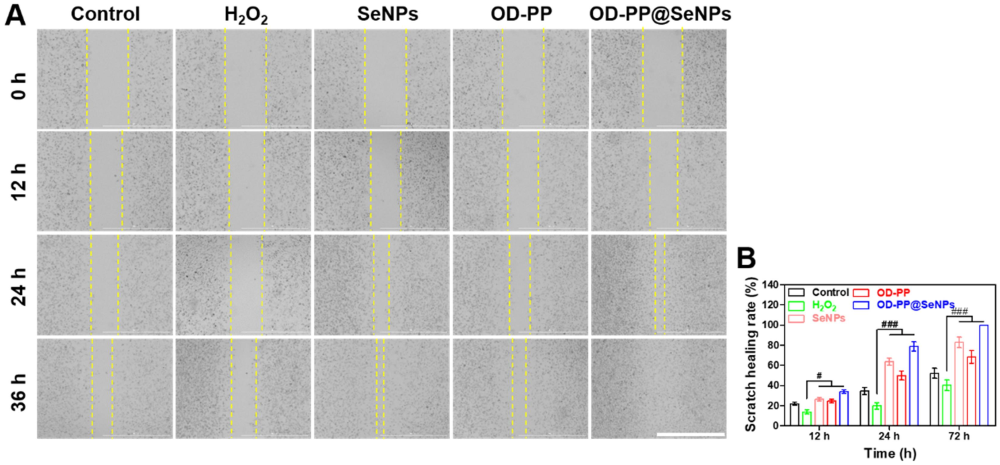


**Figure S28** Cell scratch. (A) Cell scratch healing rates during 36 h and (B) scratch healing rate (n = 3). Scale bar = 1000 μm.

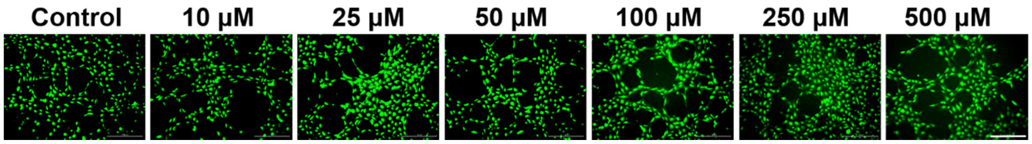


**Figure S29** The effect of different concentrations of SeNPs on the tube formation. Scale bar = 1000 μm.

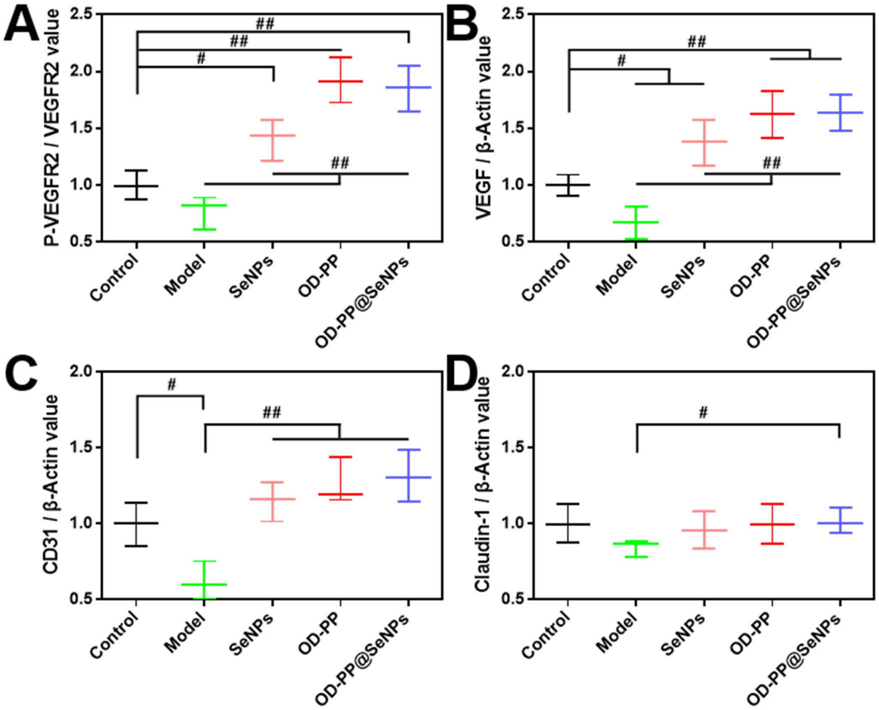


**Figure S30** Quantification of Western blot results. (A) VEGFR2, (B) VEGF, (C) CD31, and (D) Claudin-1

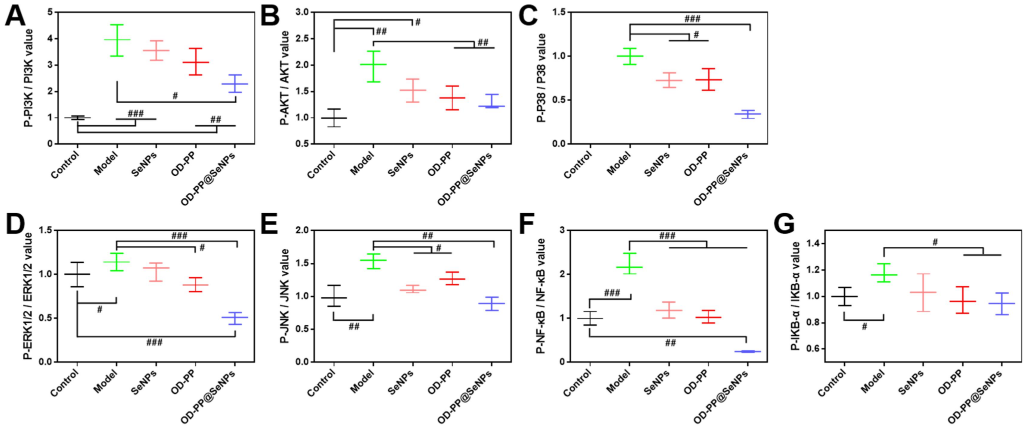


**Figure S31** Quantification of Western blot results. (A) P-PI3K/ PI3K, (B) P-AKT/ AKT, (C) P-P38/P38, (D) P-ERK1/2/ERK1/2, (E) P-JNK/ JNK, (F) P-NF-κB/ NF-κB, and (G) P-IκB-α/ IκB-α

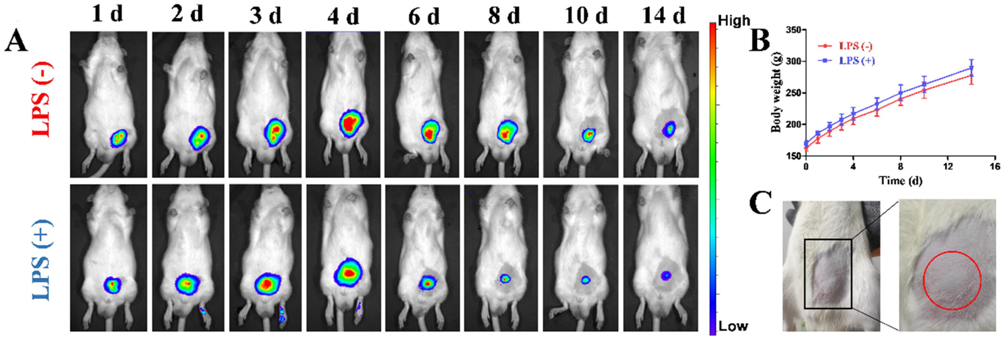


**Figure S32** Subcutaneous retention of OD-PP@SeNPs hydrogel. (A) Subcutaneous retention of OD-PP@SeNPs hydrogel loaded with IR820 by the small animal living imaging system. (B) Body weight of rats. (C) Photo of OD-PP@SeNPs hydrogel injected subcutaneously on the first day

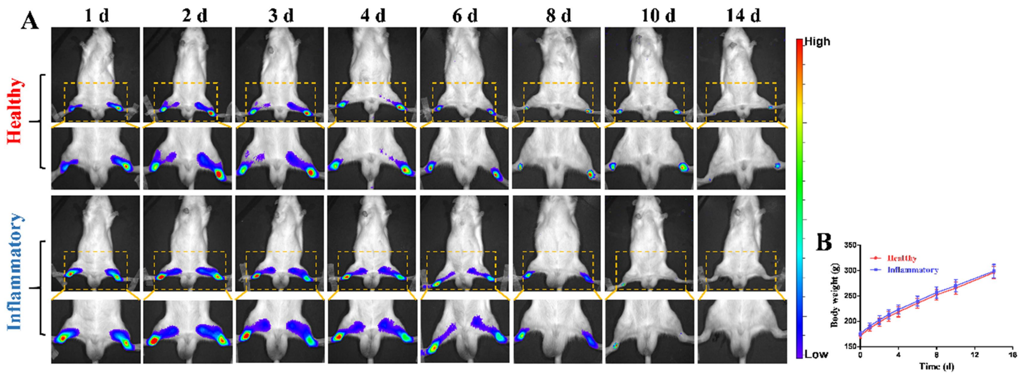


**Figure S33** Joint cavity retention of OD-PP@SeNPs hydrogel. (A) Joint cavity retention of OD-PP@SeNPs hydrogel loaded with IR820 by the small animal living imaging system and (B) Body weight

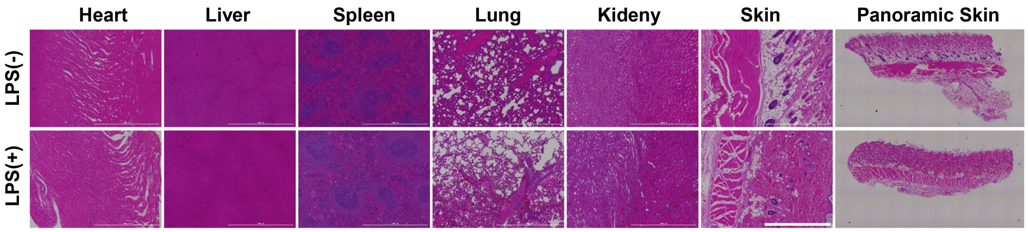


**Figure S34** H&E staining of major organs of rats at the end of subcutaneous retention experiment

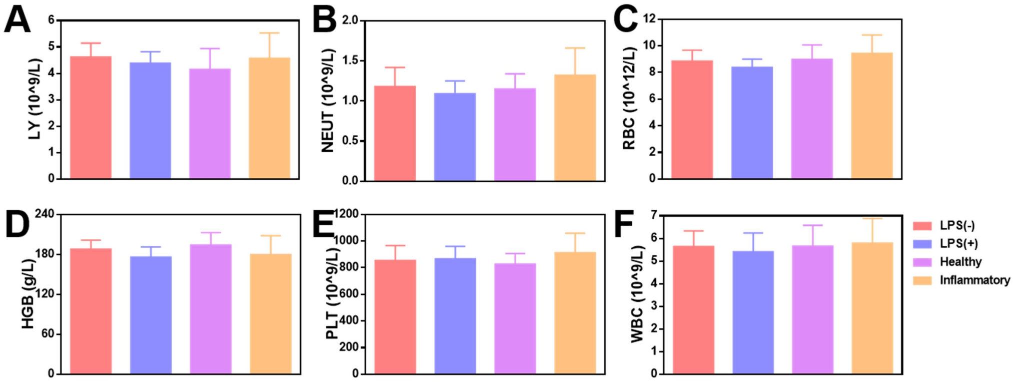


**Figure S35** Blood routine analysis of rats at the end of the *in vivo* retention experiment. (A) LY, (B) NEUT, (C) RBC, (D) HGB, (E) PLT, and (F) WBC

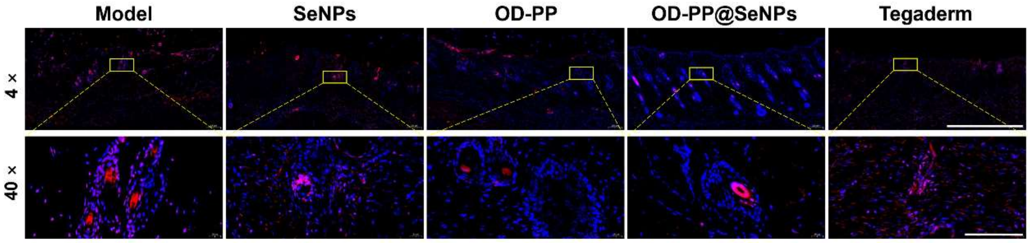


**Figure S36** ROS staining of skin wounds. Scale bar = 200 μm.

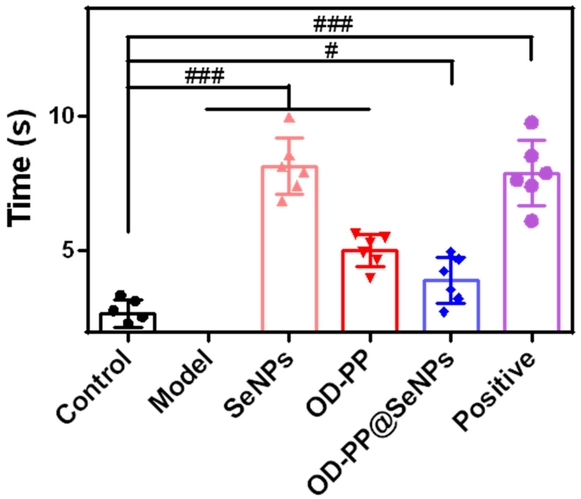


**Figure S37** The time for rats through the balance beam

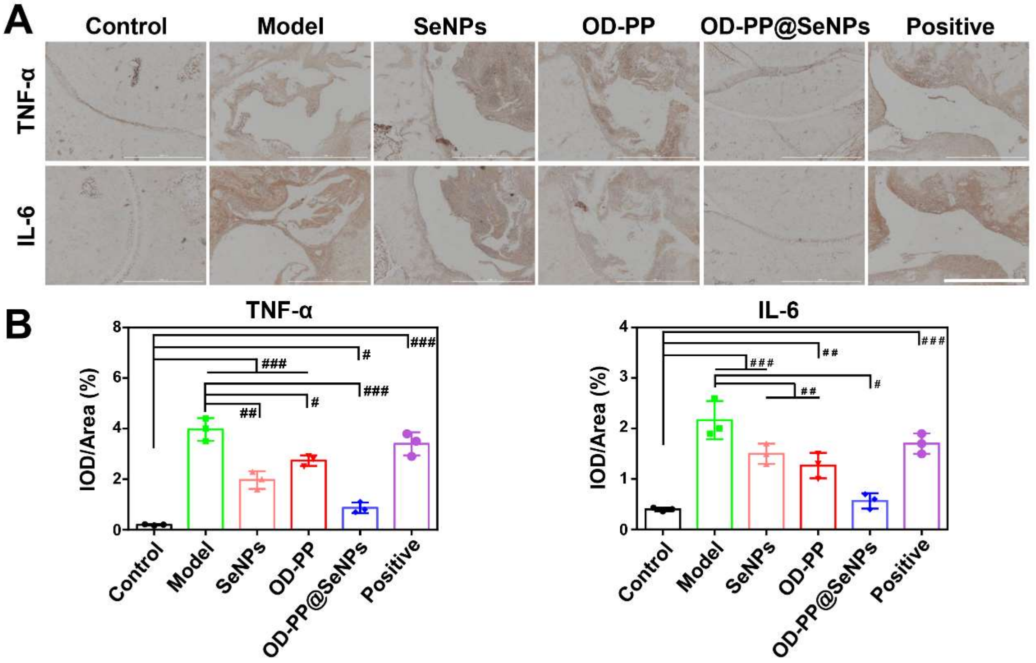


**Figure S38** Immunohistochemical of ankle joint in CIA rats. Scale bar = 1000 μm.

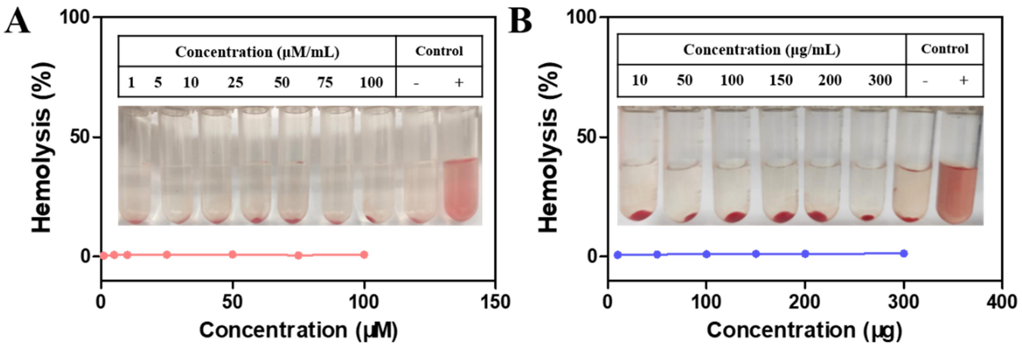


**Figure S39** Hemolysis assay with RBCs exposed to (A) SeNPs and (B) OD-PP@SeNPs

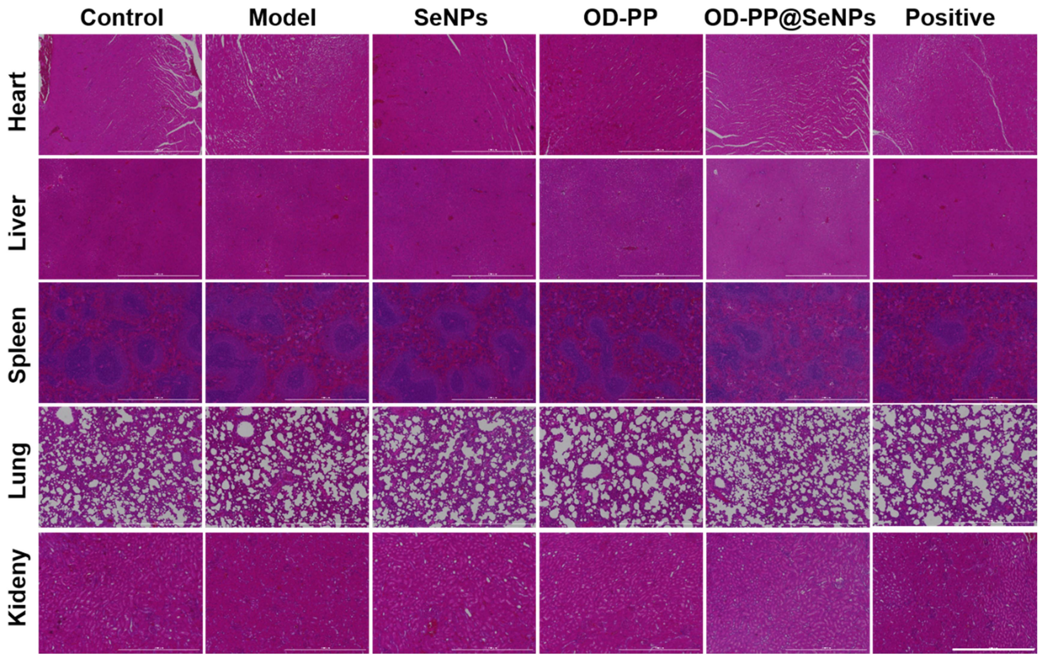


**Figure S40** H&E staining of rats in wound healing experiments. Scale bar = 1000 μm.

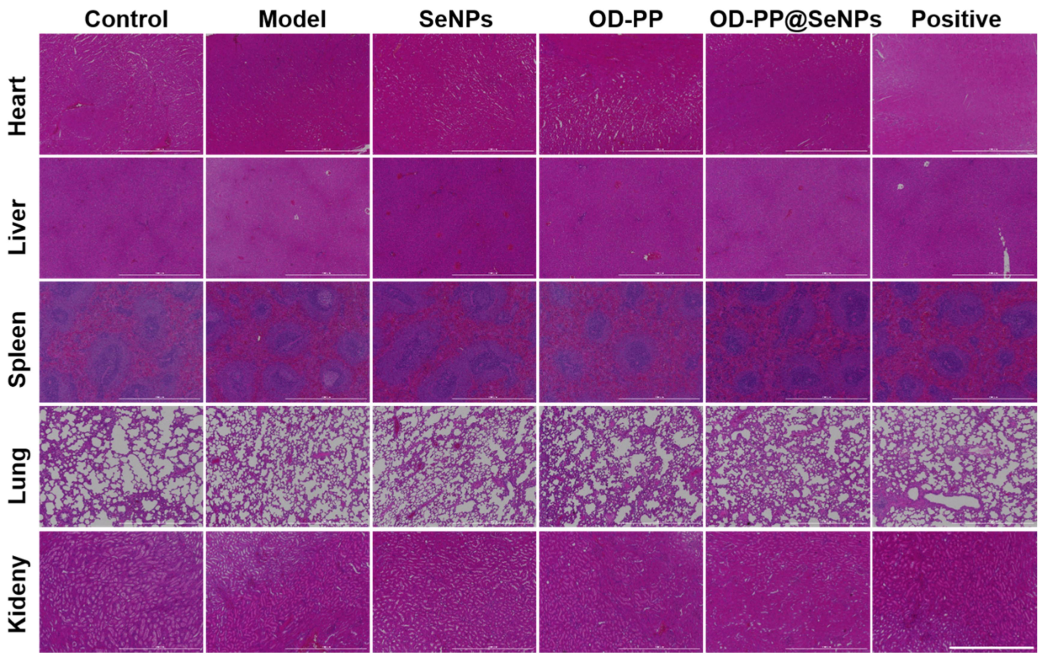


**Figure S41** H&E staining of rats in rheumatoid arthritis experiments. Scale bar = 1000 μm.

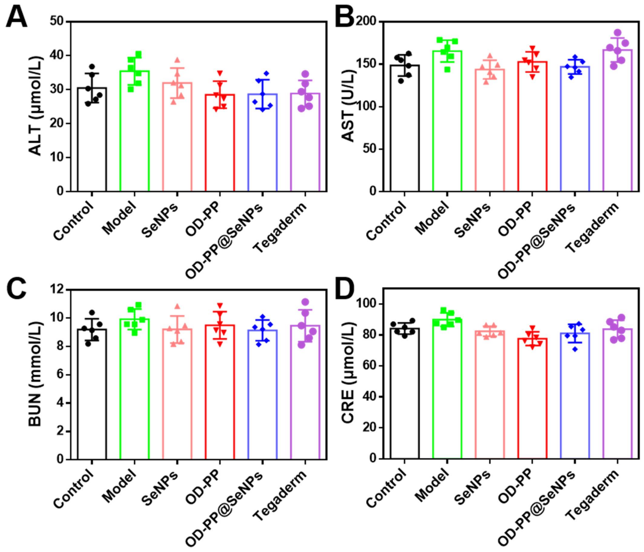


**Figure S42** Blood biochemistry of rats in wound healing experiment. (A) ALT, (B) AST, (C) BUN, and (D) CRE

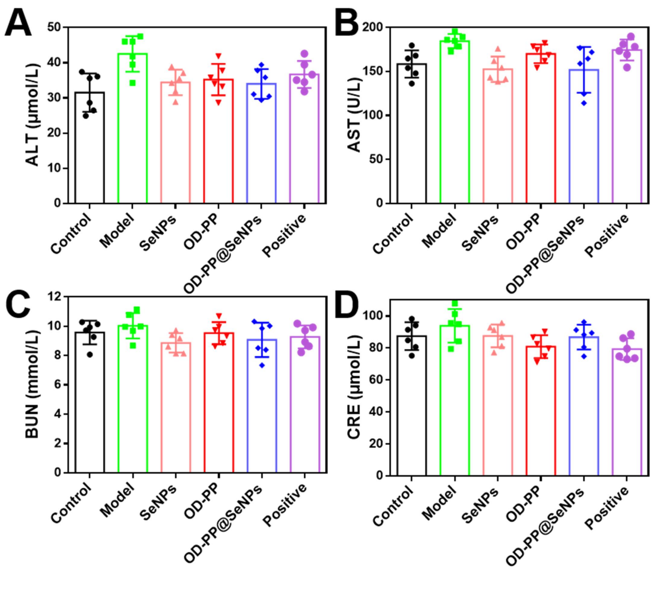


**Figure S43** Blood biochemistry of rats in rheumatoid arthritis experiment. (A) ALT, (B) AST, (C) BUN, and (D) CRE

**Table S1** Mathematical models of the regression for *in vitro* release profiles of preparations

| Hydrogel | Dissolution conditions | Zero order | First order | Higuchi | Korsmeyer-Peppas | |
|---|---|---|---|---|---|---|
| | | $R^2$ | $R^2$ | $R^2$ | $R^2$ | n |
| **OD-PP@BSA-FITC** | pH 7.4 | 0.60 | 0.96 | 0.92 | 0.84 | 0.30 |
| | pH 6.5 | 0.63 | 0.97 | 0.89 | 0.86 | 0.37 |
| | 1 mM $H_2O_2$ | 0.57 | 0.96 | 0.87 | 0.82 | 0.33 |
| | pH 6.5+1 mM $H_2O_2$ | 0.52 | 0.99 | 0.91 | 0.80 | 0.26 |
| **OD-PP@SeNPs@BSA-FITC** | pH 7.4 | 0.61 | 0.97 | 0.88 | 0.84 | 0.35 |
| | pH 6.5 | 0.75 | 0.98 | 0.90 | 0.91 | 0.49 |
| | 1 mM $H_2O_2$ | 0.75 | 0.97 | 0.89 | 0.80 | 0.50 |
| | pH 6.5+1 mM $H_2O_2$ | 0.76 | 0.96 | 0.90 | 0.89 | 0.53 |
| **OD-PP@SeNPs** | pH 7.4 | 0.76 | 0.98 | 0.93 | 0.92 | 0.47 |
| | pH 6.5 | 0.80 | 0.97 | 0.92 | 0.92 | 0.55 |
| | 1 mM $H_2O_2$ | 0.79 | 0.96 | 0.90 | 0.92 | 0.55 |
| | pH 6.5+1 mM $H_2O_2$ | 0.77 | 0.97 | 0.90 | 0.91 | 0.53 |

**Table S2** Blood Routine Test

| Indicators | Unit | LPS (-) | LPS (+) | Healthy | Inflammatory |
|---|---|---|---|---|---|
| **LY%** | % | 75.54±5.82 | 76.84±6.42 | 78.21±4.52 | 79.42±5.89 |
| **NEUT%** | % | 16.82±5.49 | 18.27±2.45 | 17.64±3.18 | 18.98±3.52 |
| **HCT** | % | 47.82±2.91 | 45.68±3.94 | 48.82±4.72 | 46.78±4.91 |
| **MCV** | Fl | 55.80±0.56 | 56.81±0.87 | 54.92±0.92 | 57.18±1.01 |
| **MCH** | Pg | 21.49±2.81 | 22.64±2.27 | 20.67±2.08 | 22.89±3.19 |
| **MCHC** | g/L | 325.81±20.76 | 334.37±22.23 | 349.81±25.16 | 342.62±23.86 |
| **RDW-SD** | Fl | 34.87±2.28 | 35.68±2.47 | 36.82±2.09 | 36.41±2.67 |
| **RDW-CV** | % | 18.85±0.99 | 19.24±1.05 | 17.94±1.27 | 19.48±1.67 |
| **MPV** | Fl | 7.48±0.59 | 7.65±0.62 | 7.97±0.73 | 7.81±0.82 |
| **PDW** | % | 11.48±0.82 | 10.97±0.95 | 11.68±0.72 | 12.47±0.96 |
| **PCT** | % | 0.68±0.19 | 0.66±0.28 | 0.74±0.17 | 0.71±0.24 |
| **P-LCR** | % | 12.97±1.98 | 13.58±2.28 | 14.18±2.77 | 13.89±2.51 |

**Abbreviations:**

WBC: white blood cell count; LYM: lymphocytes; NEUT: neutrophils; LY%: lymphocyte proportion; RBC: red blood cell; HGB: hemoglobin; HCT: hematocrit; MCV: mean corpuscular volume; MCH: mean corpuscular hemoglobin; MCHC: mean corpuscular hemoglobin concentration; RDW : red cell distribution width; RDW-SD: standard deviation in red cell distribution width; RDW-CV: coefficient variation of red blood cell volume distribution width; PLT: platelet count;MPV: mean platelet volume; PDW: platelet distribution width; PCT: thrombocytocrit; P-LCR: platelet large cell ratio.

**Table S3** Mathematical models of commonly used drug release kinetics

| Model | Mathematical equation | Y axis | X axis | Slope |
|---|---|---|---|---|
| **Zero order** | $Q_t = k_0t+Q_0$ | $Q_t$ | t | $k_0$ |
| **First order** | $Log\ Q_t = LogQ_0-k_0t/2.303$ | $Log\ Q_t$ | t | $k_0/2.303$ |
| **Korsmeyer-Peppas** | $M_t/M_\infty = ktn$ | $Log\ (M_t/M_\infty)$ | Log t | n |
| **Higuchi** | $Q_t = kHt^{1/2}$ | $Q_t$ | $t^{1/2}$ | kH |

**Table S4** Primer sequences used for RT-PCR

| Gene | Sequences |
|---|---|
| *tnf-α* | Forward: GCTCCCTCTCATCAGTTCCA |
| | Reverse: GCTTGGTGGTTTGCTACGAC |
| *il-1β* | Forward: TCTGAAGCAGCTATGGCAAC |
| | Reverse: TCAGCCTCAAAGAACAGGTCA |
| *il-6* | Forward: AACGAAAGTCAACTCCATCTG |
| | Reverse: GGTATCCTCTGTGAAGTCTCC |
| *il-10* | Forward: AGAGCCACATGCTCCTAGA |
| | Reverse: CCTGCATTAAGGAGTCGGTTAG |
| GAPDH | Forward: AGTGCCAGCCTCGTCTCATA |
| | Reverse: TGAACTTGCCGTGGGTAGAG |

**Table S5** Clinical score for RA

| **Arthritis scores** | **The incidence of arthritis** |
|---|---|
| **0** | Normal |
| **1** | Mild, but definite redness and swelling of the ankle or wrist, or apparent redness and swelling limited to individual digits |
| **2** | Moderate redness and swelling of ankle of wrist |
| **3** | Severe redness and swelling of ankle of the entire paw |
| **4** | Maximally inflamed limb with involvement of multiple joints |

## References


[1] H. Wang, X. Gong, Y. Miao, X. Guo, C. Liu, Y. Y. Fan, J. Zhang, B. Niu, W. Li, *Food Chem.* **2019**, *283*, 397.

[2] N. Jha, P. Esakkiraj, A. Annamalai, A. K. Lakra, S. Naik, V. Arul, *J. Trace Elem. Miner.* **2022**, *2*, 100019.

[3] X. Song, Y. Chen, H. Sun, X. Liu, X. Leng, *Food Chem.* **2020**, *331*, 127378.

[4] P. Kord Forooshani, R. Pinnaratip, E. Polega, A. G. Tyo, E. Pearson, B. Liu, T. O. Folayan, L. Pan, R. M. Rajachar, C. L. Heldt, B. P. Lee, *Chem. Mater.* **2020**, *32*, 8182.

[5] C. H. J. C. Ruifang Han, Yu Xiao, Qianqian Bai, *Acta Pharm. Sin. B* **2022**, DOI 10.1016/j.apsb.2022.07.009.

[6] J. Y. Ng, A. M. Azizudin, *Clin. Rheumatol.* **2020**, *39*, 2861.

[7] A. Rehman, P. John, A. Bhatti, *Nanomaterials* **2021**, *11*, 2005.

[8] J. Qin, X. Huang, N. Wang, P. Zhou, H. Zhang, Z. Chen, K. Liang, D. Gong, Q. Zeng, P. Niu, A. Chen, L. Yuan, Z. Yang, L. Su, N. Shen, J. Deng, D. Yu, *Clin. Transl. Immunol.* **2021**, *10*, e1338.

[9] A. Khurana, S. Tekula, M. A. Saifi, P. Venkatesh, C. Godugu, *Biomed. Pharmacother.* **2019**, *111*, 802.
